# Identifying Bottlenecks in the Photocatalytic Oxygen Evolution Reaction with Covalent Organic Frameworks

**DOI:** 10.1021/acs.chemmater.5c00804

**Published:** 2025-06-04

**Authors:** Stefan Trenker, Hugo A. Vignolo-Gonzalez, Andrés Rodríguez-Camargo, Liang Yao, Martijn A. Zwijnenburg, Bettina V. Lotsch

**Affiliations:** † 28326 Max Planck Institute for Solid State Research, Heisenbergstr. 1, 70569 Stuttgart, Germany; ‡ Department of Chemistry, University of Munich (LMU), Butenandtstr. 5-13, 81377 Munich, Germany; § Department of Chemistry, University of Stuttgart, Pfaffenwaldring 55, 70569 Stuttgart, Germany; ∥ Department of Chemistry, University College London, 20 Gordon Street, London, WC1E 6BT, U.K.

## Abstract

Covalent organic frameworks (COFs) have emerged as promising semiconducting materials for photocatalytic applications due to their large surface area, high crystallinity, and vast synthetic tunability. This is especially noticeable in the context of photocatalytic water splitting, where many COFs have been employed for the hydrogen evolution half-reaction. There, sacrificial reagents typically replace the kinetically demanding oxygen evolution half-reaction. On the contrary, only few reports focus on (sacrificial) water oxidation with COF photocatalysts. In most of these cases, cobalt species are employed as oxygen evolution cocatalyst, often with limited insight into their structure and detailed role in the catalysis. Herein, we use heterogenization of a molecularly defined iridium half-sandwich complex onto a bipyridine-based COF (Ir@TAPB-BPY COF) and provide detailed structural insights ensuring the integrity of the targeted cocatalyst. First, we demonstrate the retained catalytic activity of the anchored Cp*Ir­(III) motifs in chemical water oxidation experiments. In contrast, subsequent photocatalytic and electrocatalytic tests indicate that Ir@TAPB-BPY COF does not evolve oxygen and that careful control experiments have to be conducted in order to avoid false positive results, caused for example by the sacrificial electron acceptor. Using computational methods, we trace back the missing performance to thermodynamic and kinetic limitations of the employed systems. This work demonstrates the pitfalls associated with low-performing oxygen evolution photocatalysts as well as the indispensability of control experiments and their careful evaluation.

## Graphical abstract

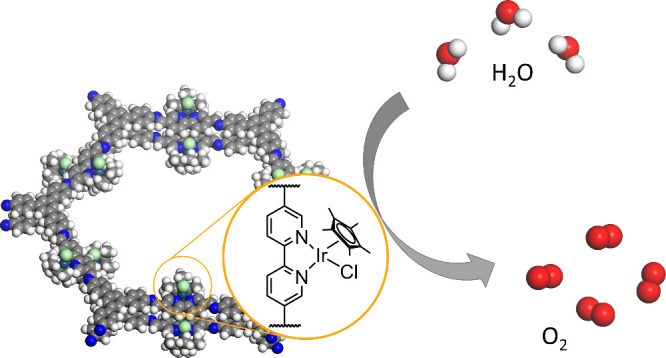

## Introduction

Covalent Organic Frameworks (COFs) are a new class of organic polymers that combine high crystallinity, defined porosity, and chemical stability.^1–6^ The structural modularity resulting from the use of tunable molecules as building blocks allows for the extensive and systematic alteration of the resulting materials’ chemical and optoelectronic properties. Recent examples include pore-size tuning through linker elongation,^7^ and introduction of redox activity based on suitable linkers.^8,9^ Special interest in COFs has been piqued for their potential as photocatalysts, e.g. for solar water splitting, which makes use of their intrinsic light absorption and high surface area, along with the molecular level tunability of the chromophoric units and thus optoelectronic properties.^10–13^ However, photocatalytic water splitting with COFs has generally focused on just the reductive half reaction, that is, hydrogen evolution (eq 1).^14^

1 $$4H^{+}+4e^{-}\to 2H_{2}$$

2 $$2H_{2}O\to O_{2}+4H^{+}+4e^{-}$$

In this case, the other half reaction of water splitting – the oxidation of water to dioxygen (eq 2) – is suppressed through the use of a sacrificial electron donor (SED), enabling mechanistic insights into and optimization of the hydrogen evolution reaction (HER, Scheme 1).^14,15^ For the photocatalytic HER to occur efficiently, COFs have been equipped with various cocatalytic species (proton reduction catalyst, PRC) such as Pt nanoparticles or cobalt complexes.^14,16^ By matching COF composition with the right choice of SED and cocatalysts, sacrificial hydrogen evolution rates as high as 289 mmol g^–1^ h^–1^ could be achieved to date.^17–28^

**Scheme 1:**
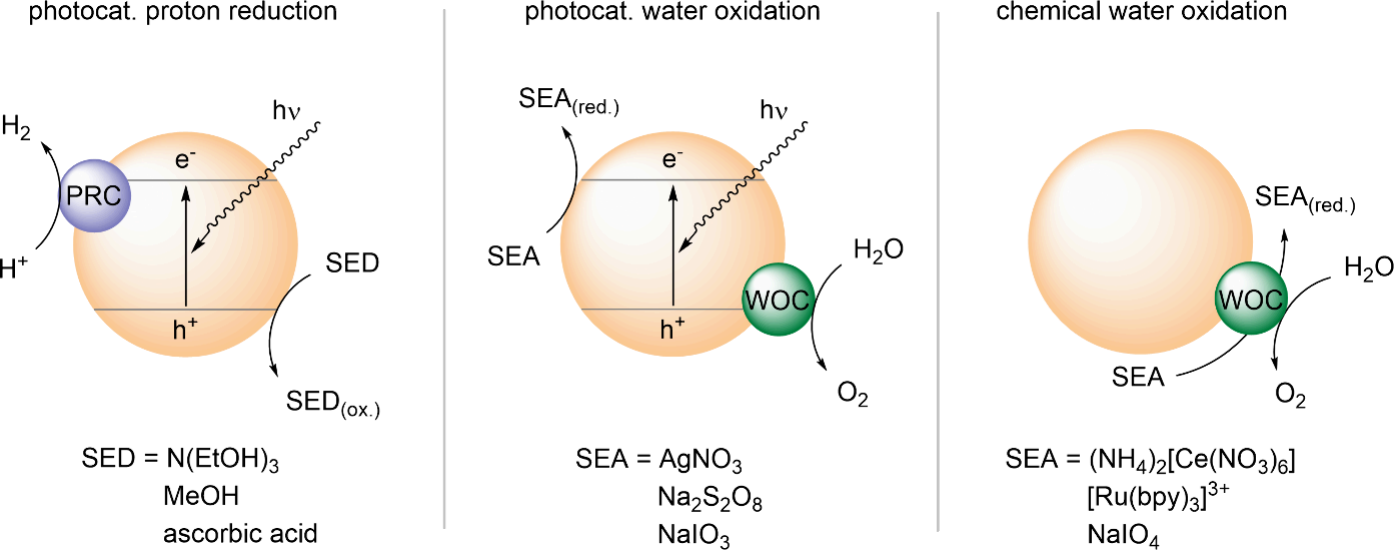
Sketch of Water Splitting Half Reactions with Semiconductor and Sacrificial Agents Compared to Chemical Water Oxidation

On the other hand, the oxygen evolution reaction (OER) is rarely explored with COFs.^29^ It requires a strongly positive valence band and involves transfer of four electrons along with the formation of an oxygen–oxygen bond, which makes it significantly more challenging compared to two-electron transfer HER, both thermodynamically and kinetically.^15,30–33^ Whereas the electrons are drawn off through reduction of water to hydrogen or a sacrificial electron acceptor (SEA), the electron holes are used to oxidize water. Given the standard potential for the oxidation of water to oxygen (E^0^ = 1.23 V vs. NHE), a sufficiently positive valence band is a major prerequisite for a semiconductor to be thermodynamically suitable for water oxidation photocatalysis. As shown both computationally and experimentally for conjugated polymers, electron-poor and nitrogen-rich monomers shift the ionization potential (valence band) to more positive values, in turn increasing the thermodynamic driving force for water oxidation with the eventual photocatalyst.^34–36^ In addition, to overcome the kinetic limitations associated with oxygen evolution, water oxidation catalysts (WOCs) are often employed. Traditionally, WOCs are composed of transition metals such as Ru, Ir, Co, Ni, either as heterogeneous oxide-based catalysts^37,38^ or in the form of molecular species.^32,39–41^

To avoid photophysical bottlenecks, screening and improvement of WOCs is often conducted with chemical oxidants in dark water oxidation schemes (Scheme 1).^42^ As a proof of concept, oxygen evolution with metal–organic frameworks (MOFs) has been achieved with cerium­(IV) ammonium nitrate (CAN) as the oxidant in a detailed mechanistic water oxidation study.^43,44^ In this example, Wang and co-workers synthesized UiO-67 with heterogenized half-sandwich IrCp* complexes as WOC (Cp* = pentamethylcyclopentadienyl).^43,44^ Similarly, Liang et al. reported the immobilization of a Ru­(terpy) complex on MIL-101­(Cr), and investigated the catalytic efficacy and stability depending on the binding site (terpy = 2,2′;6′,2″-terpyridine).^45^ However, in these examples the metalated MOFs acts as a heterogeneous support only, where CAN oxidizes the WOC directly. Also, experiments with CAN can only be conducted under strongly acidic conditions due to its instability above pH 1. Alternative terminal oxidants such as sodium periodate and potassium peroxymonosulfate can be employed under neutral conditions, but are prone to undesired oxygen transfer pathways.^42,46,47^ Recently, chemical water oxidation with CAN was reported for COFs constructed from catalytically active Ru linkers.^48,49^

Research on COFs for sacrificial photocatalytic water oxidation has so far focused on cocatalysts based on cobalt (Table S 9).^50–58^ The highest oxygen evolution rate yet was achieved using COF nanosheets composed of cobalt porphyrin (CoTPP) and cobalt bipyridine units (CoBpy_3_). When conducting photocatalytic experiments with AM1.5-filtered light and under reduced pressure, Zhou et al. detected oxygen evolution rates as high as 7323 μmol g^–1^ h^–1^ with CoTPP-CoBpy_3_.^59^ In a similar approach, the groups of Li, Li, and Yang use an imine COF (Bp-COF) with integral bipyridine sites to bind Co (II) ions as cocatalysts for photocatalytic water oxidation.^50^ Just recently, the isoreticular triazine-based TAPT-Bpy COF was also reported to evolve oxygen under illumination in the presence of a SEA.^60^ In these three cases, the valence bands were determined to be sufficiently low-lying for water oxidation, and the heterogenized cobalt species were characterized in detail.

Until now, other reports on COFs for sacrificial water oxidation with visible light in the context of water splitting are less detailed. For example, g-C_40_N_3_–COF,^51^ g-C_54_N_6_ COF,^61^ g-C_52_N_6_ COF,^61^ and I-TST^52^ are also employed with cobalt species as potential cocatalyst, but in parts exhibit only little characterization. Especially control experiments validating the integrity of the respective setups and the innocence of the SEA are rarely reported.^62^

In this work, we broaden the scope of oxygen evolution with COFs by developing a novel iridium-loaded COF and testing its activity with several commonly used SEAs in both photocatalytic and chemical water oxidation setups. Iridium, both heterogeneously in the form of its oxide and homogeneously as Ir complexes, is one of the most active elements for oxygen evolution catalysis.^63,64^ Among molecular Ir WOCs, iridium half-sandwich complexes with Cp* ligands have emerged as prototypical species for (photo)­chemical water oxidation^65–69^ as well as photoelectrocatalysis^70–74^ and electrocatalysis.^75–78^ In addition, IrO_2_-decorated photocatalysts have been reported for oxygen evolution from water both in the presence^79,80^ and absence^81^ of SEAs. In the latter case, Bai et al. could achieve overall water splitting with a conjugated polymer which emerged from both experimental and computational screening of the two water-splitting half-reactions.^34,81–84^

Since iridium is a scarce element, heterogenization of catalytically active mononuclear Ir complexes onto a suitable COF photocatalyst provides a means to achieve highest possible noble-metal atom utilization and, hence, atom effciency.^67,85,86^ Here, we first study chemical water oxidation to prove the retained catalytic activity of IrCp* when anchored to an imine COF *via* bipyridine sites. When testing the photocatalytic activity of the IrCp*-decorated COF though, we only find parasitic oxygen evolution that could be traced back to contaminated glassware or inherent instability of the SEAs based on rigorous blank experiments. Using both experimental and computational analysis, we identify thermodynamic bottlenecks as a source for the absence of oxygen evolution activity when using our iridium-loaded COF. Drawing on these results, our study provides a guideline for future research in the field of sacrificial oxygen evolution with heterogeneous organic photocatalysis.

## Results and Discussion

We choose TAPB-BPY COF as the model system for this study. It is formed through the condensation of 1,3,5-tris­(4-aminophenyl)­benzene (**TAPB**) and 2,2′-bipyridyl-5,5′-dialdehyde (**BPY-CHO**), which connect *via* the creation of imine bonds to form a two-dimensional COF under solvothermal conditions. Consequently, TAPB-BPY COF features an ordered, porous structure decorated with bipyridine moieties, which can be utilized to coordinate metal species in general, and water oxidation catalysts in particular.^87^ The latter has been shown by the groups of Li, Li, and Yang for the exact same COF (but denoted Bp-COF instead), where coordinating cobalt ions enabled photocatalytic water oxidation.^50^ According to the authors, this demonstrates that TAPB-BPY COF in principle has a low-lying valence band and is thus thermodynamically capable of oxidizing water (Table S 6).

Intrigued by these results, we expanded the scope of water oxidation cocatalysts used in conjunction with COFs. Making use of the bipyridine sites covering the pore walls of TAPB-BPY COF, we devised an iridium analogue of cobalt-loaded TAPB-BPY COF – Ir@TAPB-BPY COF – which features chelated IrCp* motifs (Figure 1). Whereas the molecularly defined Ir cocatalyst should introduce high catalytic activity, the surrounding COF provides both high spatial distribution and stability for the metal complex through its bipyridine sites.^85^ Besides constituting promising molecular iridium WOCs,^64^ IrCp* species are excellent catalysts for a variety of reactions such as organic oxidations^88^ and reductions,^89,90^ as well as CO_2_ hydrogenation.^91^ Altogether, these possibilities suggest the usability of Ir@TAPB-BPY COF for applications also beyond the oxygen evolution reaction covered within this report.

**Figure 1:**
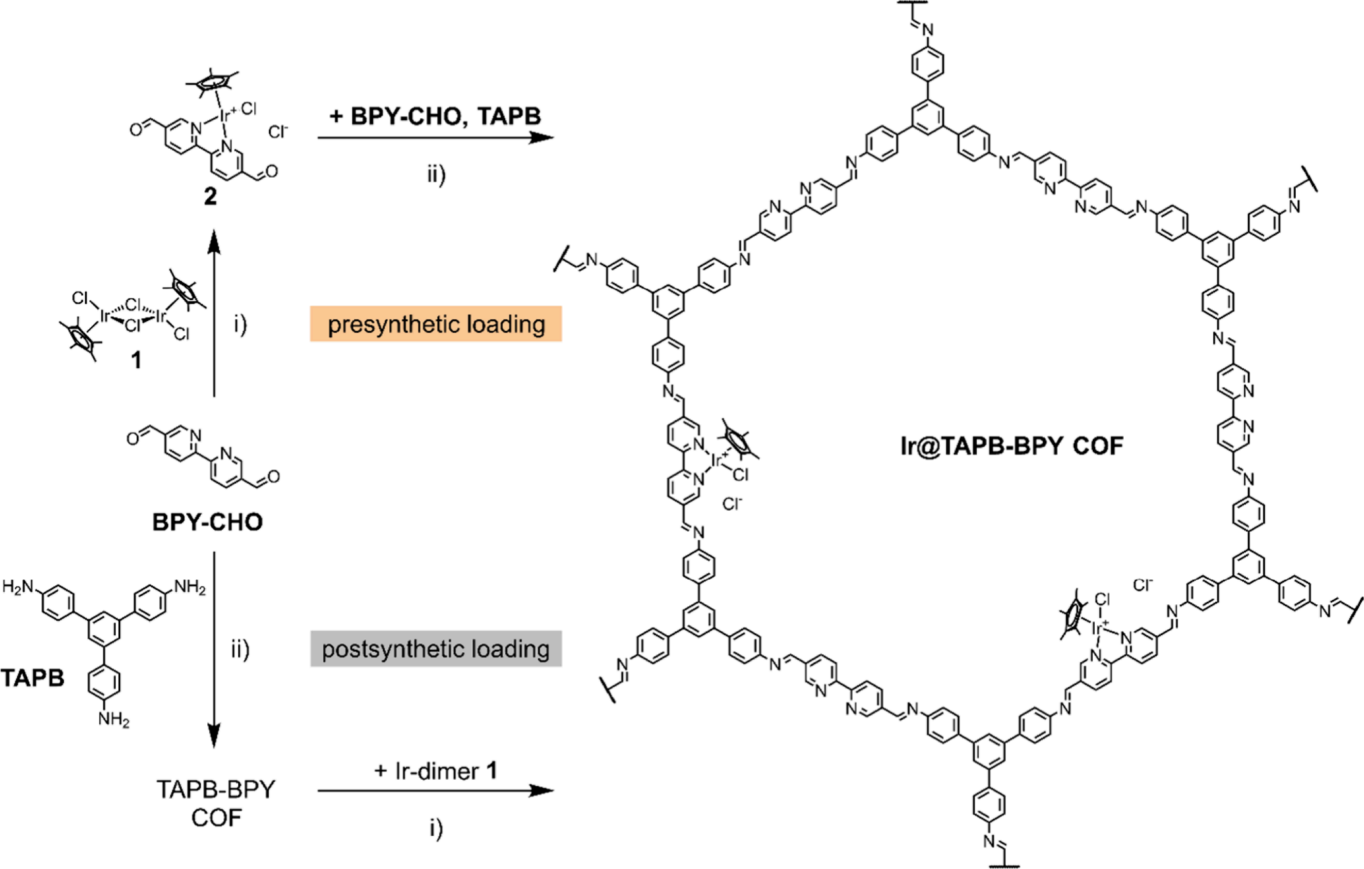
Synthetic approaches to IrCp*-loaded TAPB-BPY COF. Reaction conditions: (i) Solvent, 2–20 h, rt. (ii) Mesitylene/1,4-dioxane, 6M AcOH, 120 °C, 72 h.

The targeted iridium complex can be easily bound to the bipyridine moieties in TAPB-BPY COF through reaction with the respective dimer [Cp*IrCl_2_]_2_ **1**. Using an excess of **1**, a maximum loading of about 14 wt% could be achieved as confirmed by inductively coupled plasma optical emission spectrometry (ICP-OES). Scanning electron microscopy (SEM) confirms the homogeneous distribution of iridium over the COF particles (Figure S 3). However, we found that this procedure leads to a drastic decrease in crystallinity and porosity judging from diffraction patterns and sorption isotherms, respectively (Figure S 4, Figure S 5). Reactions of **1** with an isoreticular imine COF lacking bipyridine units confirm that Ir neither binds to the imine groups nor deposits in the form of nanoparticles (Figure S 6).^92^

In order to retain the crystalline and porous nature of TAPB-BPY COF after loading with **1**, we employed a presynthetic rather than the postsynthetic approach. By reaction of the original linker **BPY-CHO** with the iridium precursor **1**, we can synthesize the metalated building block **2** which can subsequently be used for the construction of iridium-loaded TAPB-BPY COF (Figure 1).^93–95^ Not only does this approach allow to precisely tune the iridium content of the resulting Ir@TAPB-BPY COF (Figure S 7), it also yields highly ordered materials judging from X-ray powder diffraction (XRPD) data (Figure 2a). Analogous to pristine TAPB-BPY COF, Ir@TAPB-BPY COF exhibits distinct reflections at 2θ = 2.29° (100), 4.06° (1–20), 4.64° (200), 6.21° (2–30), 8.26° (130) and a weak stacking reflection at 25.4° (001). We constructed a unit cell that resembles metal-free TAPB-BPY COF but is decorated with additional IrCp*Cl moieties occupying the bipyridine sites (space group *P*6_3_). This structural model comprises two nearly eclipsed layers with opposing bipyridine orientation and an IrCp*Cl fragment site occupancy of 0.1 to account for 10% (or 4.3 wt %) metalated linker **2** (Figure 2a). The unit cell parameters obtained from Pawley refinement (*R*_wp_ 7.91%) of the experimental powder pattern are *a* = *b* = 44.24 Å, *c* = 7.68 Å, α = 90°, β = 90°, γ = 120° (Figure S 9).

Comparing the connectivity, i.e. degree of condensation of both COFs *via* Fourier transform infrared (FTIR) spectroscopy, we could not detect significant differences (Figure S 10). Both Ir@TAPB-BPY COF and metal-free TAPB-BPY COF show the appearance of a new imine signal at 1623 cm^–1^ (ν_C=N(stretch)_), which – concomitant with the absence of amine bands (ν_N–H_ = 3200 – 3500 cm^–1^) – hints to the successful condensation of TAPB with **2** or **BPY-CHO**, respectively (Figure S 11).

Nitrogen sorption analysis reveals type IV isotherms for both metal-free and Ir@TAPB-BPY COF, though with varying BET surface areas of 1209 and 407 m^2^ g^–1^, respectively (Figure 2b).^96^ In a series with iridium content ranging from 2 to 6 wt %, however, we see no correlation of the loading degree with the surface area (Figure S 7). The derived pore size distributions (PSDs) for both pristine TAPB-BPY COF and Ir@TAPB-BPY COF show pores centered around 3.9 nm (Figure S 12).

**Figure 2:**
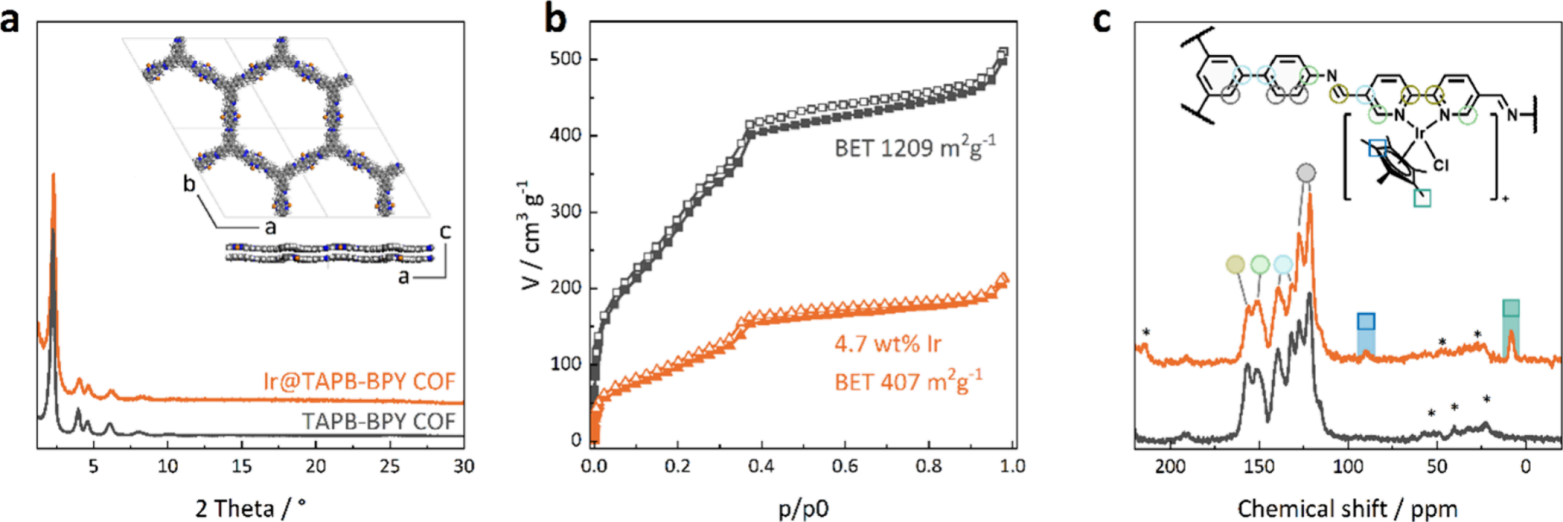
(a) XRPD pattern of TAPB-COF (gray) and Ir@TAPB-BPY COF (orange). Inset shows structural model for Ir@TAPB-BPY COF. (b) Nitrogen sorption isotherm of TAPB-BPY COF (gray) and Ir@TAPB-BPY COF (orange) at 77 K. Filled and open symbols represent the adsorption and the desorption branches, respectively. (c) ^13^C ssNMR spectrum of TAPB-BPY COF (gray) and Ir@TAPB-BPY COF (orange) with signal assignment. Asterisks mark spinning side bands.

Transmission electron microscopy (TEM) visualizes the mesopores inherent to Ir@TAPB-BPY COF, and Fast-Fourier Transform (FFT) analysis confirms a periodicity of 3.6 nm in good agreement with sorption and XRPD data (Figure S 14). We could not find any indication of metal-containing (nano)­particles covering the COF. Likewise, scanning electron microscopy (SEM) and elemental mapping show agglomerated micrometer-sized particles with an even distribution of N, C, Ir, and Cl, which indicates successful heterogenization of the targeted iridium complex onto TAPB-BPY COF (Figure S 15, Figure S 16).

^13^C solid-state nuclear magnetic resonance (ssNMR) was then conducted to assess the presence of the Cp* ligand and thus the integrity of the target complex. The COF’s aromatic signals in the range between 160 and 110 ppm do not change significantly upon Ir loading (Figure 2c). On the other hand, two new signals at 90 and 8 ppm can be assigned to the aromatic carbon atoms and methyl groups of the Cp* ligand, respectively. Concomitantly, a new signal at 1.3 ppm in the ^1^H ssNMR spectrum of Ir@TAPB-BPY COF shows the presence of the methyl protons (Figure S 18).

Furthermore, we conducted X-ray photoelectron spectroscopy (XPS) to confirm both the binding of the iridium species to the bipyridine sites of the heterogeneous support as well as its chemical state. The Ir 4f_7/2_ peak of Ir@TAPB-BPY COF is detected at 62.7 eV, which is in good agreement with literature values (62.7 eV)^97^ for the Ir­(III) center of [Cp*Ir­(bpy)]Cl (Figure S 20). Upon iridium incorporation, the nitrogen 1s signal for the imine and pyridyl nitrogen atoms shifts from 398.7 to 399.0 eV, indicative of the binding of Ir to the framework.

### Chemical Water Oxidation

In order to ascertain the retained activity of the COF-bound Ir species, we performed chemical water oxidation experiments in a specifically designed flow reactor (Figure 3).^98^ By using a continuous flow of inert gas with a slight overpressure of up to 250 mbar, we circumvent the leaking of oxygen into the apparatus and achieve high sensitivities. Online optical trace oxygen sensors allow for almost lag-free and instrumentally simple oxygen detection. Together, these factors allow us to conduct economical small-scale experiments, namely 5 mg COF dispersed in 5 mL sacrificial solution for a typical experiment. We previously discussed the layout and reliability of this setup.^98,99^

In a typical experiment with CAN as the terminal oxidant, we suspended the COF in 0.1 M nitric acid (pH 1). After a blank injection of HNO_3 (aq.)_ to evaluate the amount of trace oxygen introduced per injection, we added a degassed portion of CAN through a gastight septum injector nut (Figure 3). As expected, both pristine aqueous nitric acid as well as nonmetalated TAPB-BPY COF do not show significant oxygen evolution upon CAN addition (Table 1, entries 1 + 2, Figure S 22).

**Table 1:** Chemical Water Oxidation Experiments with Ir@TAPB-BPY COF^a^

Entry	Catalyst	μmol Ir	[Ir], μM^b^	O_2_ evolution^c^	TOF,^c^ h^–1^
1	HNO_3_ blank	-	-	0.111 μmol	-
2	TAPB-BPY COF	-	-	0.161 μmol	-
3	Ir@TAPB-BPY COF	0.25	50	0.327 μmol	2.52
4	Ir@TAPB-BPY COF	0.48	96	0.765 μmol	3.18
5	Ir@TAPB-BPY COF	1.68	336	3.457 μmol	4.08
6	[Cp*Ir(bpy)Cl]Cl	0.25	50	26.864 μmol	214
7	[Cp*Ir(bpy)Cl]Cl	0.025	5	2.654 μmol	212
8^d^	[Cp*Ir(bpy)Cl]Cl (lit.)	0.025	5	1.625 μmol	130
9^e^	[Cp*Ir(bpy)Cl]Cl (lit.)	0.30	7.1	37.8 μmol	252

a Reaction conditions: 5.0 mg COF in 78 mM CAN (in 5.0 mL 0.1 M HNO_3_).

b [Ir] represents the concentration of iridium complex in the reaction mixture and is derived either from the initial amount of molecular catalyst or the Ir loading of the COF as determined *via* ICP.

c After/within 30 min.

d Value extracted from graph. ref. 76

e Data obtained in experiments with 28 mM CAN and volumetric detection. TOF value based on data extracted from graph. ref. 101.

Iridium species, including [Cp*Ir­(bpy)­Cl]­Cl, have in fact been reported as water oxidation catalyst under these exact conditions.^76,100^ Indeed, we found that Ir@TAPB-BPY COF shows substantial oxygen evolution, with turnover frequencies (TOFs) in the range of 2.52 – 4.08 h^–1^ depending on the Ir loading (Table 1, entries 3–5, Figure 3). Also, Ir@TAPB-BPY COF exhibits turnover numbers of >1 and continuous oxygen evolution over two hours (Figure S 23), which hints to a catalytic OER process instead of a degradation process releasing O_2_. These results indicate the general preservation of catalytic activity of the Cp*Ir moiety bound to TAPB-BPY COF, despite showing activity two orders of magnitude lower than the molecular WOC in both our own (Table 1, entries 6 + 7, Figure S 23) and literature experiments (Table 1, entries 8 + 9). Note, however, that the molecular catalyst is completely soluble in the reaction mixture, hence higher activity for the homogeneous as compared to the heterogeneous catalyst system is expected.

**Figure 3:**
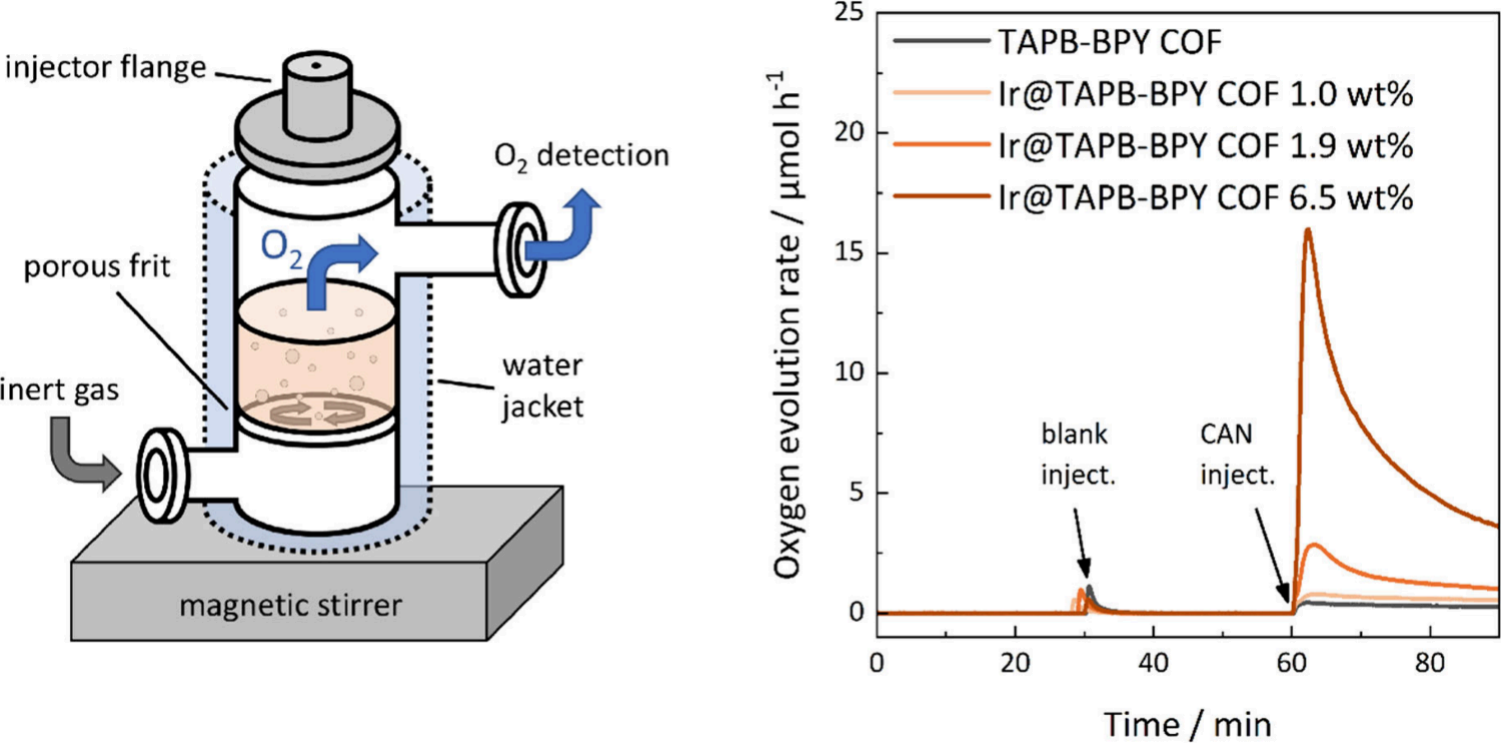
Left: Schematic experimental setup for the chemical water oxidation experiments. Right: Chemical water oxidation experiments with TAPB-BPY COF at varying iridium loadings.

SEM elemental mapping suggests that the uniform distribution of both Ir and Cl over Ir@TAPB-BPY COF is preserved after catalysis with CAN, but also deposition of Ce on the COF is observed (Figure S 25, Figure S 26). TEM analysis confirms the presence of CeO_*x*_ nanoparticles, but at the same time refutes the presence of iridium-rich oxidic nanoparticles which could also be catalytically active (Figure S 27).^101–103^ This is further corroborated by XPS analysis, which shows unchanged Ir peak positions and no signs of IrO_*x*_ (Figure S 30).^44,102^ The signals for the Ce depositions are characteristic for Ce­(III) species, in line with the expected reduction of CAN during catalysis (Figure S 31). A filtration experiment shows negligible oxygen evolution for the reaction filtrate compared to suspended Ir@TAPB-BPY COF (Figure S 32) ruling out that the observed activity is due to desorbed catalyst.

NMR spectroscopy of the filtrate after water oxidation catalysis with Ir@TAPB-BPY COF in 78 mM CAN shows the presence of formic acid and acetic acid indicative of the oxidative cleavage of the Cp* ring (Figure S 33).^44,101,103^ It has been proposed elsewhere that the oxidation or complete loss of the Cp* ligand does not impair the catalytic activity of Ir-based WOCs, and that some bidentate ligands such as bipyridine do not degrade.^44,103–108^ Also in accordance with literature reports on molecular [Cp*Ir­(bpy)­Cl]­Cl, Ir@TAPB-BPY COF shows the highest catalytic activity around CAN concentrations of 50 mM (Figure S 34–Figure S 37).^76^

Unfortunately, ligand degradation is not the only drawback of chemical water oxidation with CAN in acidic media. XRPD of the retrieved Ir@TAPB-BPY COF indicates a loss of long-range order after treatment with CAN, especially at higher concentrations (Figure S 38). Consistently, nitrogen sorption analysis and TEM reveal the gradual loss of structural porosity when increasing the CAN concentration from 10 mM to 78 mM (Figure S 39, Figure S 27). FTIR spectroscopy hints to oxidation of either the framework or the Cp* ligand, as the broad band around 3300 cm^–1^ is assigned to O–H stretching modes (Figure S 40).^44,102,103^ More importantly, FTIR spectroscopy shows the partial loss of imine stretching vibrations originally present in Ir@TAPB-BPY COF, while at the same time we observe a new feature around 1654 cm^–1^ which we ascribe to amide groups resulting from imine oxidation through CAN.^109^ Likewise, postcatalytic ssNMR spectroscopy reveals the loss of the ^13^C imine signal at 156 ppm, concomitant with the appearance of a weak amide signal at 167 ppm (Figure S 41).^109^ Besides, both the signals of the COF backbone as well as the Cp* ring are preserved, showing that neither is completely decomposed.

Though driving WOC with CAN allows for a fast assessment of the catalytic activity of COF-bound iridium WOCs, the acidic and highly oxidative conditions during catalysis are detrimental to the COF stability. This is less problematic when switching to photocatalytic water oxidation since the respective SEAs can usually be used under neutral conditions and have less positive redox potentials (Figure S 42). That said, photocatalytic water oxidation is mechanistically more complex, as it relies on successful charge carrier generation within the COF upon illumination, and transport of the electron holes to the active sites.

### Photocatalysis

The catalytic activity of Ir@TAPB-BPY COF for photocatalytic oxygen evolution was examined in the same flow reactor after replacing the injector nut with an optical quartz glass window (Figure S 43).^98^ After exemplarily confirming the functionality of our setup with the literature-known catalytic systems [Cp*Ir­(bpy)­Cl]Cl + [Ru­(bpy)_3_]­Cl_2_ and RuO_2_ + TiO_2_ (Figure S 44), we conducted photocatalytic experiments with Ir@TAPB-BPY COF in the presence of sodium persulfate as SEA.^98,110^ Unfortunately, we could neither detect significant amounts of oxygen under illumination with AM 1.5-filtered nor with visible light (>420 nm, Figure S 52). Only with full-spectral illumination could we measure oxygen evolution over the course of 14 h with rates of up to 0.75 μmol h^–1^ (150 μmol h^–1^ g^–1^) (Figure S 53). However, we do not attribute these findings to actual water oxidation by the COF, but rather to decomposition of S_2_O_8_^2–^ as discussed in detail in the Supporting Information (section S6).

We thus investigated silver nitrate as an alternative SEA, which is more commonly used in water oxidation photocatalysis but brings about the disadvantage of undesired deposition of elemental silver.^62^ Over the course of a photocatalytic experiment this can significantly alter the optical properties of the photocatalyst.^82,111,112^ Due to the milder reaction conditions during photocatalysis compared to chemical water oxidation, Ir@TAPB-BPY COF could be recovered with retained crystallinity and porosity, albeit showing deposited Ag/AgCl particles (Figure S 45–Figure S 51). XPS confirms the deposition of silver species (Figure S 31) and at the same time reveals unchanged iridium binding energy, indicating the preservation of anchored iridium complexes (Figure S 30). More importantly though, numerous photocatalysis experiments revealed that Ir@TAPB-BPY COF does not evolve oxygen upon illumination in the presence of AgNO_3_ as SEA. Due to the extensive scope of this study, we shifted the discussion of the AgNO_3_ results to the Supporting Information (section S7). In a nutshell, there we explore undesired decomposition pathways of silver species, and how improper experimental setup such as contaminated glassware can lead to unexpected oxygen evolution and data misinterpretation. This is particularly relevant for photocatalytic reactor components such as glass frits that are used for various types of catalytically active materials (Figure S 66). An exemplary oxygen trace for an experiment with Ir@TAPB-BPY COF illustrates the congruence with a blank measurement and thus the inactivity of the COF in the presence of AgNO_3_ under typical conditions (Figure 4).

**Figure 4:**
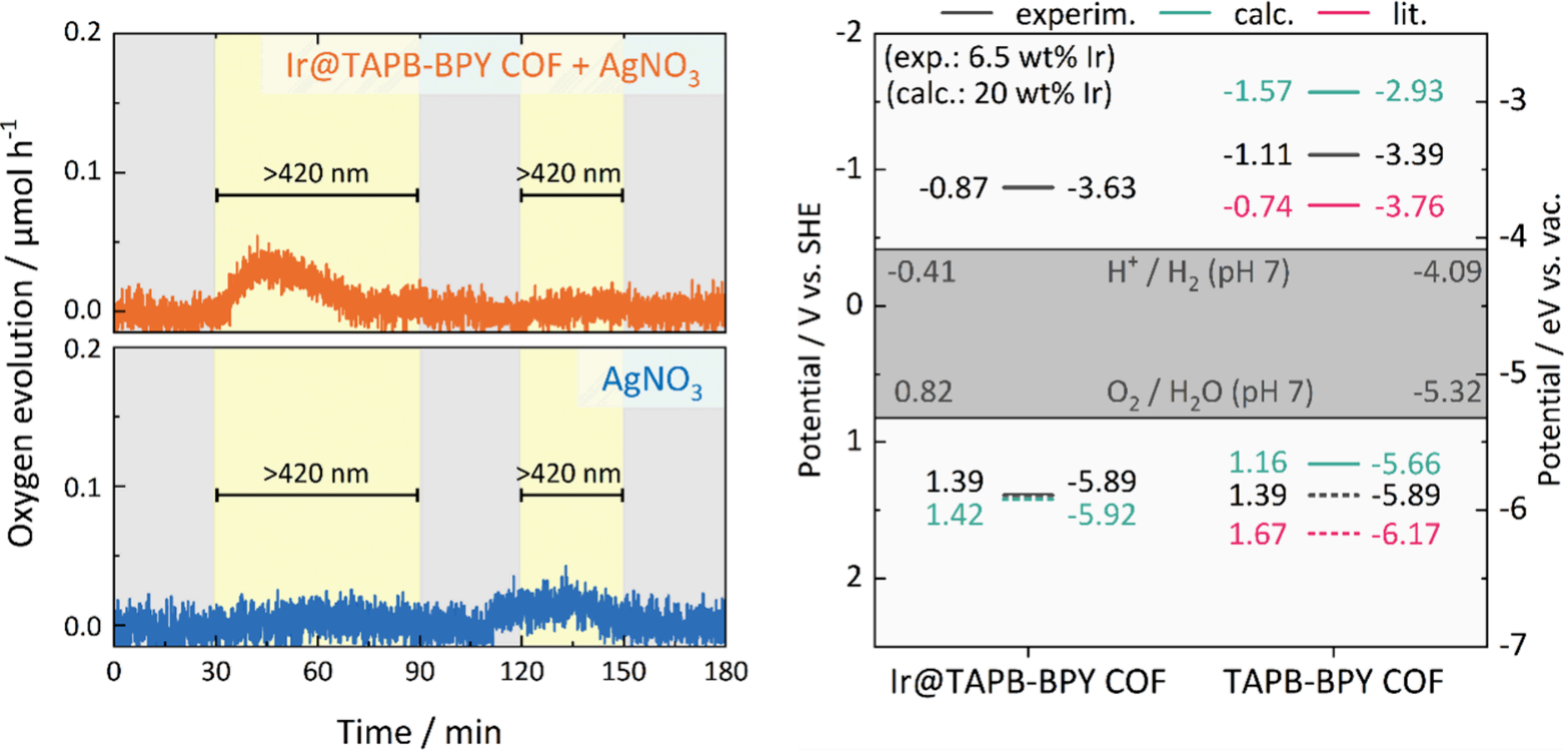
Left: Photocatalytic oxygen evolution experiment with Ir@TAPB-BPY COF (top) compared to blank measurement (bottom). Reaction conditions: 5.0 mg of Ir@TAPB-BPY COF (1.9 wt % Ir), AgNO_3_ (10 mM, 5 mL), 300 W Xe lamp, optical filters as specified. Gray areas represent dark reaction conditions. Right: Comparison of experimentally determined band positions (*via* CV in MeCN; black), computationally predicted ionization potentials and electron affinities (in MeCN, turquoise), and literature values (pink) for Ir@TAPB-BPY COF and TAPB-BPY COF. Dashed lines represent values obtained indirectly, for example E_VB_/VBM calculated from the E_CB_/CBM *via* subtraction of the optical band gap. Potentials for the water splitting half reactions illustrated as the gray area. See Table S 6 and Table S 7 for details.

In order to ensure that water oxidation catalysis is thermodynamically feasible with TAPB-BPY COF, we approximated the band positions through cyclic voltammetry (CV, Figure S 72). To this end, we determined the conduction band E_CB_ from irreversible reduction waves, and derived the valence band edge E_VB_ through subtraction of the optical band gap (see Supporting Information for details).^17,113^ Given a water oxidation potential of −5.32 eV vs.vac (0.82 V vs. SHE), TAPB-BPY COF (E_VB_ = −5.89 eV/1.39 V vs. SHE) should be thermodynamically capable of oxidizing water. These values also roughly match the reported band positions for this COF (Table S 6, Figure 4).

To complement the electrochemical assessment based on CV, we performed DFT calculations on representative cluster fragments of the TAPB-BPY COF (Figure S 73) embedded in a dielectric continuum to describe the effect of the water or other solvents in the pores of the COF and surrounding the COF particles. Though this approach does not take into account periodicity and layer stacking, it allows for an assessment of the effect of the dielectric screening of the water or other solvents the COF is dispersed in on its electronic properties.^22^ Previous work demonstrated that such DFT cluster calculations in the case of linear conjugated polymers accurately predict the ionization potential (IP) and electron affinity (EA) values of dry polymer solids measured by experimental photoelectron spectroscopy when using a relative dielectric permittivity of 2 (organic solid) for the continuum.^36,114^ When using a relative dielectric permittivity of 80.1 (water) DFT cluster calculations can successfully explain the trends in the activity of such polymeric solids for sacrificial hydrogen evolution from water/SED mixtures.^83,84,115–117^ For TAPB-BPY COF the IP and EA obtained with the B3LYP DFT functional^118–121^ are 1.16 and −1.57 V vs. SHE, respectively (in acetonitrile; for values in water see Table S 7). Despite showing more negative values compared to the experimentally determined band positions, these calculations confirm the expectation that TAPB-BPY COF should thermodynamically be suitable for water oxidation catalysis. We note, however, that especially the calculated IP hints at there only being a small driving force for the water oxidation reaction, which would make water oxidation challenging.

This statement also holds true for Ir@TAPB-BPY, for which CV reveals a valence band maximum identical to TAPB-BPY COF (1.39 V vs. SHE/–5.89 eV vs. vac.; Figure S 74, Table S 6) while DFT predicts a slightly more positive value than in the absence of Ir (1.42 V vs. SHE/–5.92 eV vs. vac). Interestingly, the underlying nonaqueous CV curves do not show any evidence of iridium redox features such as the Ir^II/III^ or the Ir^III/IV^ couple (Figure S 76).^68,76,97,122^ Also when conducting aqueous CV, Ir@TAPB-BPY COF does not show irreversible oxidation waves, in contrast to the molecular reference (Figure S 77). Similar observations have recently been made for other COFs loaded with molecularly defined iridium species.^123,124^ This could be a hint of strong electronic coupling between the heterogenized molecular iridium species and the TAPB-BPY COF, similar to that observed by Surendranath and co-workers for homogeneous complexes conjugated to graphite, called graphite-conjugated catalysts (GCC).^125^ We discuss the consequences of this concept for OER with Ir@TAPB-BPY in the Supporting Information (section S9). However, given the imperfect conjugation and limited crystallinity of imine-COFs, the absence of electrochemical features can also be indicative of a hindered electronic addressability of the COF-bound iridium motifs.

Intuitively, the direct coordination of the Cp*Ir moiety to the TAPB-BPY COF should lead to hybridization of the respective orbitals/bands as discussed above. If the potential of such a “hybrid band” is more positive than the water oxidation potential, i.e., providing enough overpotential, oxygen evolution would be thermodynamically feasible (scenario 1, Scheme 2). On the contrary, when considering the COF solely as photosensitizer and the iridium species as an energetically decoupled water oxidation catalyst in a heterojunction-type arrangement, the relative position of their potentials/bands determines whether water oxidation is thermodynamically feasible (scenario 2, Scheme 2) or not (scenario 3, Scheme 2). In all three scenarios, effective removal of electrons *via* a SEA is required to avoid charge recombination (not shown).

**Scheme 2:**
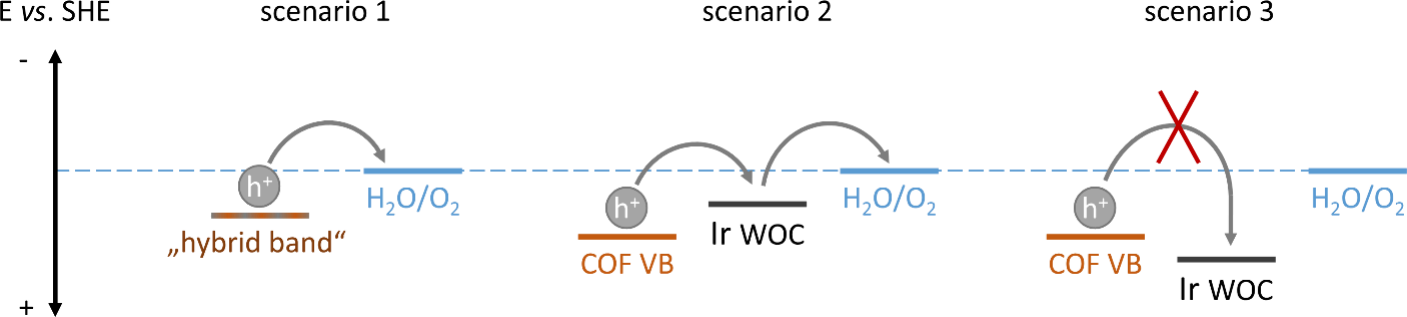
Simplified Thermodynamic Scenarios for the Photocatalytic Oxygen Evolution Reaction with Iridium-Loaded COFs

For electrocatalysis with Cp*Ir­(bpy) derivates immobilized on ITO electrodes, Joya et al. found the oxygen evolution onset potential to be 1.51 V vs NHE at pH 7. Neglecting slightly different ligand environments, this value suggests that photogenerated electron holes in Ir@TAPB-BPY COF (∼1.38 V vs SHE) do not provide enough driving force for water oxidation in heterojunction cases (scenario 3, Scheme 2).

Thus, the usage of COFs with even lower-lying valence bands seems promising. The isoreticular, triazine-based TAPT-BPY COF is reported to exhibit a significantly more positive E_VB_ of up to 2.28 V vs NHE (Figure S 79), and should therefore be able to oxidize water in both scenario 1 and 2 (Scheme 2). However, with iridium-loaded Ir@TAPT-BPY COF (Figure S 80) photocatalytic oxygen evolution is also not observed under identical conditions (Figure S 81). Likewise, trials using Ir@TAPB-BPY COF as photocatalyst for oxidations of thermodynamically and kinetically less demanding substrates such as methanol also failed (Table S 8).

We therefore conclude that highly positive valence bands are unconditional prerequisites for oxygen evolution with COF photocatalysts – but that successful catalysis cannot be inferred from low-lying valence bands alone. In other words, a positive valence band is a necessary, but not a sufficient condition for OER. Due to the slow kinetics and demanding mechanisms underlying the OER, photocatalytic water oxidation with COFs requires – compared to photocatalytic HER with COFs – significantly larger overpotentials of at least 300 mV.^14,126^ We note that apart from high overpotentials several other bottlenecks can limit photocatalytic oxygen evolution, which have not been discussed in detail within the scope of this work. These include efficient light absorption, charge separation and transfer, charge recombination, accessibility of active sites, and catalyst-substrate interactions.^29,86,127–129^ Especially the use of sacrificial electron acceptors can have a dramatic influence on charge separation and, hence, catalytic activity. As all of these aspects can vary in COFs from batch to batch, accurate experimentation and in-depth analysis are indispensable to ensure data integrity and reproducibility. Consequently, we were not able to reproduce sacrificial oxygen evolution with various cobalt-loaded COF photocatalysts as reported in the literature (see section S10).

## Conclusions

In summary, we successfully synthesized a new 2D COF equipped with Cp*Ir motifs and demonstrated catalytic activity for chemical water oxidation with CAN. There, however, the TAPB-BPY COF only acts as a support for the iridium WOC, and in addition cannot withstand the harsh reaction conditions over time. Subsequently, we used Ir@TAPB-BPY COF to broaden the scope to photocatalytic water oxidation with tailored organic frameworks. In a detailed photocatalytic study, we could not confirm Ir@TAPB-BPY COF to act as a photocatalyst for the OER but instead found that photocatalytic oxygen evolution requires careful experimentation to avoid data misinterpretation and false positive results. This demonstrates the importance of reporting negative results and suitable blank experiments.

By comparing chemical water oxidation and photocatalytic oxygen evolution experiments, we could identify thermodynamic limitations inherent to Ir@TAPB-BPY COF as a possible bottleneck in water oxidation. Only when using a strong chemical oxidant such as CAN we could achieve catalytic oxygen evolution by bypassing both the need for strongly oxidative photogenerated holes as well as their efficient transport to the active sites.

We thus recommend a best practice approach including not only the screening of different catalytic species and their loading, but also the systematic screening of SEAs, including chemical water oxidation reagents, to identify the best reaction conditions and rule out data misinterpretation by, for example, catalytically active contaminants or decomposition of sacrificial agents. Effectively, the present study further highlights the intricacy of photocatalytic OER in general as well as the utilization of COFs for OER in particular.

## Experimental Section

### Synthesis of Iridium-Loaded Bipyridine Linker **2**

Following a literature procedure^130^ [Cp*IrCl_2_]_2_ (199 mg, 0.25 mmol) and 2,2’-bipyridiyl-5,5′-dialdehyde (108 mg, 0.5 mmol) were dissolved in DCM (6 mL) and stirred at rt for 42 h. The resulting orange solution was filtered through a 0.45 μm PTFE syringe filter and evaporated using a stream of nitrogen, yielding **2** as an orange solid (284.8 mg, 93%). ^1^H NMR (400 MHz, Chloroform-*d*) δ 10.28 (s, 2H), 9.44 (d, *J* = 8.2 Hz, 2H), 9.25 (s, 2H), 8.68 (d, *J* = 8.1 Hz, 2H), 1.77 (s, 15H) ppm. ^1^H NMR (400 MHz, Methanol-*d*_4_, hydrate formation) δ 9.07 (d, *J* = 6.9 Hz, 2H), 8.60 (d, *J* = 9.9 Hz, 2H), 8.30 (d, *J* = 8.4 Hz, 2H), 5.81 (s, 2H), 1.71 (s, 15H). ^13^C NMR (101 MHz, Methanol-*d*_4_, hydrate formation) δ 156.0, 151.0, 144.0, 139.6, 124.8, 95.5, 91.0, 8.6 ppm. MS (ESI+): *m*/*z* calc. for C_22_H_23_ClIrN_2_O_2_^+^ (M-Cl^–^): 575.10718; found 575.10835. ICP: 33.49 wt % Ir; calc. 31.479 wt %.

### Synthesis of TAPB-BPY COF

A Biotage 20 mL microwave vial was charged with TAPB (92.3 mg, 0.252 mmol, 2.0 equiv) and 2,2’-bipyridyl-5,5′-dialdehyde (81.9 mg, 0.378 mmol, 3.0 equiv). The vial was temporarily sealed with a rubber septum and flushed three times *via* vacuum/argon cycles. Mesitylene (5.1 mL) and 1,4-dioxane (0.9 mL) were added, and the reactants were suspended *via* sonication for 5 min. The suspension was degassed *via* three vacuum/argon cycles. Aqueous acetic acid (600 μL, 6M) was added, the vial was sealed with a crimp cap and heated to 120 °C for 3 d. After cooling to room temperature, the solid was filtered off and washed with DMF (50 mL), THF (50 mL), acetone (50 mL), and MeOH (50 mL). Soxhlet extraction with MeOH overnight followed by supercritical CO_2_ drying yielded TAPB-BPY COF (118 mg, 76%) as an ocre powder. Elemental analysis calc. (%) for C_84_H_54_N_12_: C 81.93, H 4.42, N 13.65; found: C 79.38, H 4.59, N 12.72. TAPT-BPY COF was synthesized accordingly.

### Synthesis of Ir@TAPB-BPY COF

Ir@TAPB-BPY COF was synthesized according to the procedure described for TAPB-BPY COF but with substitution of bipyridyl-5,5′-dialdehyde by the desired amount of iridium-loaded linker **2**. The postsynthetic loading procedure is described in the Supporting Information.

### Chemical Oxygen Evolution

If not stated otherwise, 5.0 mg COF were suspended in 4.6 mL 0.1 M HNO_3_ and sonicated for 5 min. The resulting suspension was transferred to a custom-made flow reactor (Figure 3) and degassed in the dark with an argon flow of 40–60 NmL min^–1^ while stirring at 400 rpm. Once the system approached the baseline oxygen content, the flow was reduced to 20 NmL min^–1^ and the temperature of the water-jacketed reactor was kept at 25 °C. After adjusting the pressure to 1.10 – 1.25 bar, the baseline was measured for 30 min before injecting a blank (0.1 M HNO_3_, 0.2 mL) through a septum injector nut. After another 30 min, 0.2 mL of a CAN stock solution (1.95 M, in 0.1 M HNO_3_) were injected, yielding a final CAN concentration of 78 mM. The oxygen evolution rate was determined every three seconds using a PreSens flow-through cell with an integrated PSt-6 or PSt-9 sensor spot connected to a Fibox 4 trace oxygen meter. The readout in ppm was converted to μmol h^–1^ by applying a factor of 0.0749 μmol h^–1^ ppm^–1^.

### Photocatalytic Oxygen Evolution

If not stated otherwise, 5.0 mg COF were suspended in the respective aqueous reaction medium (5 mL) and sonicated for at least 10 min. The resulting suspension was transferred to a custom-made flow reactor (Figure S 43) and the sacrificial electron acceptor was added. The reactor was closed and the reaction mixture was degassed in the dark with an argon flow of 40–60 NmL min^–1^ while stirring at 400 rpm. Once the system approached the baseline oxygen content, the flow was reduced to 20 NmL min^–1^ and the temperature of the water-jacketed reactor was kept at 25 °C using a JULABO FP50-ME thermostat. After adjusting the pressure to 1.10 – 1.25 bar, the baseline was measured for 30 min before starting the illumination from above through a quartz glass blind flange. The oxygen evolution rate was determined every three seconds using a PreSens flow-through cell with an integrated PSt-9 sensor spot connected to a Fibox 4 trace oxygen meter. The readout in ppm was baseline-corrected and subsequently converted to μmol h^–1^ by applying a factor of 0.0749 μmol h^–1^ ppm^–1^ (Figure S 43). To prevent uncontrolled heating of the oxygen sensor during illumination, it was covered in wet paper towels together with the Pt100 temperature sensor. If not stated or depicted otherwise, illumination was performed for 90 min. In between measurements, the reactor was cleaned with aqua regia, piranha solution, and copious amounts of water.

### Material Characterization

As-synthesized and post-catalytic samples were characterized by an array of analytical techniques which are detailed in the Supporting Information. Similarly, details on the use of computational chemistry for band position calculation are given in the Supporting Information.

## Supplementary Material





## Notes

Synthetic details, material characterization (SEM, TEM, EDX, XRPD, ssNMR, IR, UV–vis, XPS, gas sorption), electrochemical measurements, photocatalytic and chemical oxygen evolution experiments, paragraphs on photolytic sodium persulfate decomposition and photoreactor contamination, reproduction of literature reports, photographs, and DFT results (PDF)
Structural data relevant to computational chemistry (ZIP)
