# Supplementary material for: Identifying Bottlenecks in the Photocatalytic Oxygen Evolution Reaction with Covalent Organic Frameworks
Source: Chem Mater. 2025 Jun 4;37(12):4463–74. doi: 10.1021/acs.chemmater.5c00804 (PMC12199468; doi:10.1021/acs.chemmater.5c00804)
Supplement: Supplementary file 1 [file cm5c00804_si_001.pdf]

Supporting Information

Identifying bottlenecks in the photocatalytic oxygen evolution reaction with Covalent Organic Frameworks

Stefan Trenker, [a,b] *Hugo A. Vignolo-Gonzalez,* [a] *Andrés Rodríguez-Camargo,* [a,c] *Liang Yao,* [a] *Martijn A. Zwijnenburg,* [d] *Bettina V. Lotsch* [a,b,c]*

[a] Max Planck Institute for Solid State Research, Heisenbergstr. 1, 70569 Stuttgart, Germany.

[b] Department of Chemistry, University of Munich (LMU), Butenandtstr. 5-13, 81377 Munich, Germany.

[c] Department of Chemistry, University of Stuttgart, Pfaffenwaldring 55, 70569 Stuttgart, Germany

[d] Department of Chemistry, University College London, 20 Gordon Street, London, WC1E 6BT, UK.

Table of Contents

S1 - Instruments and Methods 3
S2 - Syntheses 8
S3 - COF Characterization 10
S4 - Chemical Water Oxidation 21
S5 - Photocatalytic Oxygen Evolution 33
S6 - Photocatalytic Oxygen Evolution Experiments with Sodium Persulfate as SEA 40
S7 - Silver Nitrate Decomposition 43
S8 - Electrochemistry and Computational Chemistry 55
S9 - Reflections on the GCC Concept 61
S10 - Photocatalysis Reproduction 65
References 85

S1 - Instruments and Methods

ICP-OES

ICP-OES spectroscopy was performed on a Varian Vista Pro and evaluated using the Agilent ICP Expert software. Samples were digested in concentrated nitric acid (65%) at 185 °C for 25 min with a CEM Discover SP-D.

Elemental analysis

Elemental analysis (C, H, N) was conducted on an ELEMENTAR vario EL using Helium as carrier gas.

Supercritical CO_2 drying

COF samples were kept soaked in ethanol prior to supercritical CO_2 extraction on a Leica EM CPD300 critical point dryer with ethanol as exchange liquid.

Nuclear magnetic resonance spectroscopy

Spectra of soluble samples were recorded using a Bruker AV400TR or a Jeol Eclipse 400+ spectrometer. Chemical shifts are denoted on the scale in parts per million (ppm), calibrated to residual non-deuterated solvent (^{1}H-NMR: 7.26 for $CDCl_3$, 2.50 for DMSO-d_6) or solvent carbon resonances (^{13}C-NMR: $CDCl_3$: 77.16 for $CDCl_3$, 39.52 for DMSO-d_6). Multiplicities are denoted as: s = singlet, d = duplet, t = triplet, q = quartet, m = multiplet, or as a combination thereof. Spectra were analyzed and processed using MestReNova version 10.0.2-15465.

Solid-state nuclear magnetic resonance experiments were performed on Bruker Neo 600 MHz instrument using a 4 mm outer diameter ZrO_2 rotor and a BL4 MAS double resonance probe at a spinning frequency of 14 kHz. The ^{13}C spectra were acquired with ^{1}H cross-polarization.

X-ray photoelectron spectroscopy (XPS)

XPS measurements were conducted on KRATOS Axis Ultra X-ray photoelectron spectrometer with a monochromatic Al Kα source and charge compensation. Binding energies were calibrated to the adventitious carbon 1s peak at 284.80 eV or the graphitic carbon (C=C) 1s peak at 284.5.[1–3] The CasaXPS software 2.3.16 was used for data analysis. Powder samples were measured on indium foil.

Physisorption analysis

Argon and nitrogen sorption measurements at 87 K and 77 K, respectively, were performed with a Quantachrome Instruments Autosorb iQ MP. Samples of more than 20 mg were preheated *in vacuo* (10^{-7} mbar) at 120 °C for 12 h. ASiQwin Version 3.01 was used for data analysis. Pore size distributions were evaluated using the carbon QSDFT kernel for cylindrical pores for the adsorption branch if not stated otherwise. Expected pore sizes were derived from structural models in Materials Studio v6.0.0.

Mass spectrometry

Experiments were performed on a Thermo Finnigan MAT 90 or MAT 95 mass spectrometer using electrospray ionization (ESI). m/z values were calculated using Perkin Elmer ChemDraw® Professional Version 16.0.0.82 (68).

Infrared spectroscopy

Infrared spectroscopy was conducted using a Perkin Elmer Spektrum BX II FT-IR equipped with an ATR unit (Smith Detection Dura-Sample IIR diamond). Background correction was done before sample measurements.

UV-Vis

Diffuse reflectance UV-Vis spectra were collected on a Cary 5000 spectrometer and referenced to barium sulfate. Absorption spectra were calculated from the reflectance data using the KUBELKA-MUNK function.

X-ray powder diffraction (XRPD)

XRPD patterns were collected at room temperature on a BRUKER D8 Discovery with Ni-filtered CuKα-radiation (1.5406 Å) and a position-sensitive LynxEye detector in Bragg-Brentano geometry. Materials Studio v6.0.0 was used for structural modelling, XRD pattern simulations, and Pawley Refinement.

High angle measurements for the identification of different silver species were performed on a STOE Stadi P powder diffractometer with Ge(111)-monochromated Cu-Kα1 radiation (λ = 1.54051 Å) in Debye-Scherrer geometry. WinXPOW 3.0.2.1 was used for data analysis.

Electrochemistry

Cyclic voltammetry was conducted on a WaveDriver 200 EIS Bipotentiostat with COF-coated carbon paper working electrodes, a platinum wire counter electrode, and a non-aqueous Ag/Ag^+ reference electrode. Anhydrous acetonitrile with 0.1 M tetrabutylammonium hexafluorophosphate was used as electrolyte under Ar atmosphere. Prior to the measurement, the electrochemical cell was purged with argon for 10 min. Reduction onset potentials (E_{onset}) were extracted from the linear fits in the voltammograms according to a previous method.[4] Potentials *vs.* Fc/Fc^+ were converted to absolute energies according to equation S1:[5,6]

$$E_{abs} = -(E_{(vs.\ Fc/Fc+)} + 5.1)\ \text{eV} \qquad \text{(eq. S1)}$$

The position of the valence band (E_{VB}) was estimated from the conduction band (E_{CB}) using the optical band gap $E_{g,opt}$ according to equation S2:

$$E_{VB}\ (\text{eV } vs.\ \text{vac}) = E_{CB}\ (\text{eV } vs.\ \text{vac}) + E_{g,opt}\ (\text{eV}) \qquad \text{(eq. S2)}$$

The absolute energy for water oxidation at pH 7 was calculated according to equation S3:[7]

$$E_{abs} = -(4.5 + 1.23 - 0.059 \cdot \text{pH})\ \text{eV} \qquad \text{(eq. S3)}$$

Electrocatalytic oxygen evolution experiments under aqueous conditions were conducted in sodium phosphate buffer (0.5 M, pH 7). COF samples were measured as coated carbon paper working electrodes, whereas dissolved [Cp*Ir(bpy)Cl]Cl (1.13 mM) was measured against a glassy carbon rotating disk electrode (1200 rpm). In both cases, an RHE electrode was used as reference electrode, and *iR* drop correction was applied.

Electron paramagnetic resonance (EPR) spectroscopy

EPR spectra were measured with a BRUKER EMXnano. Experiments were conducted with degassed silver nitrate solutions (10, 100, or 1000 mM in water). Illumination was conducted with >420 nm similar to photocatalytic OER experiments (*vide infra*). DMPO was added either before or directly after the illumination in the form of a 0.3 M stock solution in order to trap $OH^{\bullet}$ in the form of DMPO-$OH^{\bullet}$. $[Ag^{II}(py)_4]S_2O_8$ was measured as a solid.

Catalytic Activity and Literature Comparison

TONs were calculated according to: TON= $\frac{n(O_2)}{n(metal)}$ TOFs were calculated from TONs through division by the underlying time periods. If stated, literature values were extracted from graphs using WebPlotDigitizer V4.6 by Ankit Rohatgi.

Photocatalytic Oxygen Evolution - Screening

If not stated otherwise, 5.0 mg COF were suspended in the respective aqueous reaction medium (5 mL) and sonicated for at least 10 minutes. The resulting suspension was transferred to a custom-made flow reactor (Figure S 43) and the sacrificial electron acceptor was added. The reactor was closed and the reaction mixture was degassed in the dark with an argon flow of 40-60 NmL min^{-1} while stirring at 400 rpm. Once the system approached the baseline oxygen content, the flow was reduced to 20 NmL min^{-1} and the temperature of the water-jacketed reactor was kept at 25 °C using a JULABO FP50-ME thermostat. After adjusting the pressure to 1.10 – 1.25 bar, the baseline was measured for 30 minutes before starting the illumination from above through a quartz glass blind flange. The oxygen evolution rate was determined every three seconds using a PreSens flow-through cell with an integrated PSt-9 sensor spot connected to a Fibox 4 trace oxygen meter. The readout in ppm was baseline-corrected and subsequently converted to µmol h^{-1} by applying a factor of 0.0749 µmol h^{-1} ppm^{-1} (Figure S 43). To prevent uncontrolled heating of the oxygen sensor during illumination, it was covered in wet paper towels together with the Pt100 temperature sensor. If not stated or depicted otherwise, illumination was performed for 90 minutes. In between measurements, the reactor was cleaned with *aqua regia*, piranha solution, and copious amounts of water. If stated, the flow reactor was connected to a SHIMADZU GC-2030 with MS and BID detection in addition to the online PSt-9 detector. In these cases, the pressure is kept stable at 1.15 bar using a pressure controller, and a flow of 5 NmL min^{-1} helium is applied to increase the sensitivity of the oxygen detection (conversion factor 0.01338 µmol h^{-1} ppm^{-1}). The difference in conversion from mass flow readout to reaction rates compared to the other inert gas choice lies on the gas factor of the mass flow controller (MFC) manufacturer calibration. Illumination was achieved with an AAA class Newport 94023A solar simulator. If explicitly stated, the flow reactor was replaced with a bulk photoreactor that also allows for top-down illumination and measurement under flow conditions.

For stability experiments and postcatalytic analysis, the amount of COF and reaction media were upscaled while mimicking catalysis conditions in a round-bottom flask. Ir@TAPB-BPY COF (39.8 mg) was sonicated in water (40 mL) for 10 minutes and subsequently degassed by inert gas bubbling for 10 minutes. Solid $AgNO_3$ (68.4 mg, 0.4 mmol) was added, and the resulting suspension was stirred under continuous inert gas bubbling during illumination for 90 minutes with a 300 W Xe lamp (>420 nm). The solid was filtered off, washed with water, acetone, and ethanol, prior to supercritical CO_2 drying and subsequent heating *in vacuo* (12 h, 120 °C). Ir@TAPB-BPY COF was retrieved as an ocre solid (39.1 mg, 98%).

Chemical Oxygen Evolution

If not stated otherwise, 5.0 mg COF were suspended in 4.6 mL 0.1 M HNO_3 and sonicated for 5 minutes. The resulting suspension was transferred to a custom-made flow reactor (Figure 3) and degassed in the dark with an argon flow of 40-60 NmL min^{-1} while stirring at 400 rpm. Once the system approached the baseline oxygen content, the flow was reduced to 20 NmL min^{-1} and the temperature of the water-jacketed reactor was kept at 25 °C. After adjusting the pressure to 1.10 – 1.25 bar, the baseline was measured for 30 minutes before injecting a blank (0.1 M HNO_3, 0.2 mL) through a septum injector nut. After another 30 minutes, 0.2 mL of a CAN stock solution (1.95 M, in 0.1 M HNO_3) were injected, yielding a final CAN concentration of 78 mM. The oxygen evolution rate was determined every three seconds using a PreSens flow-through cell with an integrated PSt-6 or PSt-9 sensor spot connected to a Fibox 4 trace oxygen meter. The readout in ppm was converted to µmol h^{-1} by applying a factor of 0.0749 µmol h^{-1} ppm^{-1}. For recycling and filtration experiments, either the filtrate or the residual COF were subjected to identical reaction conditions. In the latter case, the COF was washed with 0.1 M HNO_3, water, and acetone after the first catalytic experiment.

For reactions with a final CAN concentration of 10 mM, the blank and the CAN stock injection were done with a gastight HAMILTON® syringe (50 µL; final volume still 5 mL) in order to reduce the oxygen leakage.

For stability experiments, the amount of COF and reaction media were upscaled while mimicking catalysis conditions in round-bottom flasks. 50 mg COF were sonicated in aq. HNO_3 (50 mL, pH 1) for 10 minutes, and subsequently degassed by inert gas bubbling for 1 hour. Solid CAN was added to reach the stated final concentration, and the resulting suspensions were stirred under continuous inert gas bubbling for 90 minutes. The solid was filtered off, washed with HNO_3 (pH 1), water, DMF, acetone, and MeOH, prior to supercritical CO_2 drying and subsequent heating *in vacuo* (12 h, 120 °C).

Methanol Oxidation

Methanol oxidation was performed by dispersing 3 mg of catalyst in 3 mL of a 10 mM $AgNO_3$ solution in D_2O and sonicated for 10 min. Then, 0.1 mL of methanol was added to the dispersion and transferred to a custom-made flow reactor (Figure S 43). After 10 h of irradiation with a solar simulator, 0.5 mL of the reaction solution was taken with the help of a syringe and passed through a 0.22 µm hydrophilic PTFE syringe filter. Finally, 0.1 mL of DMSO-d_6 was added to the D_2O/methanol mixture for subsequent ^{1}H-NMR analysis.

Computational Chemistry

The ionisation potential (IP) and electron affinity (EA) of the polymers in water were predicted by ΔDFT calculations following a previously developed approach.[8,9] In this approach the COF is described as a cluster model (see Figure S 73) embedded in a continuum dielectric with the dielectric permittivity of the major component of the reaction mixture, here water (ε_r 80.1) or acetonitrile (ε_r 37.5). The use of a continuum solvation model allows for the description of the dielectric screening of charges in the low dielectric COF/polymer by the typically higher dielectric permittivity mixture of water sacrificial electron donor/acceptor mixture. Moreover, when using a dielectric permittivity value of 2 instead, roughly the value expected for an organic material, this approach reproduces IP and EA values measured experimentally[9,10] for conjugated polymers by photoelectron spectroscopy, in which the polymer particles/film is measured in vacuum and there is no solvent/reaction mixture present.

All predicted potentials were converted from the vacuum scale to the standard hydrogen electrode (SHE) scale by subtracting 4.44 V, the absolute value of the standard hydrogen electrode potential, of the vacuum scale value.

The free energy landscape of a step wise water oxidation mechanism for a cluster model of the Ir@TAPB-BPY COF and Co@TAPB-BPY COF was calculated using an approach adapted from a method originally developed by Norskov and co-workers for heterogeneous electrocatalysts.[11–13] This approach allows one to predict the overpotential required to drive water oxidation relative to the potential of the four-hole water oxidation reaction by the difference between the potential for the elementary redox step with the largest uphill free energy change and that of the four-hole water oxidation potential. In the calculations we equate the free energy of a proton and an electron with that of ½ H_2, the so-called computational hydrogen electrode approximation. The entropic contribution to the free energy was calculated by calculating the harmonic frequencies of all relevant cluster models, as well as molecular hydrogen and water. Because of the cost of the frequency calculations the cluster model used in these calculations is smaller than that used when calculating IP and EA (Figure S 73). However, a calculation of only the enthalpic contribution to the free energy for the model in Figure S 73 and the larger model in Figure S 82 suggest that the effect of using such smaller models is small.

All DFT calculations used the B3LYP density functional[14–17] in combination with the DZP[18] (def2-SVP[19] in combination with a relativistic ECP[20] for Ir) basis-set and were performed using Turbomole 7.5.[21,22] Solvation effects in the DFT calculations were described using the COSMO[23] implicit continuum solvation model and the water/acetonitrile dielectric permittivity value discussed above.

Structural data relevant to computational chemistry is provided as supporting information in the form of a .zip file.

S2 - Syntheses

$[Cp^*IrCl_2]_2$,[24] $[Cp^*Ir(bpy)Cl]Cl$,[25] TTI-COF,[26] Co@TTI-COF,[27] Co@TAPB-BPY COF,[28] Co@TAPT-BPY COF,[29] $RuO_2@WO_3$,[30] and $[Ag^{II}(py)_4]S_2O_8$[31] were synthesized according to literature procedures.

<u>Synthesis of iridium-loaded bipyridine linker **2**</u>

Following a literature procedure[32] $[Cp^*IrCl_2]_2$ (199 mg, 0.25 mmol) and 2,2'-bipyridiyl-5,5'-dialdehyde (108 mg, 0.5 mmol) were dissolved in DCM (6 mL) and stirred at rt for 42 h. The resulting orange solution was filtered through a 0.45 µm PTFE syringe filter and evaporated using a stream of nitrogen, yielding **2** as an orange solid (284.8 mg, 93%).

^{1}H NMR (400 MHz, Chloroform-*d*) δ 10.28 (s, 2H), 9.44 (d, *J* = 8.2 Hz, 2H), 9.25 (s, 2H), 8.68 (d, *J* = 8.1 Hz, 2H), 1.77 (s, 15H) ppm. ^{1}H NMR (400 MHz, Methanol-d_4, hydrate formation) δ 9.07 (d, *J* = 6.9 Hz, 2H), 8.60 (d, *J* = 9.9 Hz, 2H), 8.30 (d, *J* = 8.4 Hz, 2H), 5.81 (s, 2H), 1.71 (s, 15H). ^{13}C NMR (101 MHz, Methanol-d_4, hydrate formation) δ 156.0, 151.0, 144.0, 139.6, 124.8, 95.5, 91.0, 8.6 ppm. MS (ESI+): m/z calc. for $C_{22}H_{23}ClIrN_2O_2^+$ (M-Cl^-): 575.10718; found 575.10835. ICP: 33.49 wt% Ir; calc. 31.479 wt%.

<u>Synthesis of TAPB-BPDA COF</u>

A Biotage® 5 mL microwave vial was charged with TAPB (14.8 mg, 0.039 mmol, 2.0 eq.) and 4,4'-biphenyldicarboxaldehyde (BPDA, 12.9 mg, 0.060 mmol, 3.0 eq.). The vial was temporarily sealed with a rubber septum and flushed three times *via* vacuum/argon cycles. Mesitylene (1.33 mL) and 1,4-dioxane (0.66 mL) were added, and the reactants were suspended *via* sonication for 5 minutes. The suspension was degassed *via* three vacuum/argon cycles. Aqueous acetic acid (100 µL, 6M) was added, the vial was sealed with a crimp cap and heated to 130 °C for 3 d. After cooling to room temperature, the combined solids of two parallel reactions were filtered off and washed with $CHCl_3$ (3 x 15 mL), THF (3 x 10 mL), and acetone (3 x 10 mL). Drying in high vacuum at 120 °C for 12 h yielded TAPB-BPDA COF (35.2 mg, 73%) as an orange-brown solid. Elemental analysis calc. (%) for $C_{90}H_{60}N_6$: C 88.21, H 4.93, N 6.86; found: C 86.04, H 4.91, N 6.44.

<u>Synthesis of TAPB-BPY COF</u>

A Biotage® 20 mL microwave vial was charged with TAPB (92.3 mg, 0.252 mmol, 2.0 eq.) and 2,2'-bipyridyl-5,5'-dialdehyde (81.9 mg, 0.378 mmol, 3.0 eq.). The vial was temporarily sealed with a rubber septum and flushed three times *via* vacuum/argon cycles. Mesitylene (5.1 mL) and 1,4-dioxane (0.9 mL) were added, and the reactants were suspended *via* sonication for 5 minutes. The suspension was degassed *via* three vacuum/argon cycles. Aqueous acetic acid (600 µL, 6M) was added, the vial was sealed with a crimp cap and heated to 120 °C for 3 d. After cooling to

room temperature, the solid was filtered off and washed with DMF (50 mL), THF (50 mL), acetone (50 mL), and MeOH (50 mL). Soxhlet extraction with MeOH overnight followed by supercritical CO_2 drying yielded TAPB-BPY COF (118 mg, 76%) as an ocre powder. Elemental analysis calc. (%) for $C_{84}H_{54}N_{12}$: C 81.93, H 4.42, N 13.65; found: C 79.38, H 4.59, N 12.72.

TAPT-BPY COF was synthesized accordingly.

Synthesis of Ir@TAPB-BPY COF

Ir@TAPB-BPY COF was synthesized according to the procedure described for TAPB-BPY COF but with substitution of bipyridyl-5,5'-dialdehyde by the desired amount of iridium-loaded linker **2**.

Postsynthetic loading of TAPB-BPY COF with Iridium

A Biotage® 20 mL microwave vial was charged with TAPB-BPY COF (33.5 mg, 0.082 mmol bpy, 1.0 eq.) and $[Cp^*IrCl_2]_2$ (33.7 mg, 0.042 mmol, 0.5 eq.). The vial was temporarily sealed with a rubber septum and flushed three times *via* vacuum/argon cycles. Degassed methanol (5 mL) was added, and the suspension was stirred for two hours. The solid was filtered off and washed with MeOH (5 mL), water (5 mL), DMF (15 mL), EtOH (15 mL), THF (15 mL), acetone (50 mL), and MeOH (5 mL). Soxhlet extraction with MeOH overnight followed by supercritical CO_2 drying yielded Ir@TAPB-BPY COF (46.5 mg, 98 %) as an orange powder.

The attempted loading of TAPB-BPDA COF was performed analogously.

S3 - COF Characterization

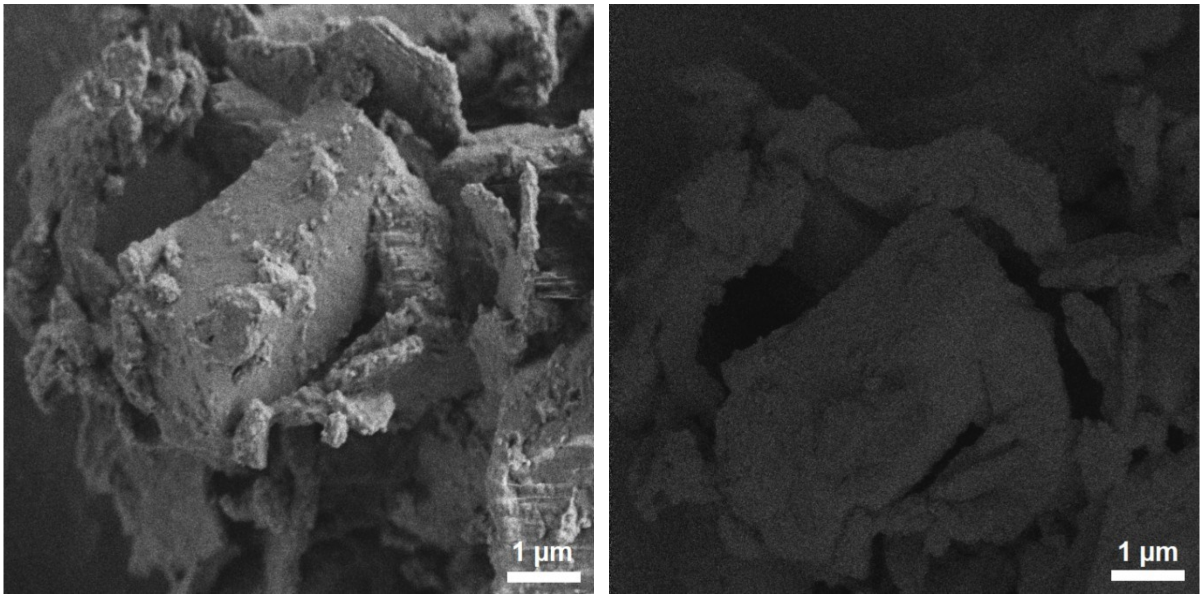

Figure S 1: SEM images of TAPB-BPY COF with secondary electron detection (left) and energy selective backscattered electron detection (right).

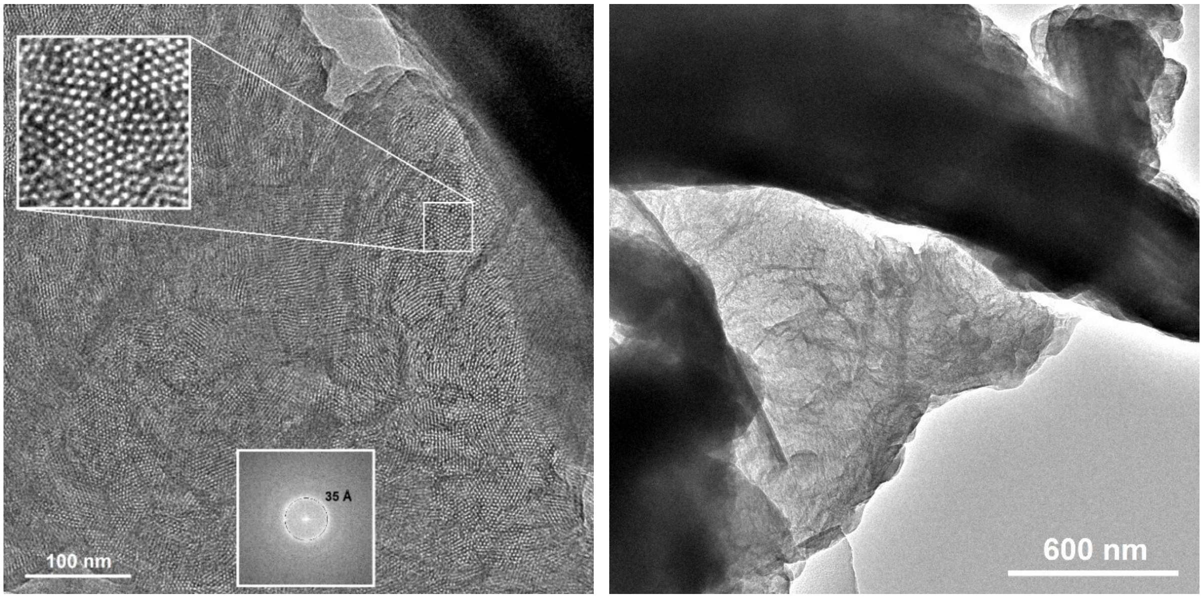

Figure S 2: TEM images of TAPB-BPY COF. Magnified area highlights hexagonal arrangement of the pore. Inset shows FFT.

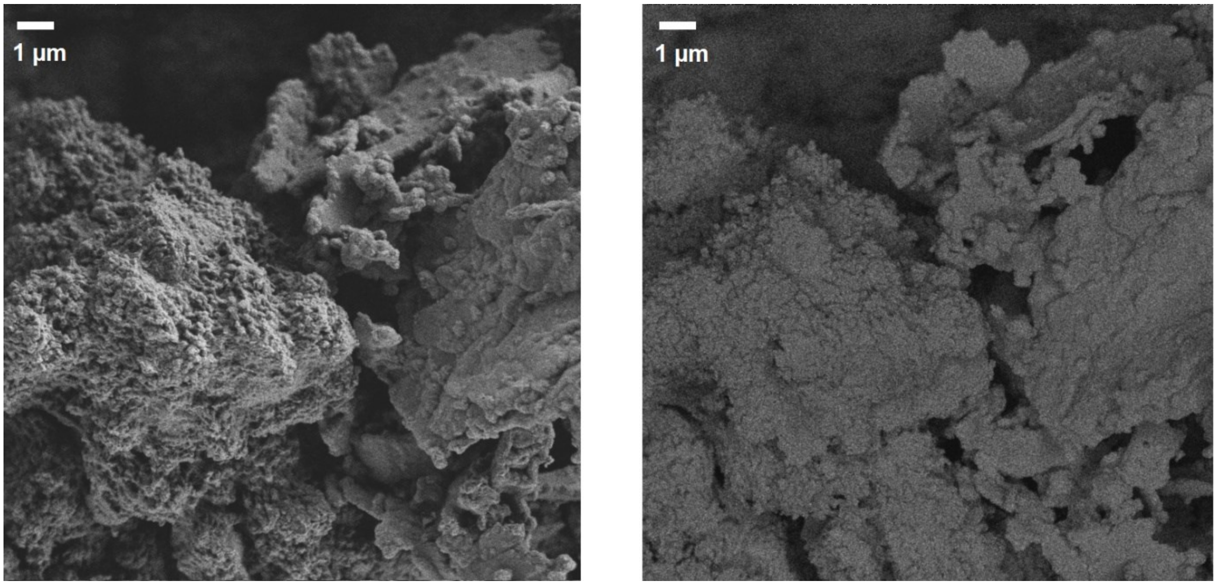

Figure S 3: SEM imaging of TAPB-COF after postsynthetic loading with $[Cp^*IrCl]_2$ with secondary electron detection (left) and energy selective backscattered electron detection (right).

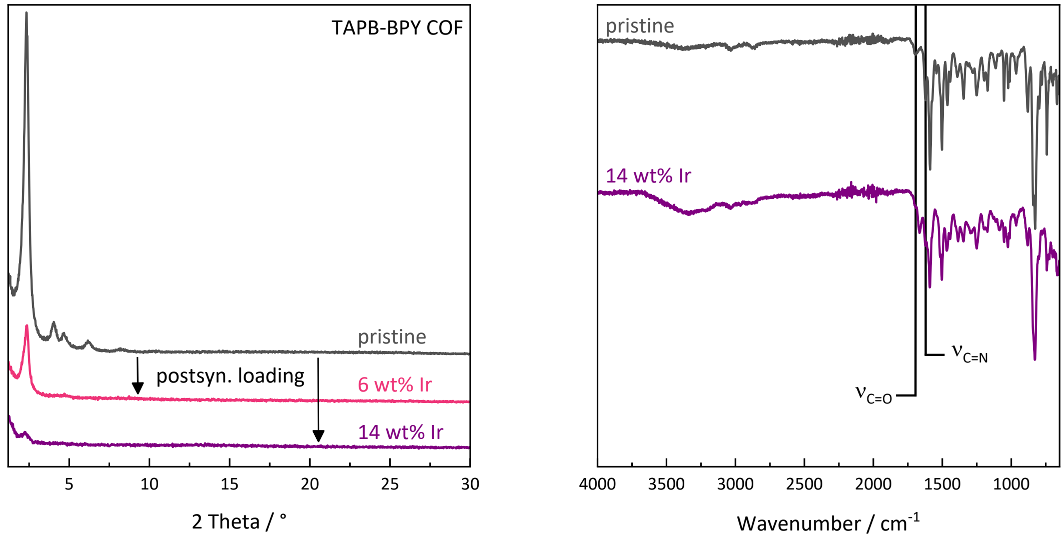

Figure S 4: XRPD patterns (left) and FTIR spectra (right) for TAPB-COF before and after postsynthetic loading with varying amounts of [Cp*IrCl]. Annotations refer to the Ir content after loading as measured by ICP-OES.

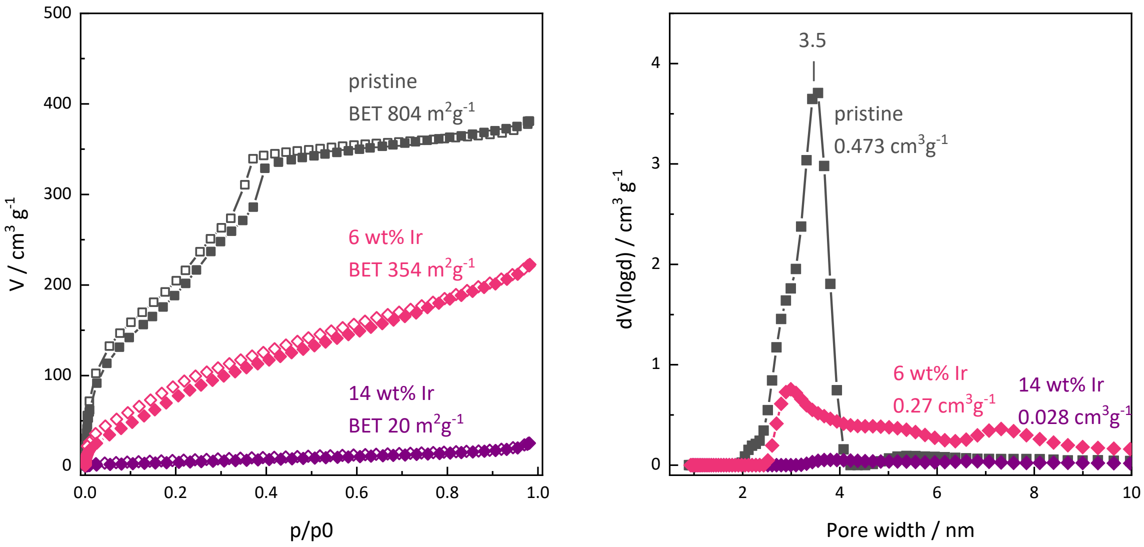

Figure S 5: Argon sorption isotherm at 87 K (left) and pore size distribution (right) for TAPB-COF before and after postsynthetic loading with varying amounts of $[Cp*IrCl_2]_2$. Filled and open symbols represent the adsorption and the desorption branches, respectively. The pore size distribution was obtained from a QSDFT kernel for cylindrical pores (adsorption branch).

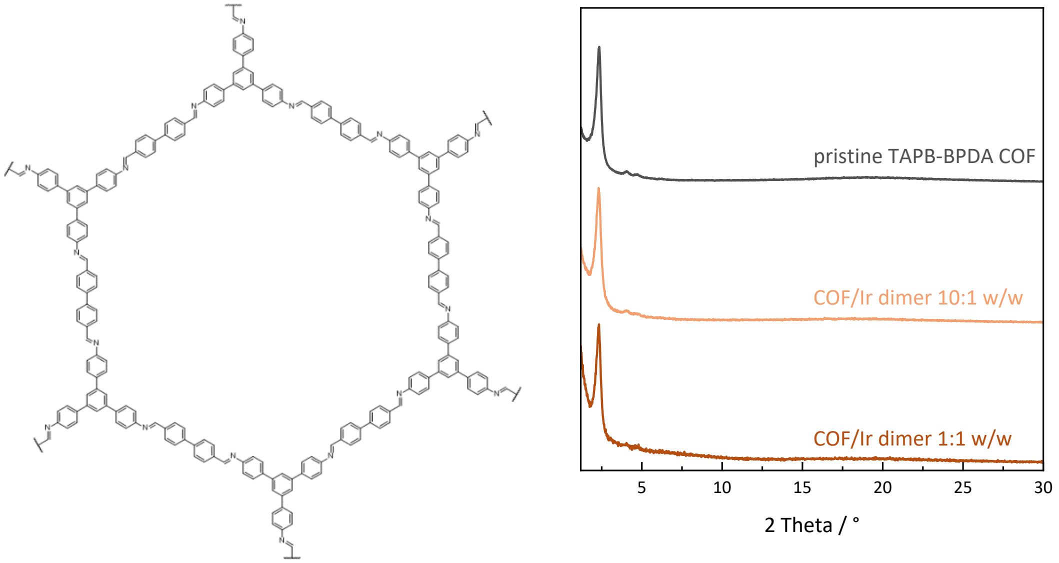

Figure S 6: Molecular structure of TAPB-BPDA COF (left) and XRPD patterns for the attempted loading of TAPB-BPDA COF with the given amounts of $[Cp*IrCl_2]_2$ **1** (right). ICP analysis shows no sign of Ir in either case.

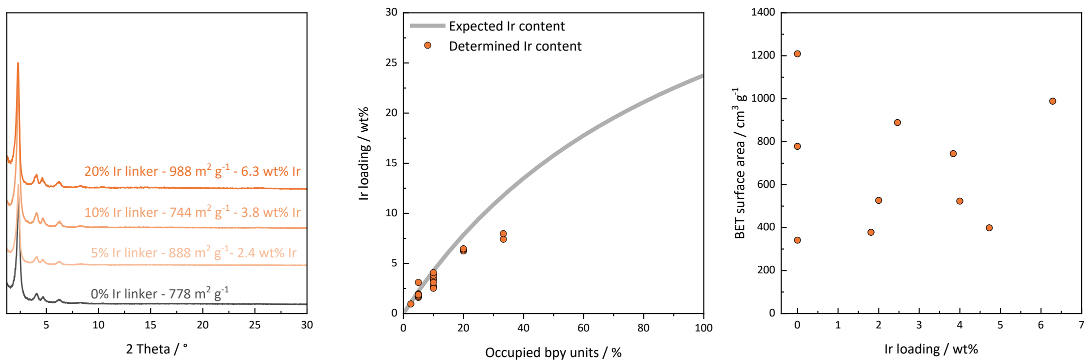

Figure S 7: XRPD patterns for a series of TAPB-COF with varying amounts of iridium-loaded linker **2** (left). BET surface areas and Ir content (ICP-OES) given as annotations. Comparison of expected and determined iridium contents (middle) and BET surface area and Ir loading (right). Each data point represents one individual synthesis. Diverging BET values for separate batches with similar Ir loading can be traced back to a faulty $scCO_2$ drying procedure.

The expected iridium content w_{Ir} was calculated from the percentage x of employed iridium-loaded linker **2** according to:

$$w_{Ir}(\mathrm{x}) = \frac{3 \cdot \mathrm{x} \cdot \mathrm{M(Ir)}}{\mathrm{M(COF)} + 3 \cdot \mathrm{x} \cdot \left(\mathrm{M(Ir)} + \mathrm{M(Cp^*)} + 2\,\mathrm{M(Cl)}\right)} \cdot 100\ \mathrm{wt\%}$$

Whereas M(COF) is the molecular weight of an ideal stoichiometric model of the COF containing three bipyridine units connected to two TAPB linkers *via* imine bonds.

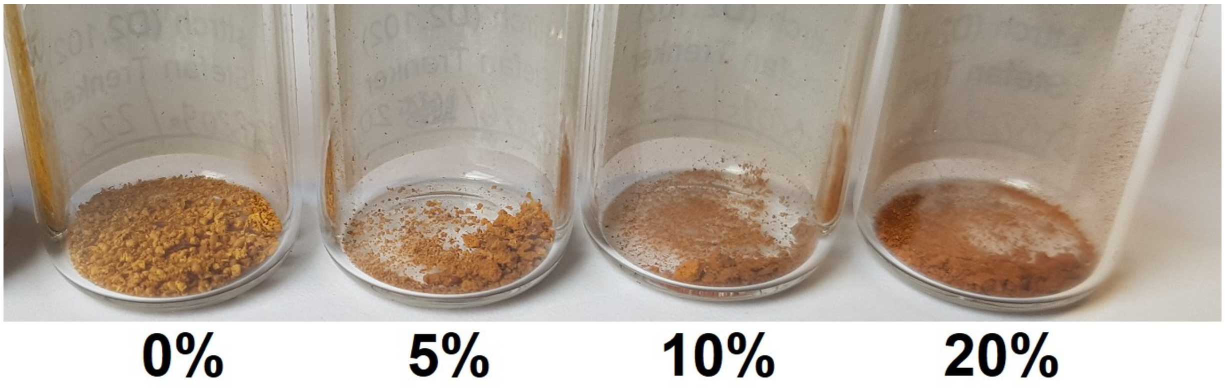

Figure S 8: Photographic image of TAPB-BPY COF constructed from the specified amount of iridium-loaded linker **2**.

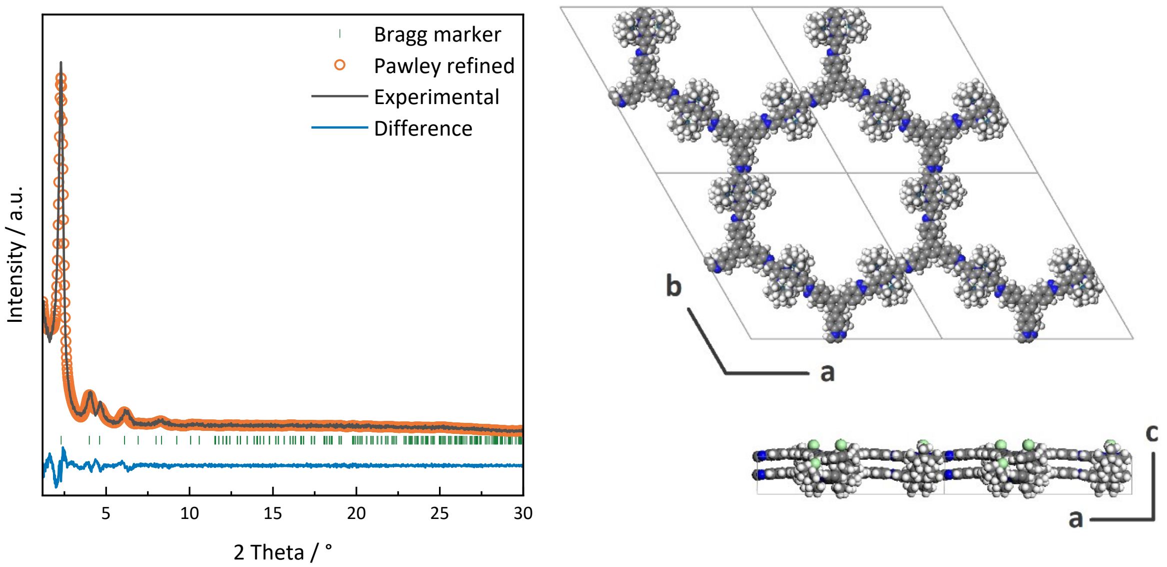

Figure S 9: Left: XRPD pattern and Pawley refinement for Ir@TAPB-BPY COF synthesized from Ir-loaded linker **2**. Right: Respective structural model. Atom color coding: H – white; C – gray; N – blue; Cl – green; Ir – dark blue. For Pawley refinement, a site occupancy of 0.1 was assumed for all atoms in the Cp*IrCl fragment.

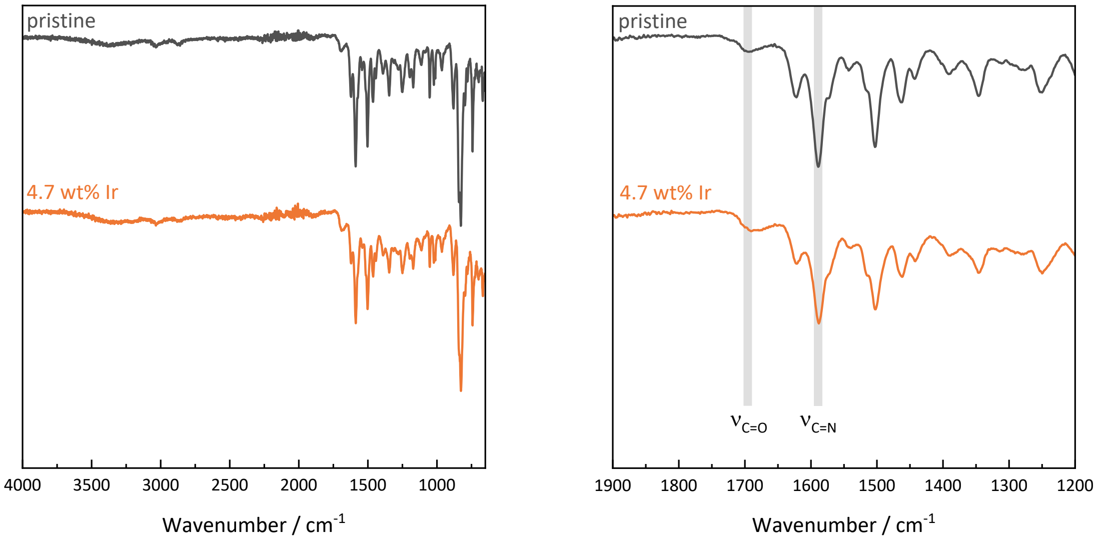

Figure S 10: FTIR spectra of pristine and iridium-loaded TAPB-BPY COF. The stretching vibrations at 1693 cm^{-1} indicate residual aldehyde functionalities in both cases.

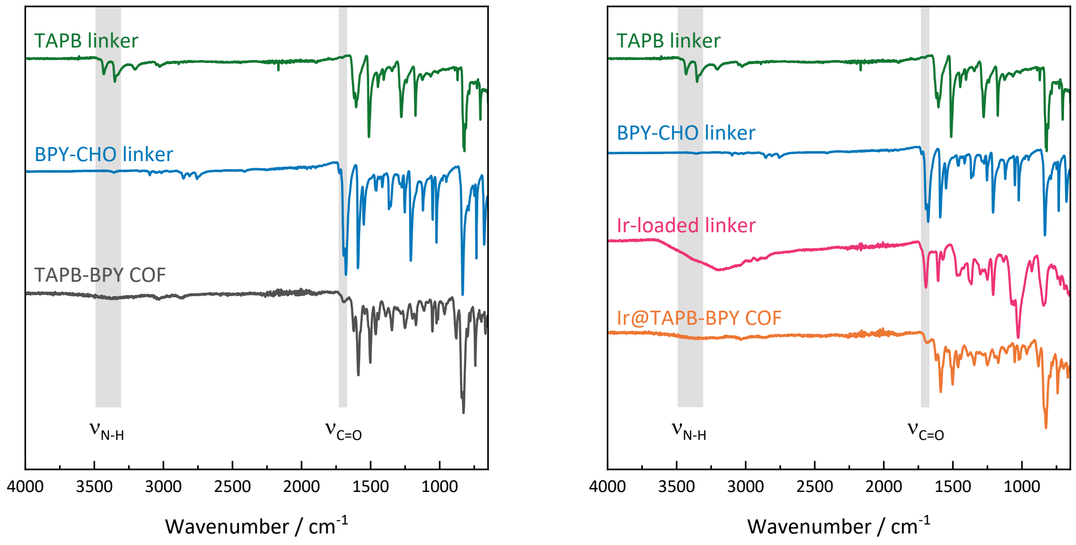

Figure S 11: FTIR spectra of pristine TAPB-BPY COF (left) and Ir@TAPB-BPY COF (right) and their respective building blocks.

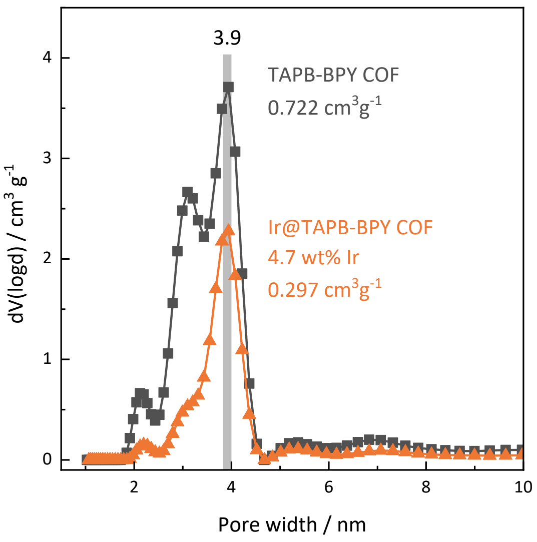

Figure S 12: Pore size distribution and pore volumes for TAPB-BPY COF and Ir@TAPB-BPY COF constructed from iridium-loaded linker **2**. The PSD was calculated from the adsorption branch of the nitrogen sorption isotherms with a QSDFT kernel assuming cylindrical pores.

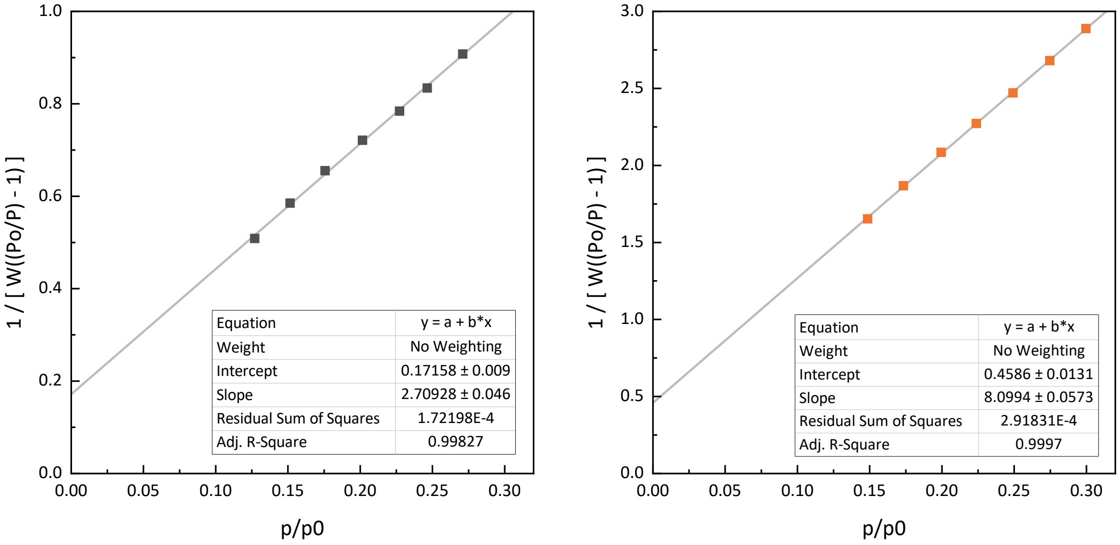

Figure S 13: BET plots for TAPB-BPY COF (left) and Ir@TAPB-BPY COF constructed from iridium-loaded linker **2** (right).

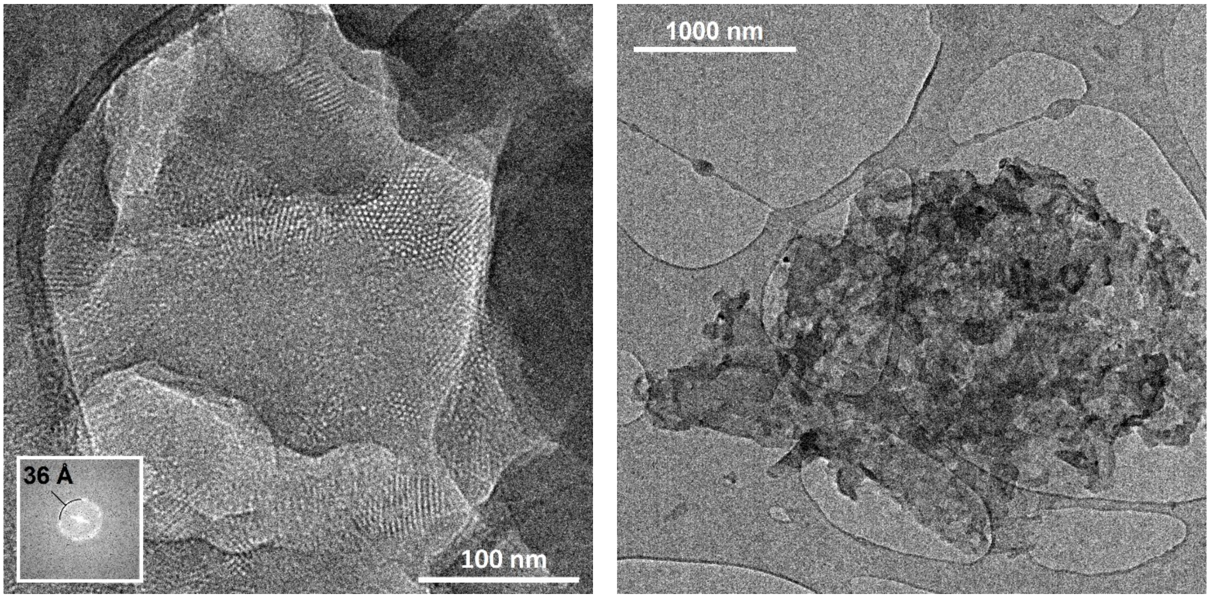

Figure S 14: TEM images of Ir@TAPB-BPY COF constructed from iridium-loaded linker **2**. Inset shows FFT.

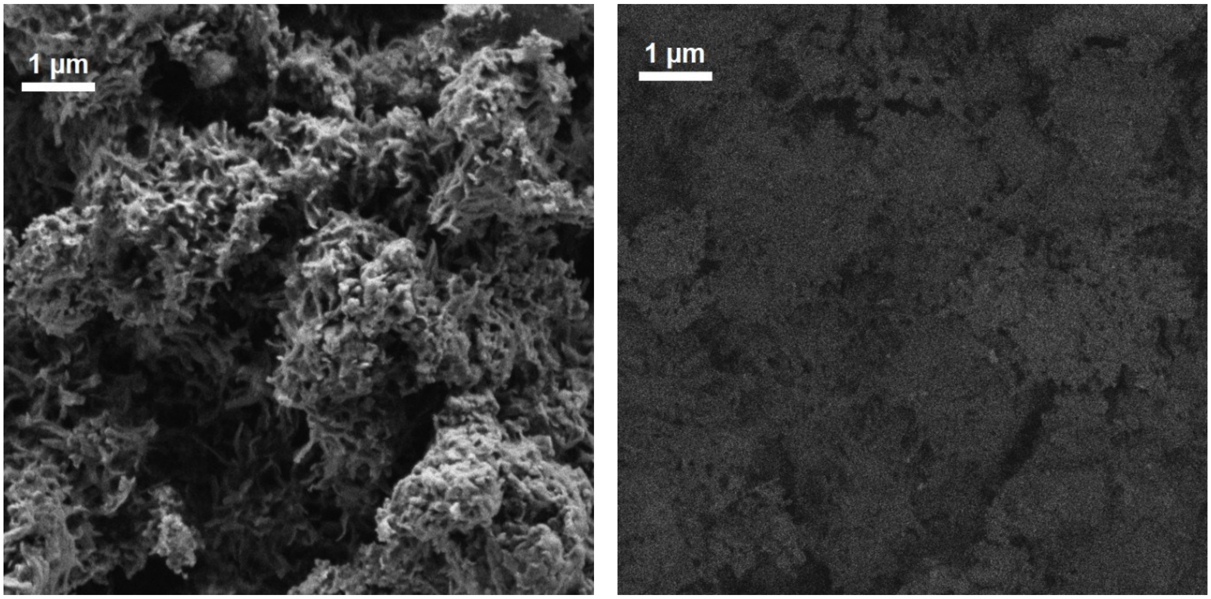

Figure S 15: SEM images of Ir@TAPB-BPY COF constructed from iridium-loaded linker **2** with secondary electron detection (left) and energy selective backscattered electron detection (right).

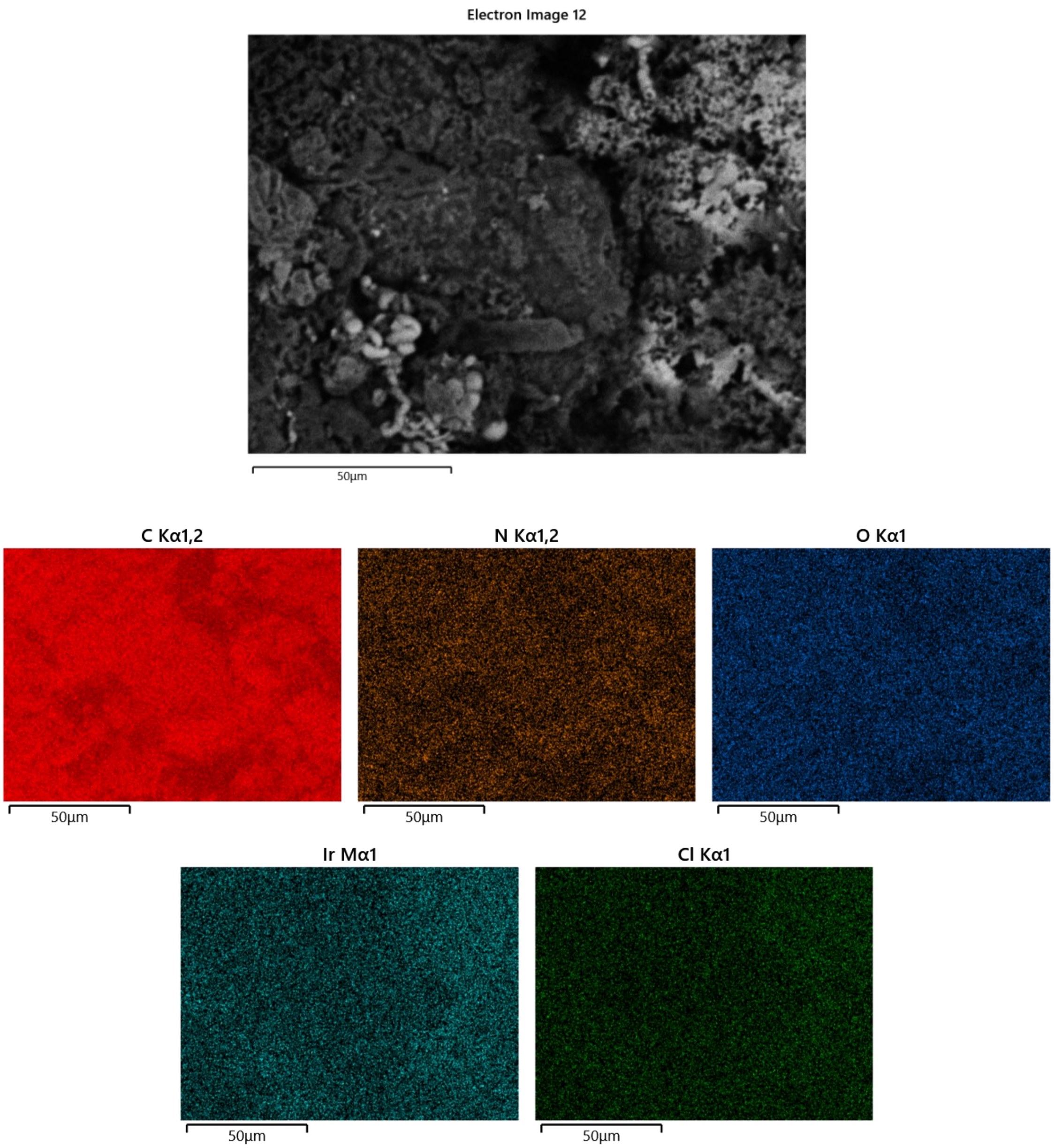

Figure S 16: SEM elemental mapping of Ir@TAPB-BPY COF constructed from iridium-loaded linker **2**.

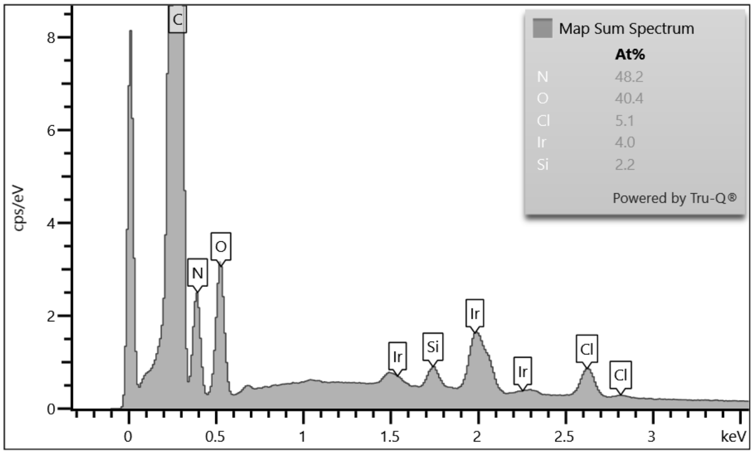

Figure S 17: Summed-up EDX spectra for the elemental mapping of Ir@TAPB-BPY COF.

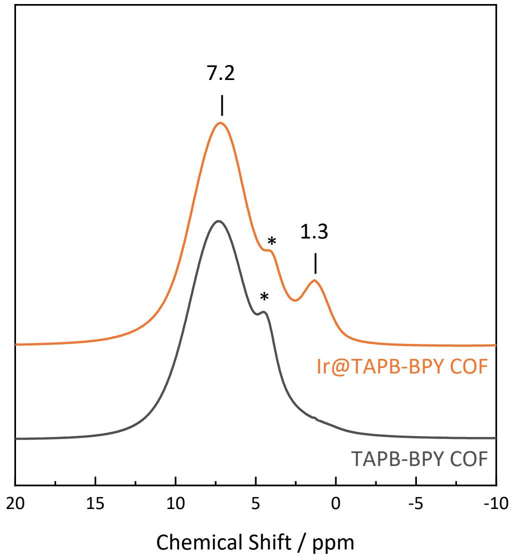

Figure S 18: ^{1}H-ssNMR spectra for TAPB-BPY COF and Ir@TAPB-BPY COF constructed from iridium-loaded linker **2**. Asterisks mark residual water signal.

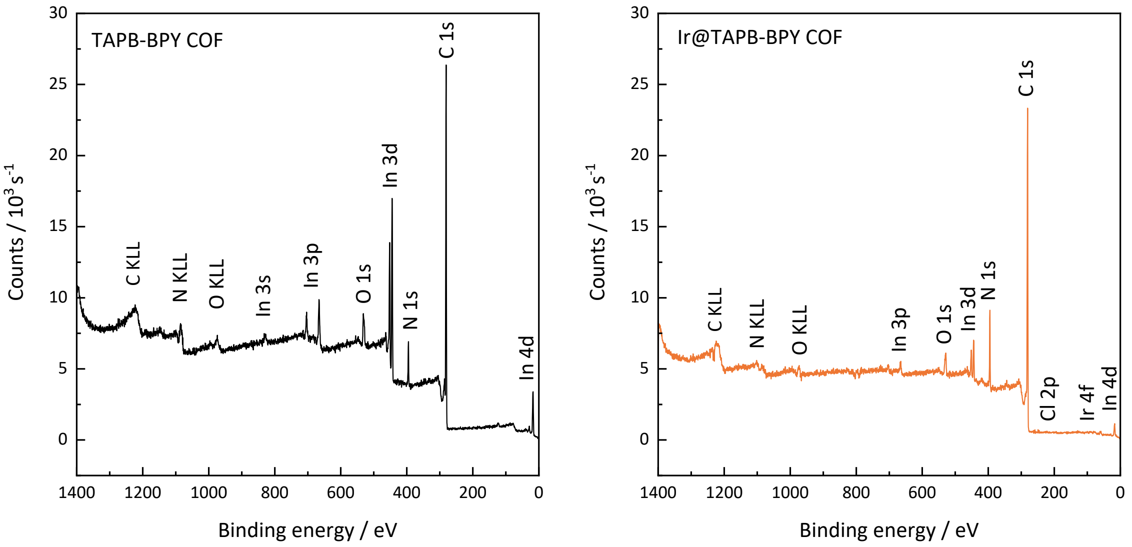

Figure S 19: Survey X-ray photoelectron spectra of TAPB-BPY COF (left) and Ir@TAPB-BPY COF (right).

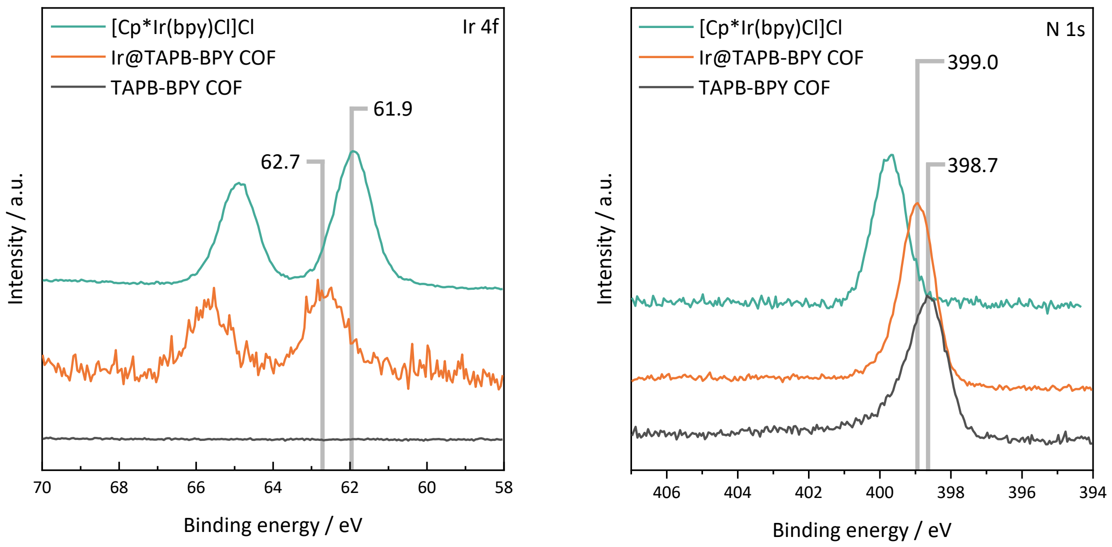

Figure S 20: Ir 4f (left) and N 1s (right) XPS signals for Ir@TAPB-BPY COF, TAPB-BPY COF, and molecular Cp*Ir(bpy)Cl.

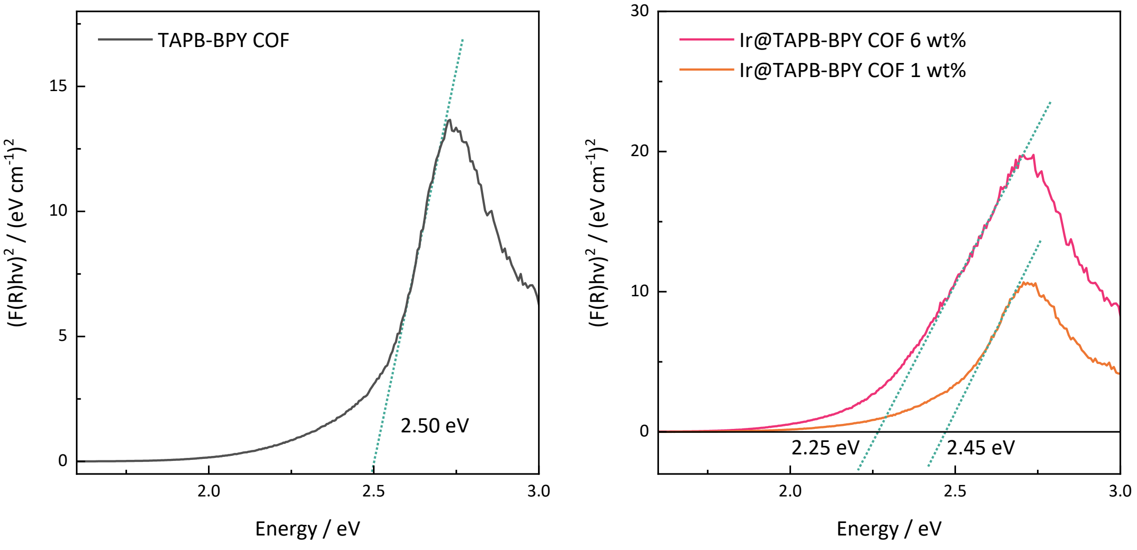

Figure S 21: Tauc plot for TAB-BPY COF (left) and Ir@TAPB-BPY COF with varying Ir content (right). Annotations indicate the optical band gap.

S4 - Chemical Water Oxidation

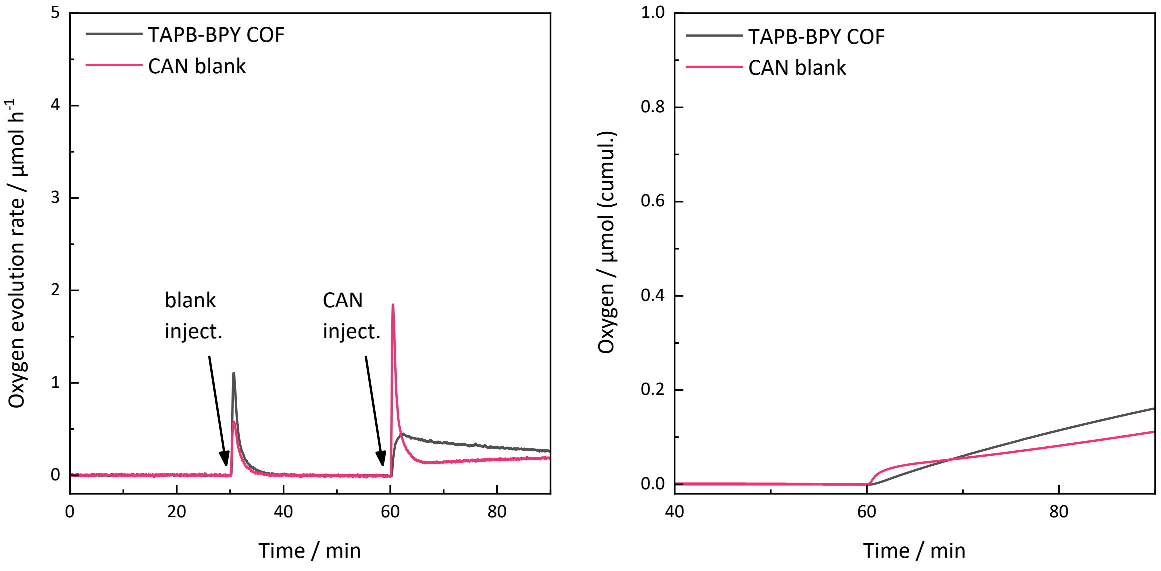

Figure S 22: Blank experiments for the chemical water oxidation with CAN. Reaction condition: 5 mg TAPB-BPY COF (if stated), 78 mM CAN (final conc.) in 5 mL HNO_3 (pH 1).

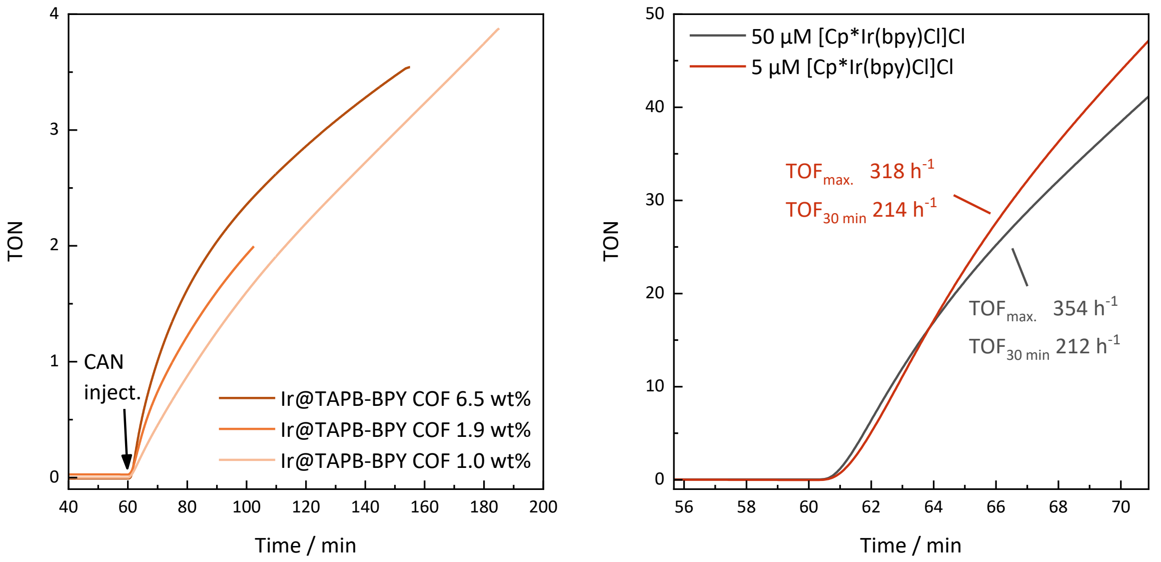

Figure S 23: TON plot for chemical water oxidation experiments with Ir@TAPB-BPY COFs of varying iridium content as annotated (a). TON plot for chemical water oxidation experiments with molecular [Cp*Ir(bpy)Cl]Cl (b). Reaction conditions: 78 mM CAN in 5 mL HNO_3 (pH 1). WOC concentration as annotated.

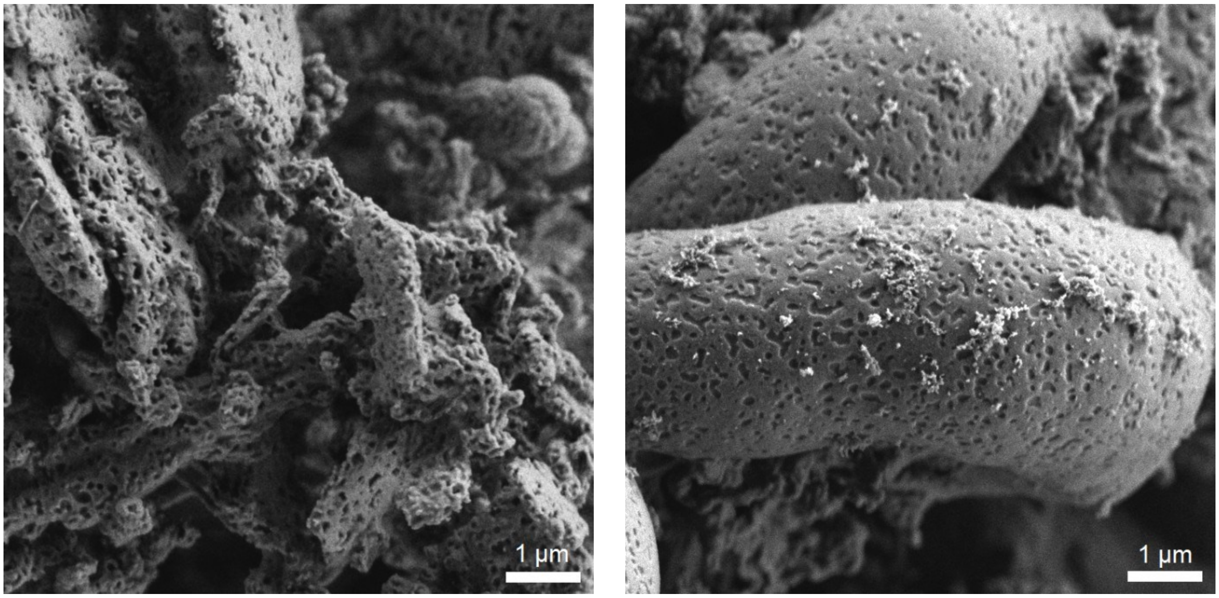

Figure S 24: SEM images of Ir@TAPB-BPY COF before (left) and after (right) water oxidation catalysis experiments with CAN (78 mM).

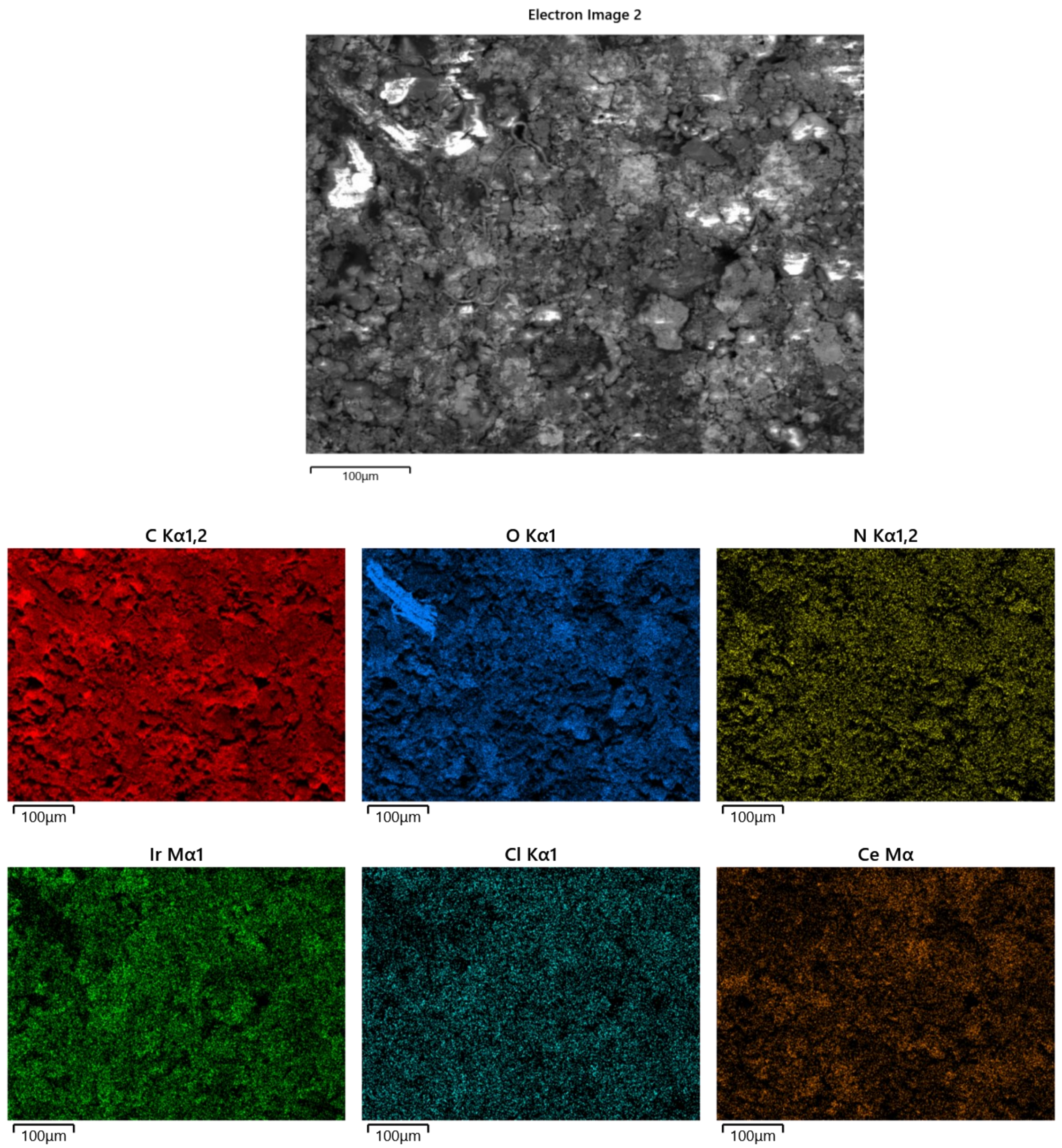

Figure S 25: SEM elemental mapping of Ir@TAPB-BPY COF after water oxidation catalysis experiments with CAN (78 mM).

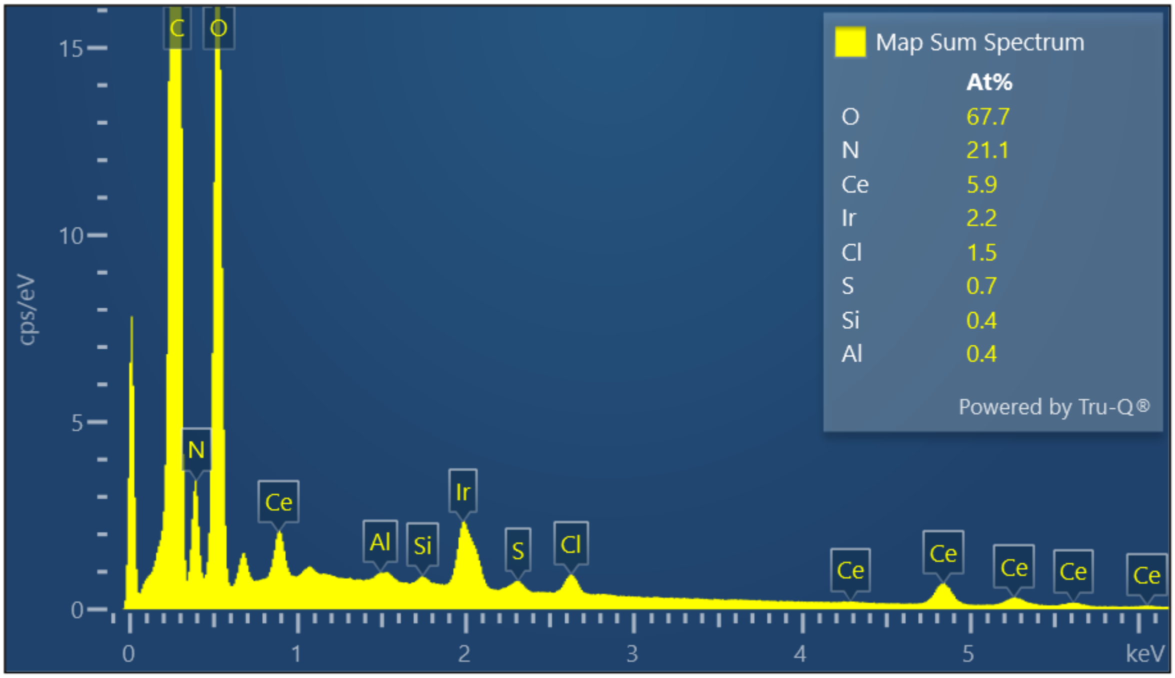

Figure S 26: Summed-up EDX spectra for the elemental mapping of Ir@TAPB-BPY COF after chemical water oxidation catalysis with CAN (78 mM).

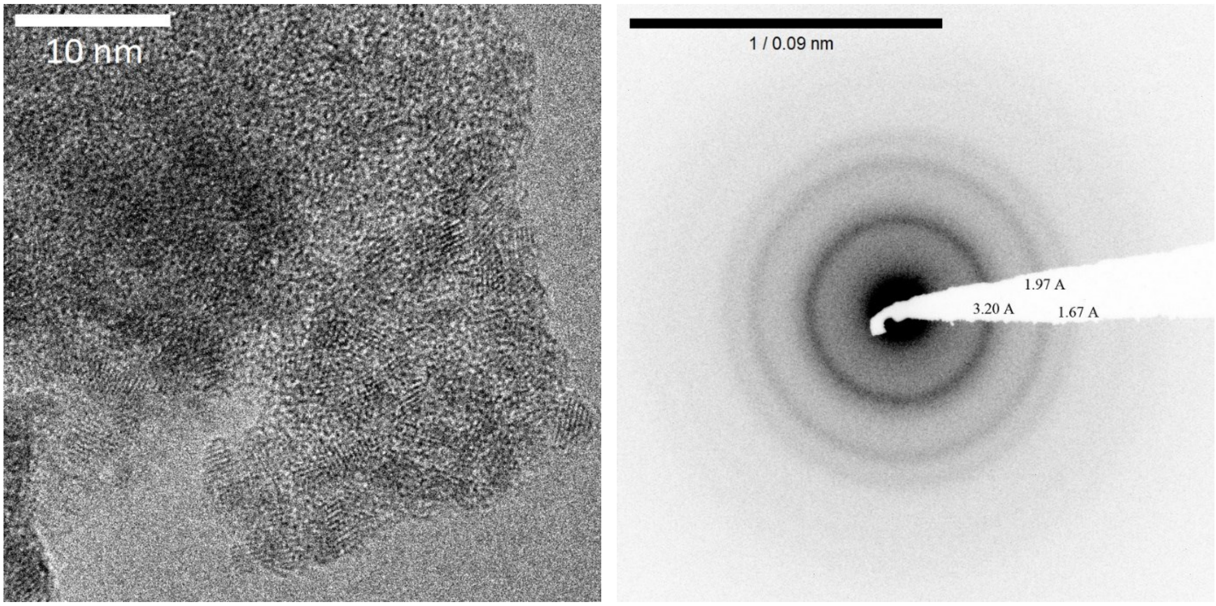

Figure S 27: TEM image (left) and selected area diffraction pattern (right) of Ir@TAPB-BPY COF after oxygen evolution with 78 mM CAN.

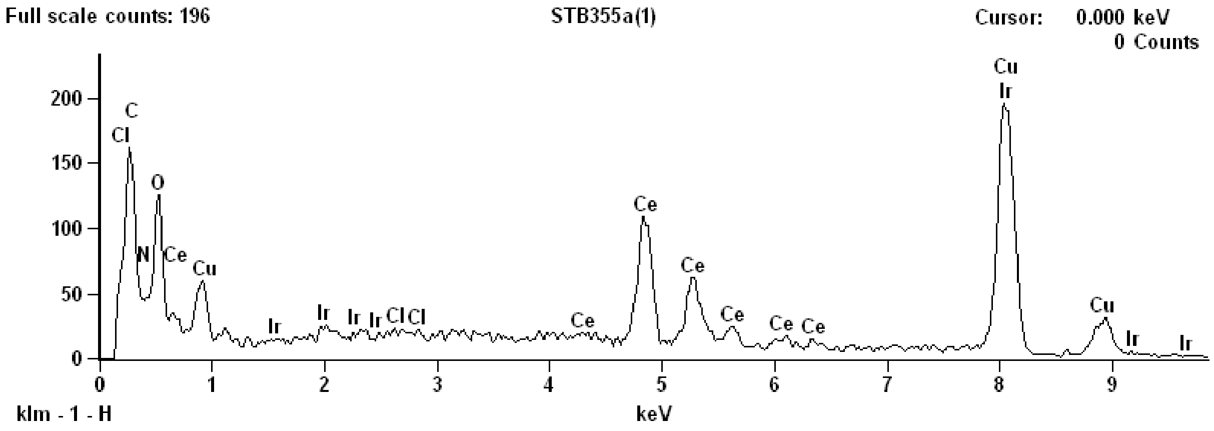

Figure S 28: EDX data for TEM-examined Ir@TAPB-BPY COF after oxygen evolution with 78 mM CAN (Figure S 27). The Ce/Ir/Cl ratio (at%) is 93.6/3.9/2.5.

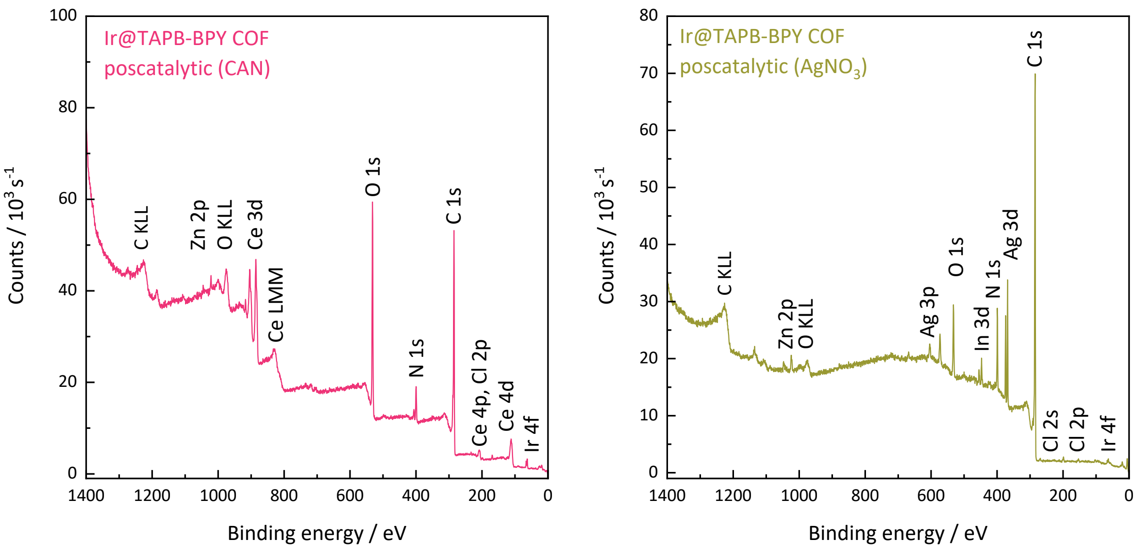

Figure S 29: Survey X-ray photoelectron spectra of Ir@TAPB-BPY COF after oxygen evolution experiments with CAN (78 mM, left) and $AgNO_3$ (10 mM, right). Upscaled reaction conditions were used to retrieve sufficient sample for these analyses – see section S1.

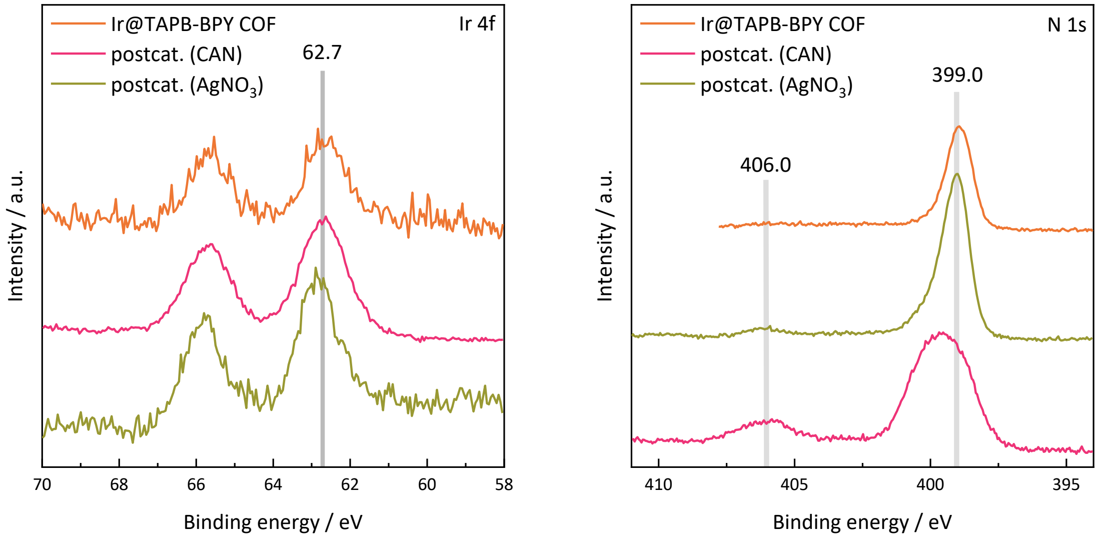

Figure S 30: XPS spectra for Ir@TAPB-BPY COF after oxygen evolution experiments with $AgNO_3$ (10 mM) and CAN (78 mM) compared to pristine Ir@TAPB-BPY COF in the Ir 4f (left) and N 1s (right) ranges. The new signal at 406 eV and the broadening of the peak around 399 eV for the CAN-sample are ascribed to residual nitrate (counter-)ions and protonation and/or oxidation of imines, respectively.[33–36] Upscaled reaction conditions were used to retrieve sufficient sample for these analyses – see section S1.

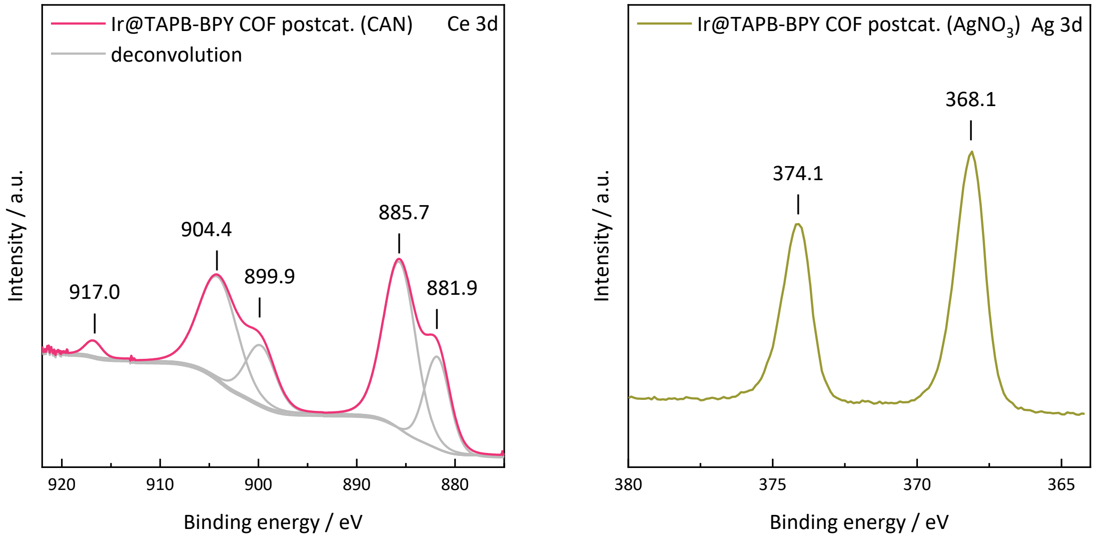

Figure S 31: Selected XPS spectra for Ir@TAPB-BPY COF after oxygen evolution experiments with CAN (78 mM, left) and $AgNO_3$ (10 mM, right). Note that silver species are hardly distinguishable *via* XPS, so we refrain from an assignment here.[37] The peak at 917.0 eV (Ce 3d) is ascribed to small amounts of Ce(IV), whereas the other peaks indicate the presence of Ce(III).[38–40] Upscaled reaction conditions were used to retrieve sufficient sample for these analyses – see section S1.

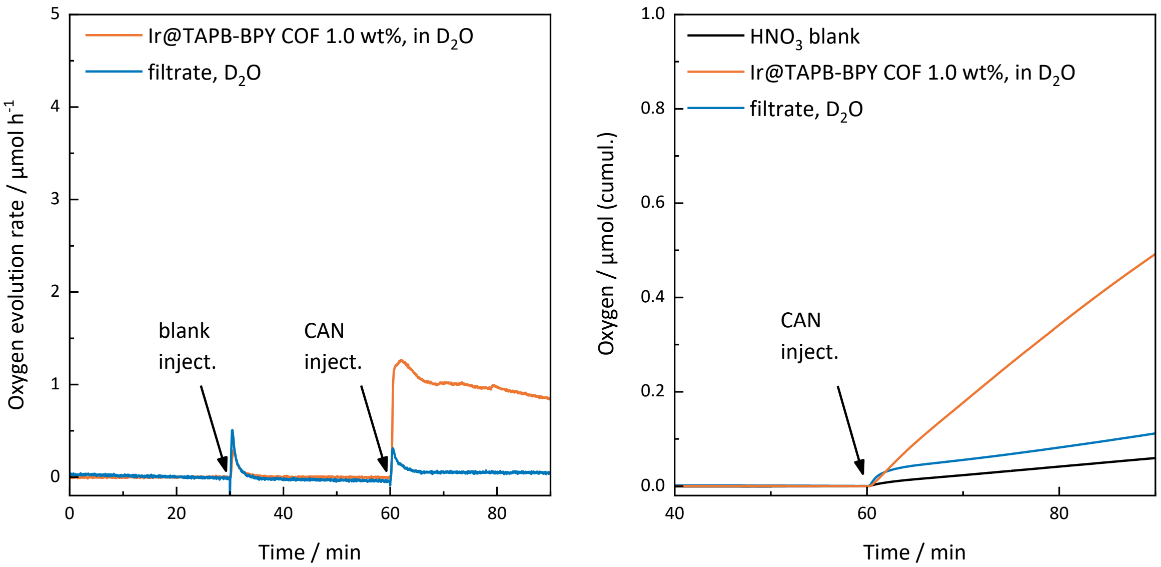

Figure S 32: Filtration experiment for the chemical oxygen evolution with Ir@TAPB-BPY COF. After catalysis, the COF was separated by filtration through a 0.45 μm PTFE syringe filter, and the filtrate was subjected to a new run in order to assess the catalytic activity of the detached iridium species. To this end, the recovered filtrate volume of 4.3 mL was restocked with 0.1 M DNO_3 to the starting volume of 4.6 mL in order to repeat the experiment with identical injection schemes. Original reaction conditions: 5 mg Ir@TAPB-BPY COF with 1.0 wt% Ir, 78 mM CAN (final conc.) in 5 mL DNO_3 (pH 1).

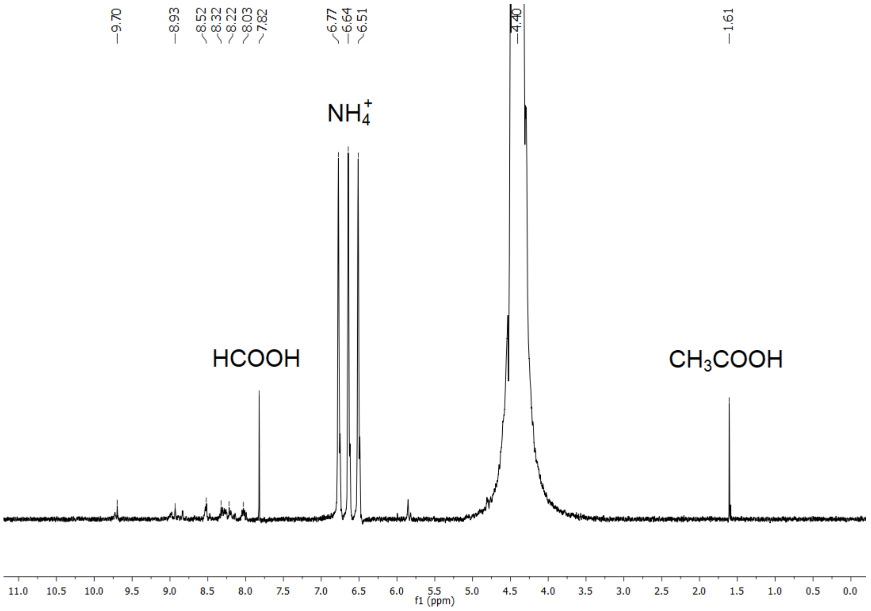

Figure S 33: ^{1}H NMR spectrum of the filtrate of Ir@TAPB-BPY COF after chemical water oxidation reaction with CAN (78 mM in pH 1 D_2O, see Figure S 32). Chemical shifts referenced to NH_4^+ according to Ref. [41].

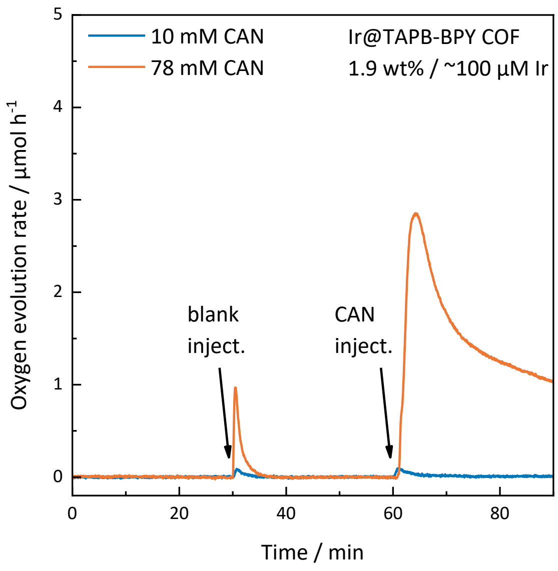

Figure S 34: Chemical water oxidation experiments with Ir@TAPB-BPY COF and CAN concentrations of 78 mM (orange) and 10 mM (blue). Reaction conditions: 5.0 mg Ir@TAPB-BPY COF, 78 or 10 mM CAN (pH 1 in aq. HNO_3, final volume 5 mL). For the 10 mM experiment, the injection volume was reduced to 50 µL to mitigate background oxygen detection.

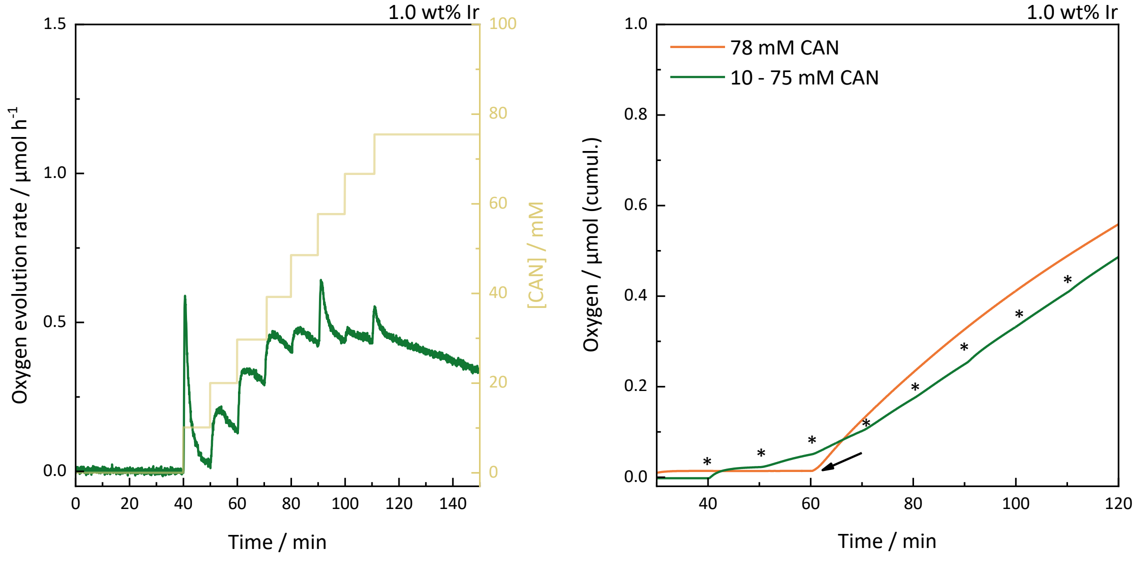

Figure S 35: Left: Chemical water oxidation experiment with Ir@TAPB-BPY COF and subsequent addition of CAN aliquots. Quickly declining signal suggest that the detected oxygen is not of catalytic nature, but partly caused by insufficiently degassed stock solution and/or syringe. Right: Comparison of oxygen evolved during fixed-concentration and dosing experiments. Reaction conditions: 5.0 mg Ir@TAPB-BPY COF (1.0 wt% Ir), 78 or 10 mM CAN (pH 1 in aq. HNO_3, final volume 5 mL). Asterisks mark subsequent addition of CAN (50 µL of a 1 M stock solution), whereas the arrow marks the injection of a single portion CAN, yielding the stated final concentration.

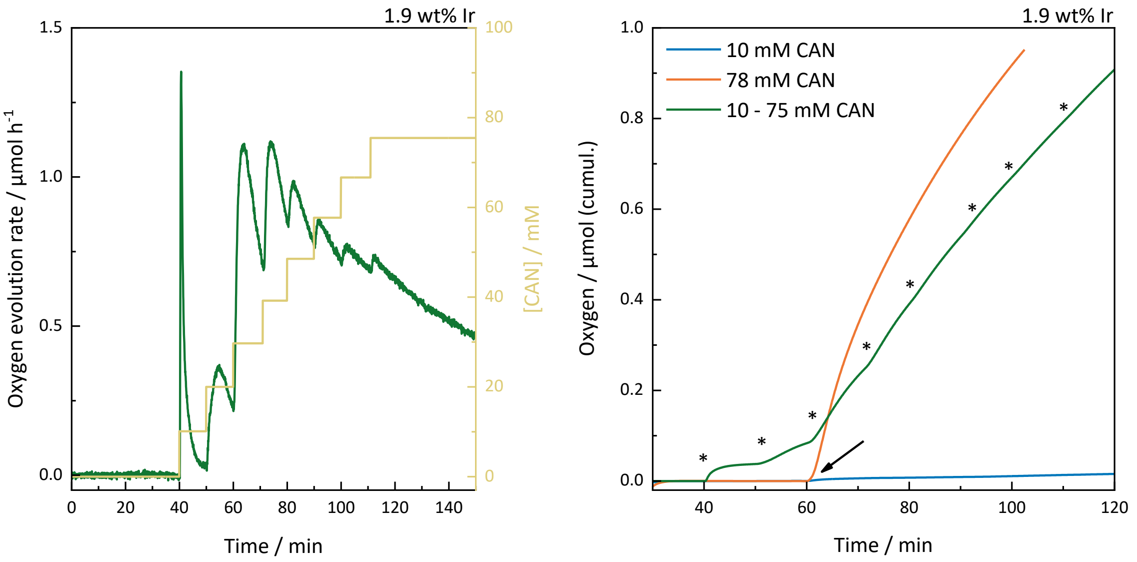

Figure S 36: Left: Chemical water oxidation experiment with Ir@TAPB-BPY COF and subsequent addition of CAN aliquots. The first, quickly declining signal suggests that the detected oxygen after the first injection is not of catalytic nature, but caused by insufficiently degassed stock solution and/or syringe. Right: Comparison of oxygen evolved during fixed-concentration and dosing experiments. Reaction conditions: 5.0 mg Ir@TAPB-BPY COF (1.9 wt% Ir), 78 or 10 mM CAN (pH 1 in aq. HNO_3, final volume 5 mL). Asterisks mark subsequent addition of CAN (50 µL of a 1 M stock solution), whereas the arrow marks the injection of a single portion CAN, yielding the stated final concentration.

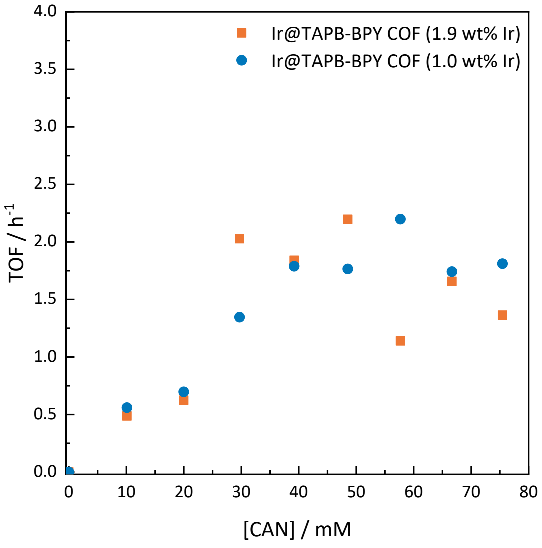

Figure S 37: Dependance of the catalytic oxygen evolution by Ir@TAPB-BPY COF on the CAN concentration.

Table S 1: ICP data for stability experiments with Ir@TAPB-BPY COF under chemical water oxidation conditions.

	pristine	No CAN	10 mM CAN	78 mM CAN
wt% Ir	1.8 wt%	2.0 wt%	1.7 wt%	1.3 wt%
Ir leakage	-	5-7%	10-12%	18-22%
Ce deposition	-	-	0.2 wt%	10.3 wt%

The statistic error of the ICP measurements was in the range of 0.1-0.2 wt% for the solids.

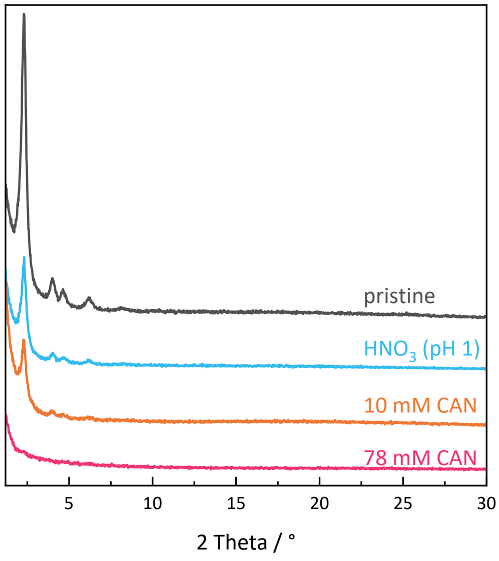

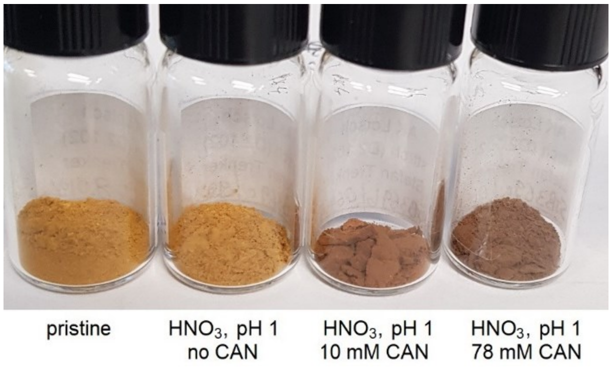

Figure S 38: Left: XRPD data for Ir@TAPB-BPY COF before and after stability tests under the stated conditions. Right: Photographic image of Ir@TAPB-BPY COF (1.9 wt% Ir) before and after stability tests under the stated conditions. For details, see Table S 1.

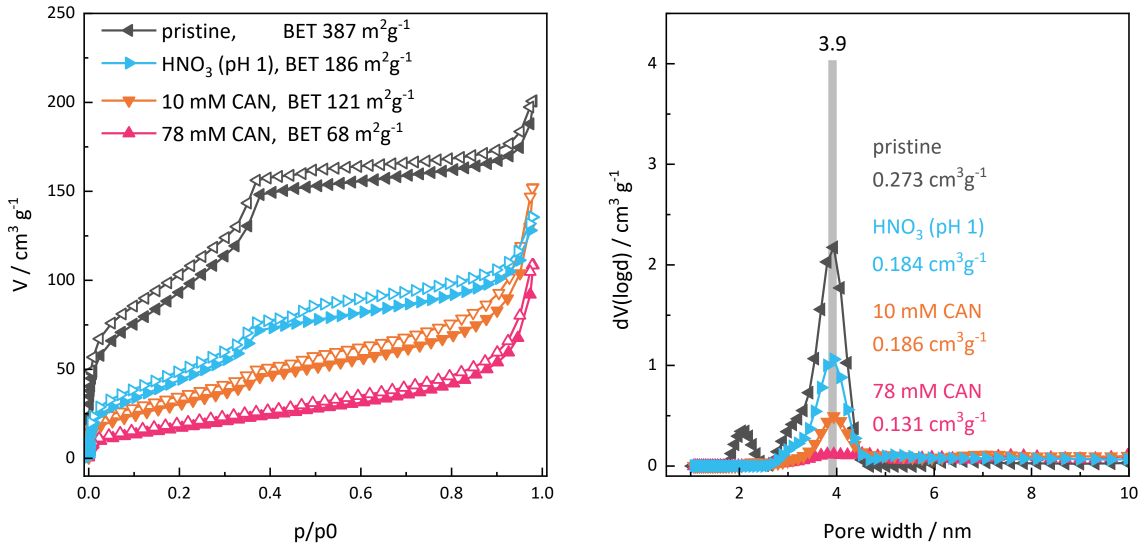

Figure S 39: Nitrogen sorption isotherms for Ir@TAPB-BPY COF (1.8 wt% Ir) before and after stability tests under the stated conditions (left). The pore size distribution (right) was obtained from a QSDFT kernel for cylindrical pores (adsorption branch). Pore volumes given as annotation.

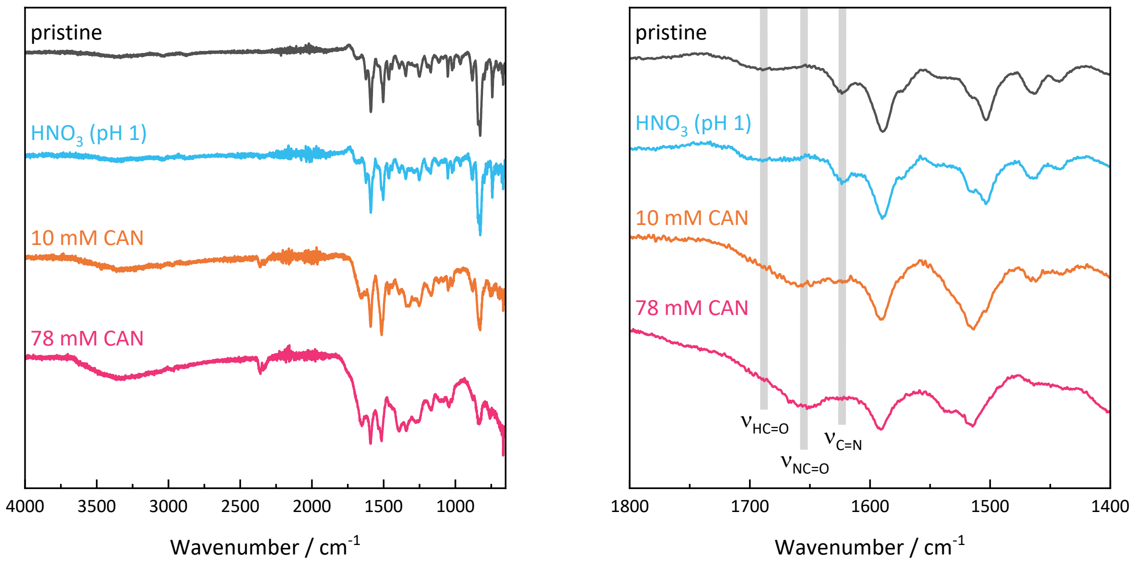

Figure S 40: FTIR spectra of Ir@TAPB-BPY COF after chemical water oxidation with CAN in varying concentrations.

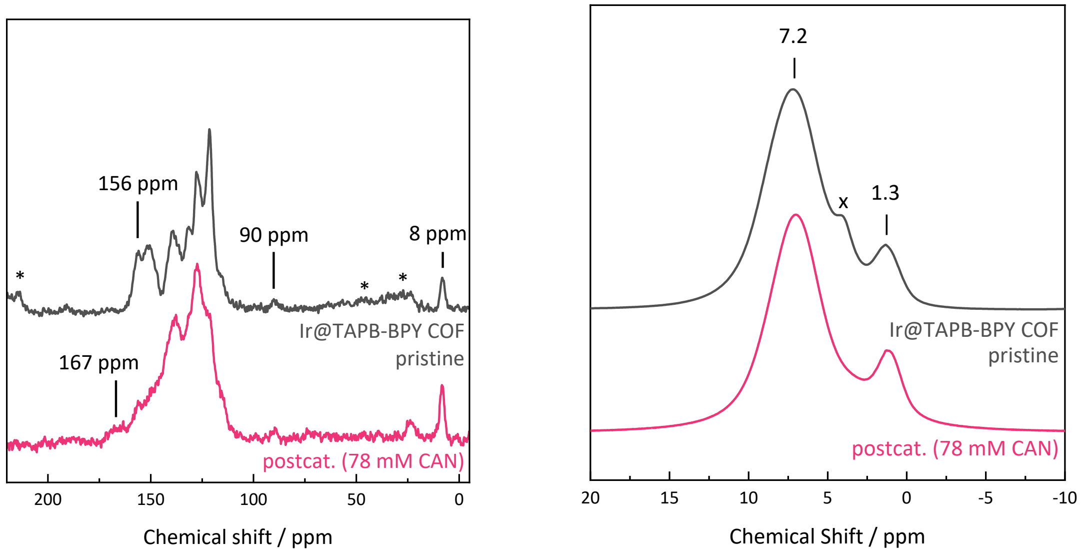

Figure S 41: 13C (left) and 1H (right) ssNMR spectra of Ir@TAPB-COF (4.7 wt% Ir) before and after oxygen evolution experiments with CAN (78 mM, pH 1). Asterisks mark spinning side bands, cross marks residual water signal. Upscaled reaction conditions were used to retrieve sufficient sample for the postcatalytic analysis – see section S1.

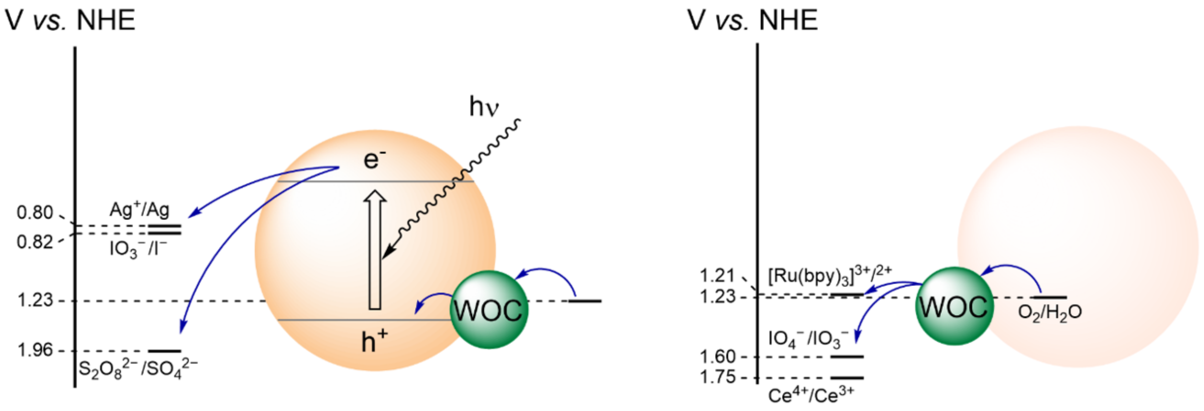

Figure S 42: Schematic illustration of SEA redox potentials in water oxidation catalysis driven photocatalytically (left) and chemically (right). Not to scale. Values for primary oxidants taken from Ref [42], values for photocatalysis SEAs derived from Ref [43] and Ref [44].

S5 - Photocatalytic Oxygen Evolution

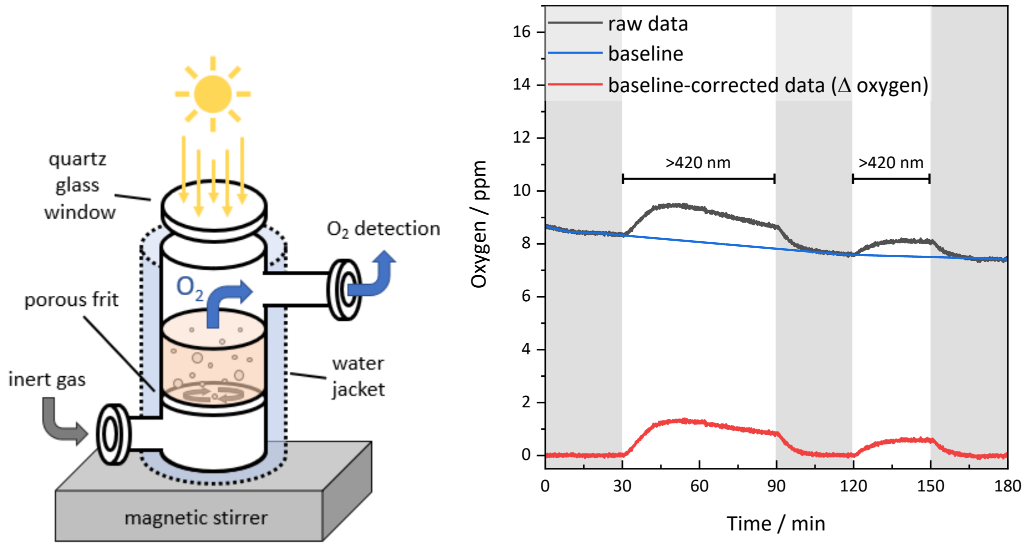

Figure S 43: Flow reactor used for photocatalytic oxygen evolution experiments (left). Exemplary representation of the background correction usually applied to raw oxygen readouts obtained from fluorescent sensor spots under flow conditions (right).

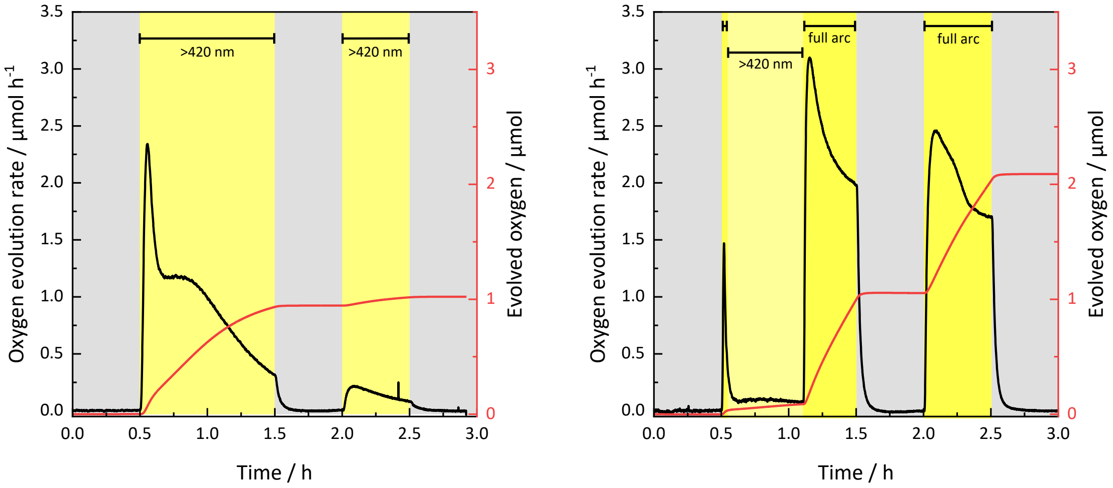

Figure S 44: Positive control experiments for photocatalytic oxygen evolution. Illumination with a 300 W Xe lamp and optical filters as annotated. Left: 0.1 mM [Cp*Ir(bpy)Cl]Cl, 1 mM $[Ru(bpy)_3]Cl_2$, 20 mM $Na_2S_2O_8$, Right: 5.02 mg TiO_2 Aeroxid, $RuCl_3$ (50 µg Ru), 10 mM $NaIO_3$.

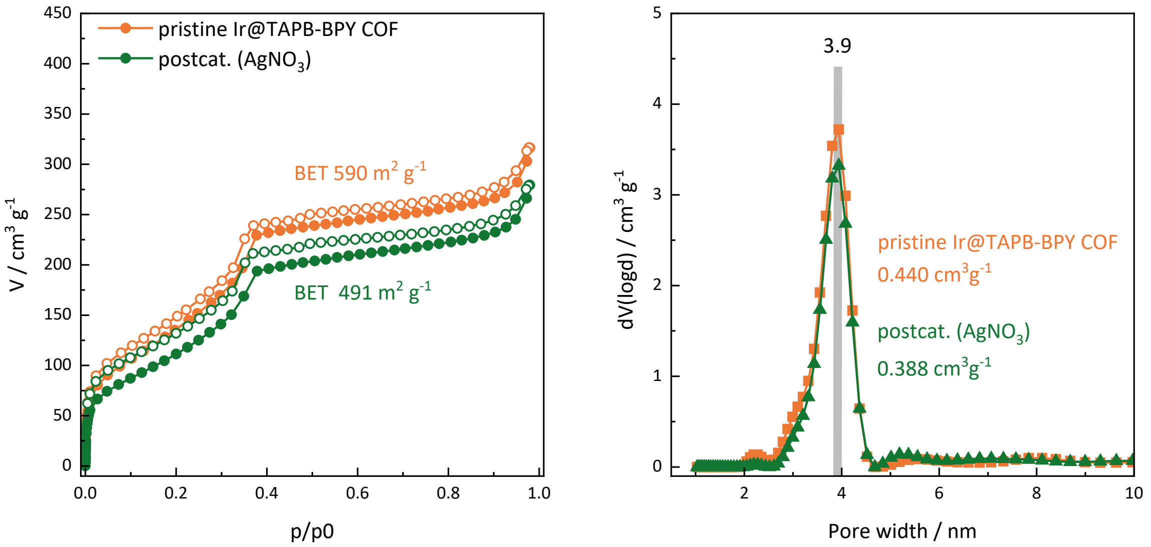

Figure S 45: Nitrogen sorption isotherms at 77 K (left) and pore size distribution (right) for Ir@TAPB-BPY COF (1 wt% Ir) before and after photocatalytic oxygen evolution experiments with 10 mM $AgNO_3$. Filled and open symbols represent the adsorption and the desorption branches, respectively. The pore size distribution was obtained from a QSDFT kernel for cylindrical pores (adsorption branch). Upscaled reaction conditions were used to retrieve sufficient sample for this analysis – see section S1.

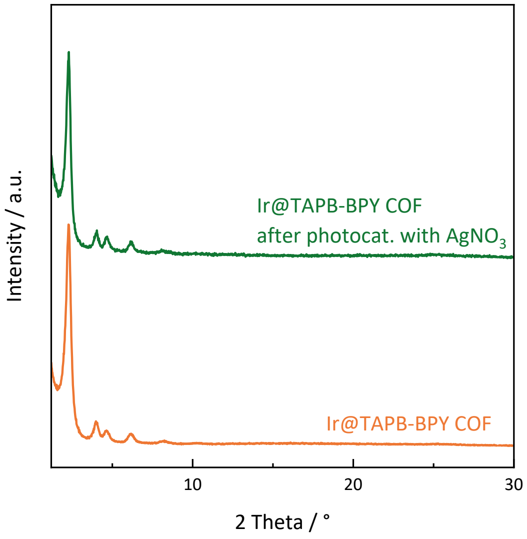

Figure S 46: XRPD patterns for Ir@TAPB-BPY COF (1 wt% Ir) before and after photocatalysis in 10 mM $AgNO_3$ under visible light. Upscaled reaction conditions were used to retrieve sufficient sample for this analysis – see section S1.

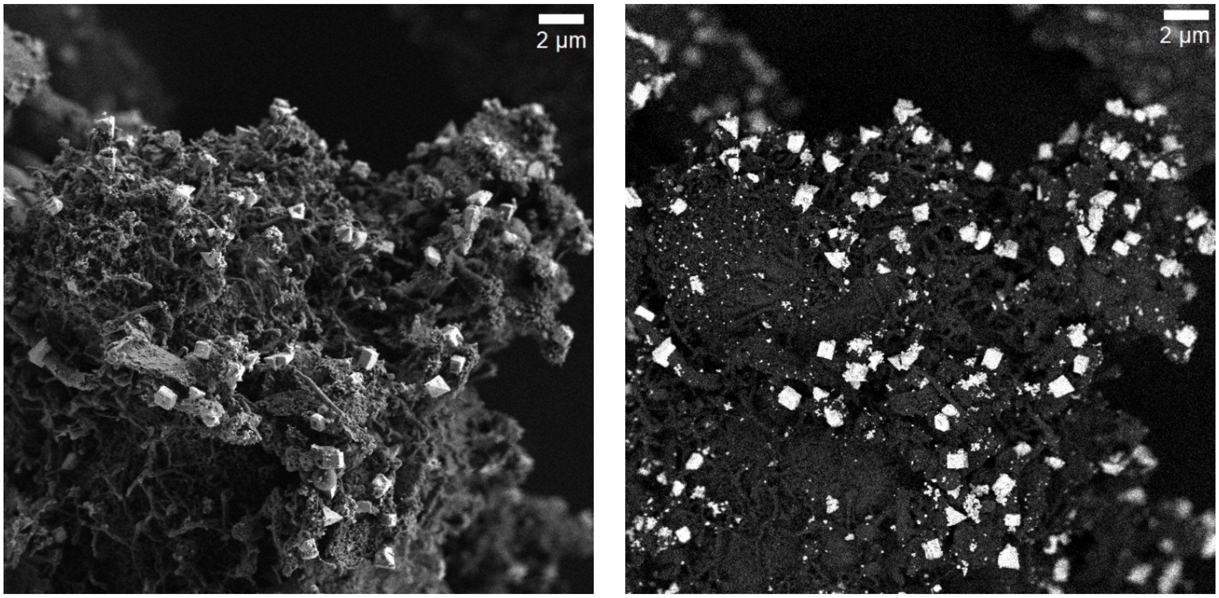

Figure S 47: SEM images of Ir@TAPB-BPY COF after photocatalysis experiments with $AgNO_3$ with secondary electron detection (left) and energy selective backscattered electron detection (right). Upscaled reaction conditions were used to retrieve sufficient sample for this analysis – see section S1.

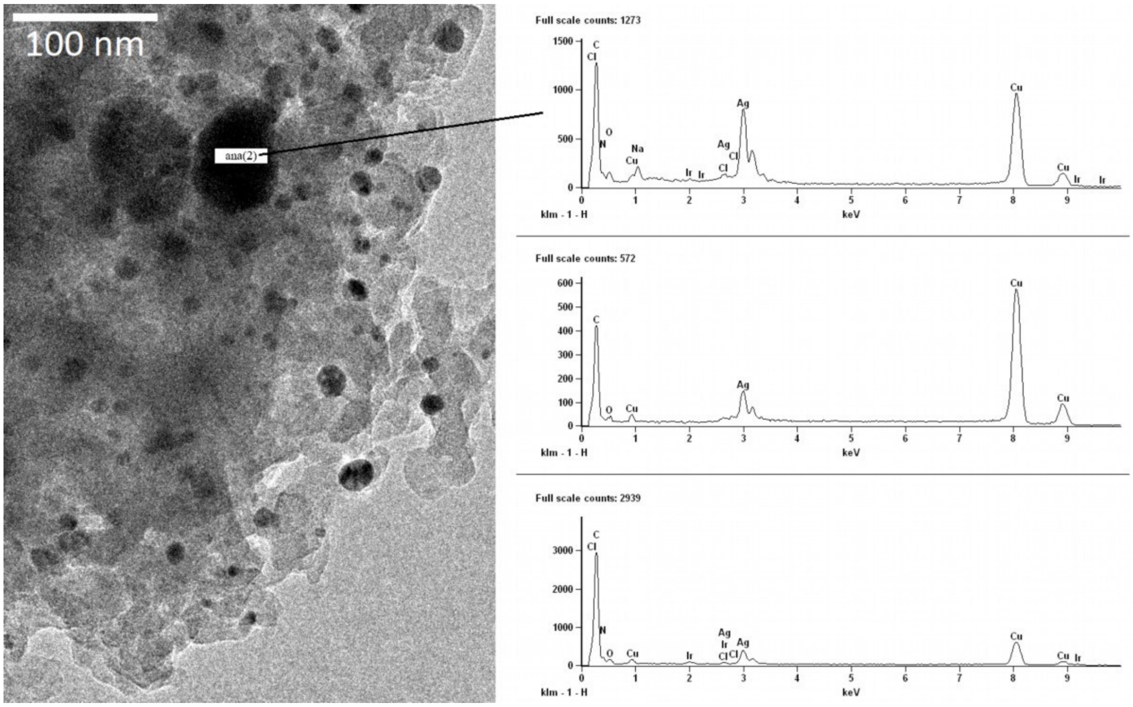

Figure S 48: Overview TEM image of Ir@TAPB-BPY COF after photocatalysis experiments with $AgNO_3$ (left). EDX spectra (right) show the elemental composition at different positions on the COF, one of which is also present on the left image (top EDX spectrum). Upscaled reaction conditions were used to retrieve sufficient sample for this analysis – see section S1.

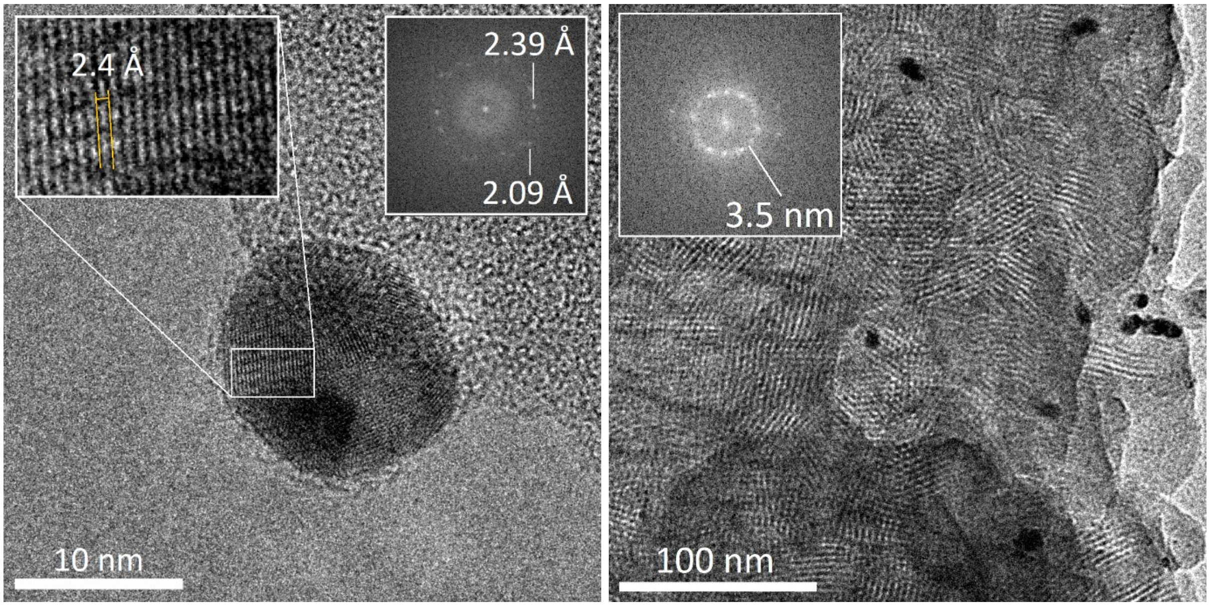

Figure S 49: TEM images of Ir@TAPB-BPY COF after photocatalysis experiments with $AgNO_3$ showing the presence of crystalline nanoparticles (left) and the retained porosity of the COF (right). Insets show FFT or zoomed-in parts of the respective images. Upscaled reaction conditions were used to retrieve sufficient sample for this analysis – see section S1.

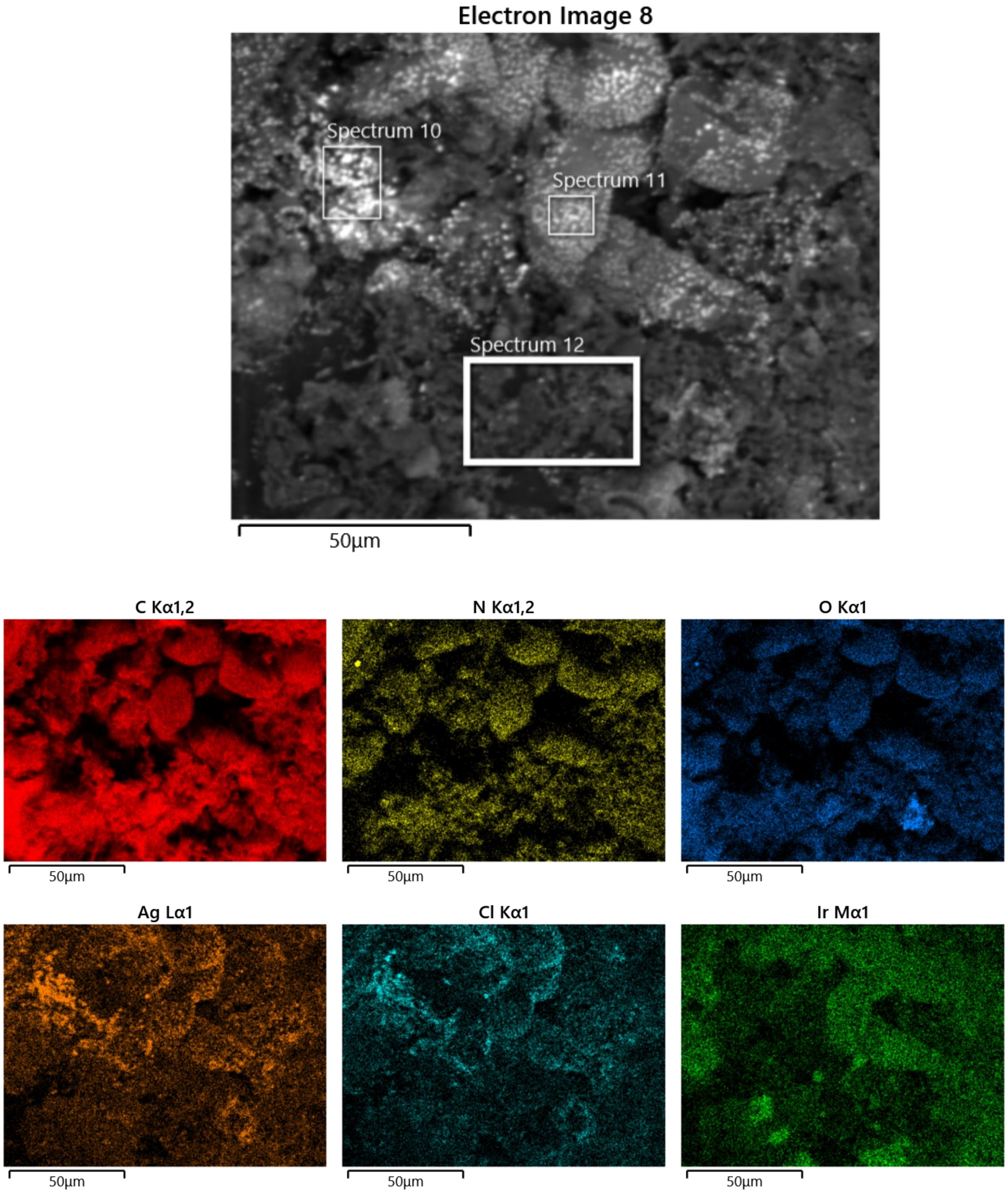

Figure S 50: SEM elemental mapping of Ir@TAPB-BPY COF after photocatalysis experiments with $AgNO_3$.

Table S 2: EDX analysis for Ir@TAPB-BPY COF after photocatalytic water oxidation experiments with $AgNO_3$. Values given in at%. EDX spectra measured at the positions specified in Figure S 50.

Element	Spectrum 10	Spectrum 11	Spectrum 12
N	47.94	52.35	43.29
O	20.35	31.45	41.70
Ir	0.40	1.91	1.09
Cl	8.66	4.12	3.08
S	-	-	1.07
Ag	21.81	10.16	8.08
Na	-	-	1.07
Si	0.84	-	0.61
Total	100	100	100

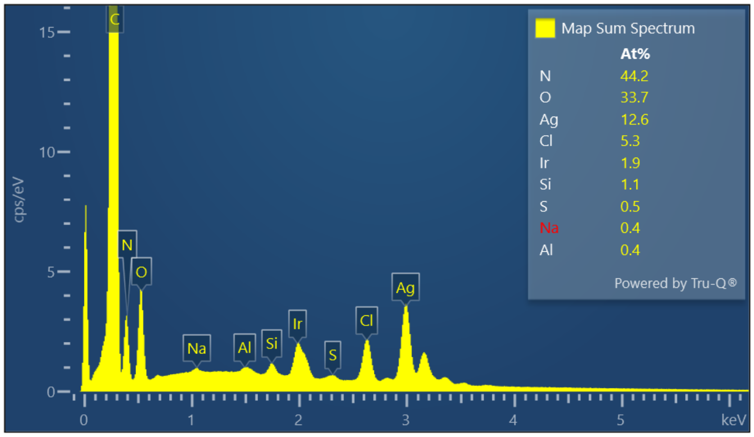

Figure S 51: Summed-up EDX spectra for the elemental mapping of Ir@TAPB-BPY COF after photocatalysis experiments with $AgNO_3$.

Table S 3: EDX analysis for Ir@TAPB-BPY COF before and after (photo-)catalytic water oxidation experiments with varying SEAs (map sum spectra). Values given in at%.

Element	pristine	$Na_2S_2O_8$[a]	$AgNO_3$[b]
N	47.88	36.54	44.21
O	39.53	50.31	33.66
Ir	4.05	3.47	1.93
Cl	6.25	2.26	5.32
Ce	-	-	-
S	0.41	3.70	0.51
Ag	-	-	12.55
Na	0.30	0.31	0.38
Al	0.55	0.47	0.37
Si	1.02	2.94	1.06
Total	100	100	100

a: 20 mM, b: 10 mM

S6 - Photocatalytic Oxygen Evolution Experiments with Sodium Persulfate as SEA

We found that irradiation of a persulfate solution leads to significant oxygen evolution under unfiltered illumination with a 300 W Xenon arc lamp (Figure S 52). Bahnemann suggests that the sulfate radicals derived from persulfate reduction (eq. S4) subsequently oxidize water and release oxygen *via* hydroxyl radicals (eq. S5-S6).[43,45] In our studies, oxygen evolution was most pronounced in the absence of both photosensitizer and eventual cocatalyst (Table S 4, entry 1), implying primarily homolytic cleavage of $S_2O_8^{2-}$ (eq. S7) takes place rather than photoreduction.[46,47]

$$S_2O_8^{2-} + e^- \rightarrow SO_4^{\cdot-} + SO_4^{2-} \qquad \text{(eq. S4)}$$

$$SO_4^{\cdot-} + H_2O \rightarrow SO_4^{2-} + OH^{\cdot} + H^+_{aq.} \qquad \text{(eq. S5)}$$

$$2\,OH^{\cdot} \rightarrow H_2O_2 \rightarrow H_2O + \frac{1}{2}O_2 \qquad \text{(eq. S6)}$$

$$S_2O_8^{2-} + h\nu \rightarrow 2\,SO_4^{\cdot-} \qquad \text{(eq. S7)}$$

On the contrary, we did not detect significant amounts of oxygen with metal-free TAPB-BPY COF in the presence of $Na_2S_2O_8$ (Table S 4, entry 2). We presume that the COF quenches the decomposition pathway through reaction with the highly reactive sulfate radicals, which are known to oxidize organics.[48,49] Iridium-loaded TAPB-BPY COF, however, shows apparent oxygen evolution, even over the course of 14 hours (Table S 4, entry 3, Figure S 53). This opposing behavior for pristine and metalated TAPB-BPY COF points out the importance of adequate blank measurements – otherwise, the oxygen evolution in the case of Ir@TAPB-BPY COF might have been mistaken as the results of catalytic water oxidation, although it is presumably caused by decomposition of the SEA. However, in the case of $S_2O_8^{2-}$ such misinterpretations can easily be avoided through the use of 420 nm longpass filters since persulfate does not decompose when irradiated with visible light.

Table S 4: Photocatalytic water oxidation experiments with persulfate under full arc illumination.

Entry	Persulfate	COF	Oxygen evolution, full arc	Oxygen evolution, AM 1.5
1	20 mM	-	1.5 µmol h^{-1}	0.1 µmol h^{-1}
2	20 mM	TAPB-BPY COF	0 µmol h^{-1}	0 µmol h^{-1}
3	20 mM	Ir@TAPB-BPY COF	0.7 µmol h^{-1}	0 µmol h^{-1}
4	-	Ir@TAPB-BPY COF	0 µmol h^{-1}	0 µmol h^{-1}

Reaction conditions: 5 mg COF, 5 mL total volume, 300 W Xenon lamp, filters as annotated.

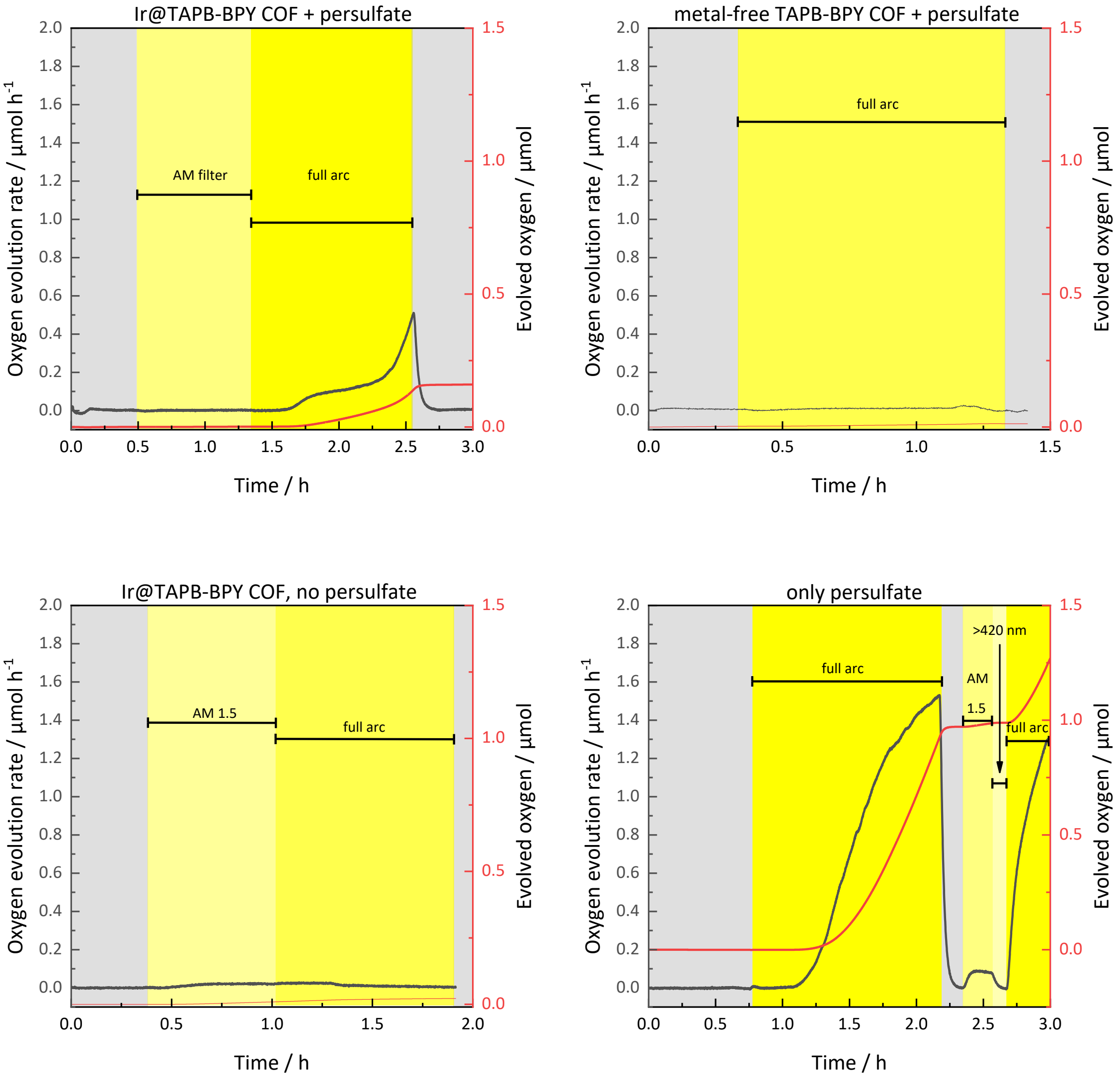

Figure S 52: Oxygen evolution rates for photocatalytic reactions with persulfate. Reaction conditions: 5.0 mg COF, 5.0 mL 20 mM $Na_2S_2O_8$, 300 W Xenon lamp, filters as annotated.

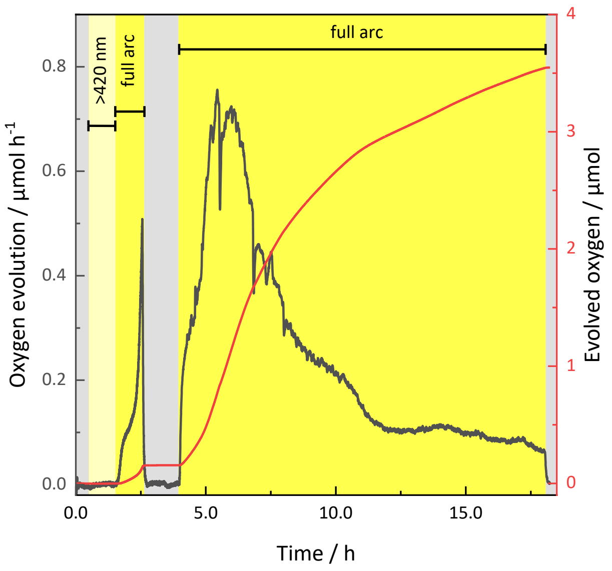

Figure S 53: Long-time photocatalytic oxygen evolution experiment with Ir@TAPB-BPY COF. Reaction conditions: 5 mg COF, 5 mL 20 mM $Na_2S_2O_8$, 300 W Xenon lamp.

S7 - Silver Nitrate Decomposition

We found that illumination of a suspension of Ir@TAPB-BPY COF in aqueous silver nitrate with a conventional 300 Xe lamp leads to distinct oxygen evolution of 0.1 – 0.4 µmol h^{-1} depending on the incident wavelength range (Figure S 54a). Both AM 1.5 filtered and unfiltered full spectral illumination gave somewhat higher rates than visible light (>420 nm) which does not exceed values of 0.2 µmol h^{-1}. Surprisingly, when conducting a control measurement with only silver nitrate solution and visible light we obtained an oxygen trace similar to those of both Ir@TAPB-BPY and TAPB-BPY COF under the same conditions (Figure S 54b). Given that both photosensitizer and WOC are missing in this experiment, no oxygen evolution was expected to take place.

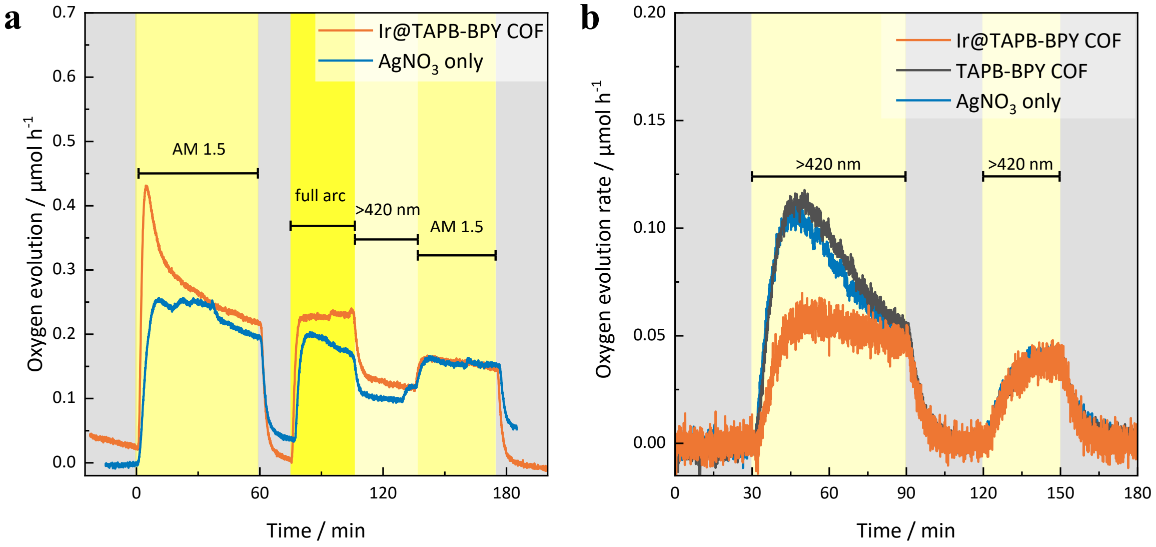

Figure S 54: Photocatalysis experiments with $AgNO_3$ as sacrificial electron acceptor. Reaction conditions: 5.0 mg COF, $AgNO_3$ (10 mM, 5.0 mL water), illumination with a 300 W Xe lamp and optical filters as specified. Grey areas represent dark reaction conditions.

We thus set out to understand this finding and elucidate the exact role of silver nitrate in photocatalysis. To this end, we repeated the illumination of a 10 mM $AgNO_3$ solution with complementary oxygen detection *via* gas chromatography (GC). For higher reproducibility and spectral match, we use a class AAA solar simulator with integrated AM1.5 filter. Overlapping data for both oxygen detection methods suggests that oxygen indeed evolves from silver nitrate solutions without an external photosensitizer (Figure S 55). A duplicate experiment confirms the reproducibility.

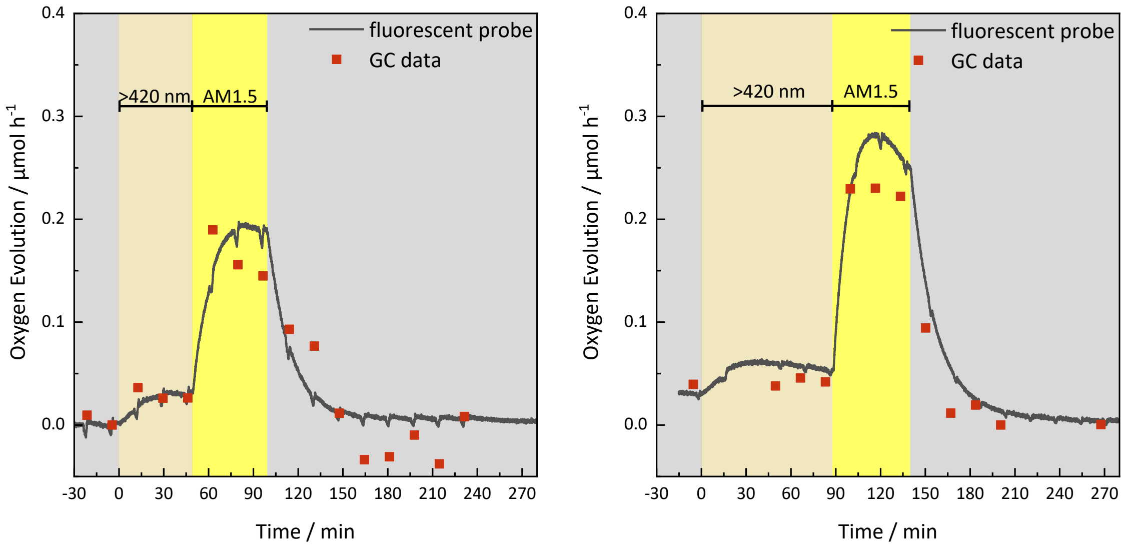

Figure S 55: Oxygen evolution during irradiation of 10 mM $AgNO_3$ solutions with varying optical filters as annotated. Simultaneous measurement *via* gas chromatography and fluorescent sensors under flow conditions. Periodic peaks are due to pressure variations upon GC sampling. Reaction conditions: 5 mL, AAA solar simulator, 1 sun, optical filters as annotated.

Upon visual inspection, we could find grey deposits on the reactors glass frit and O-rings (Figure S 56). To gain mechanistic insights, we analyzed a 10 mM $AgNO_3$ solution after illumination with visible light (>420 nm) for 90 minutes. TEM imaging of the precipitate after illumination shows the existence of particles with sizes in the range of 10 – 50 nm (Figure S 59). A scaled-up photolysis of 10 mM $AgNO_3$ with AM1.5 illumination and subsequent XRPD analysis reveals that the deposition consists of elemental silver (Figure S 59).

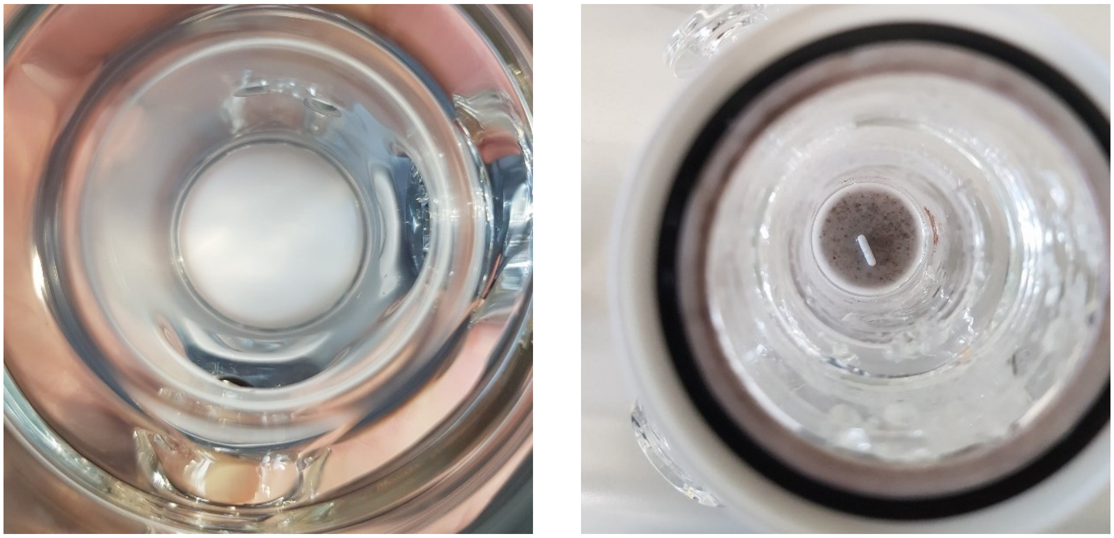

Figure S 56: Photographic image of a flow-reactor before (left) and after (right) photolysis of silver nitrate solutions (10 mM, AM1.5, 2 h).

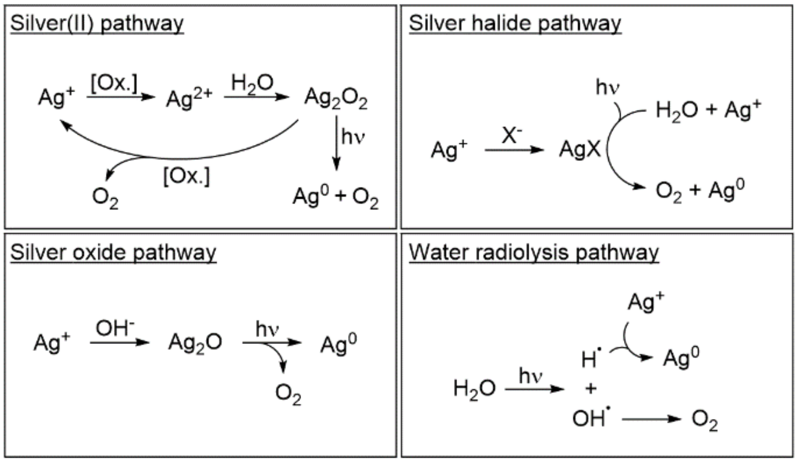

Figure S 57: Potential pathways for oxygen evolution from silver(I) species.

There are several pathways that could explain both the observed oxygen evolution and the formation of metallic silver (Figure S 57). BAHNEMANN *et al*. suggest silver peroxides as a source of oxygen during photocatalysis with Ag^{+} species, which can form through undesired oxidation of Ag(I) to Ag(II) in aqueous solutions.[45,50] Subsequent oxygen evolution reactions *via* Ag^{3+} – formed through dismutation of Ag^{2+} – can also be envisaged.[51,52] However, given the high redox potential for the Ag(II)/Ag(I) couple (E^0 = 1.98 V), either a strong oxidizer or a low-valence band semiconductor would be needed to generate Ag^{2+} – both of which are missing in our experiment.[51] Nevertheless, we used electron paramagnetic resonance (EPR) spectroscopy to test for the presence of paramagnetic Ag^{2+} species, but neither *in-situ* nor *ex-situ* illumination of silver nitrate solutions in varying concentrations yielded the signals expected for Ag^{2+} (Figure S 58).[51,53,54]

A special pathway for oxygen evolution from Ag^{+} solutions arises in the presence of halides due to the formation of hardly soluble AgX salts. Recently, is has been shown by the MAEDA group that silver nitrate can act both as SEA and precursor to photosensitizing AgCl particles in one system. Upon reaction of $AgNO_3$ with $[Co(NH_3)_6]Cl_3$ silver chloride particles form, which act as photosensitizer for the oxygen evolution reaction with the remaining $[Co(NH_3)_6]^{3+}$ species as WOC.[55] Similarly, we also note the formation of AgCl particles when employing Ir@TAPB-BPY COF as photocatalyst with $AgNO_3$ as SEA due to the presence of chloride ligands and counterions (Figure S 47 - Figure S 51). However, since the presence of chloride or other halides is detrimental for this OER pathway, it cannot explain the decomposition of silver nitrate solutions in ultrapure water in the absence of Ir@TAPB-BPY COF.

In addition, we considered Ag_2O as a potential intermediate in the photodecomposition of $AgNO_3$ (Figure S 57).[51,56] We thus performed a control experiment with Ag_2O to assess its photostability and eventual accompanied oxygen evolution. In fact, upon illumination of a 5 mM suspension of Ag_2O in water, we were able to detect small amounts of oxygen with evolution rates around 0.05 µmol h^{-1} (AM1.5, Figure S 60). In accordance with the proposed mechanism (Figure S 57), we could detect traces of elemental silver after Ag_2O photolysis (Figure S 60). However, the comparably small oxygen evolution rate even in the presence of pure Ag_2O suggests that its role

as an intermediate in the investigated $AgNO_3$ decomposition pathway is negligible. Moreover, we regard the formation of significant amounts of Ag_2O from aqueous solutions of $AgNO_3$ at pH 7 to be unlikely, as this process usually requires more alkaline conditions.[51,56,57]

As a fourth pathway, we assessed the photoreduction of Ag^+ – and the accompanying water oxidation – as the cause of oxygen evolution from silver nitrate solutions. In fact, photolysis and radiolysis of silver(I) solutions has been established as a useful tool to synthesize silver nanoparticles, -clusters, and other silver structures.[58–62] Mechanistically, it is argued that excitation of H_2O leads to dissociation into reactive species such as $OH^•$, $H^•$, and e_{aq}^-, with the latter two being able to reduce Ag(I) to Ag(0).[62–64] The $OH^•$ radical on the other hand can subsequently lead to oxygen evolution, which was qualitatively confirmed by HADA *et al.* in 1976 when irradiating aqueous silver perchlorate solutions with 253.7 nm light.[65] However, we were not able to detect supposedly oxygen evolving $OH^•$ radicals *via* EPR spectroscopy with the spin trap 5,5-dimethyl-1-pyrroline-*N*-oxide (DMPO, Figure S 58). In an indirect approach, we added isopropanol as hydroxyl radical scavenger to a 10 mM $AgNO_3$ solution and in fact detected less oxygen upon illumination, which hints to $OH^•$ radicals to play a role in the undesired oxygen evolution from $AgNO_3$, as proposed in the water radiolysis pathway (Figure S 57, Figure S 62).[66]

In wavelength-dependent literature studies it was found that longer wavelengths of up to 405 nm light can still photoreduce silver ions, though with slower kinetics.[67,68] Indeed, we also found higher oxygen evolution rates when illuminating a silver nitrate solution with shorter wavelength light (Figure S 61). We pinned down the onset wavelength for this process to the range of 455-420 nm.

In summary, we could not find direct evidence that either of the four deceived $AgNO_3$ decomposition pathways underlies the significant oxygen evolution we observed (Figure S 55). Ultimately, we tried to rule out our flow setup itself (Figure S 43) as the source of the OER. Surprisingly, when conducting $AgNO_3$ photolysis in a bulk photoreactor, we could not detect increased amounts of oxygen upon illumination (Figure S 63). The addition of a loose glass frit to the bulk reactor did also not lead to oxygen evolution, ruling out the borosilicate frit material as unexpected photosensitizer and/or WOC. In follow-up experiments with a pristine flow reactor we could again not see significant oxygen evolution – in contrast to the usual "recycled" flow-through photoreactor (Figure S 64, Figure S 65).

We thus deduced the photoreactor to be contaminated despite extensive cleaning after every experiment. Upon extraction of the top layer of the borosilicate frit and subsequent optical and electron microscopy we identified ruthenium-loaded tungsten oxide as the most likely reason for the observed photocatalytic oxygen evolution in some cases (Figure S 66-Figure S 69). We note that the absorptance of $RuO_2@WO_3$ matches the onset wavelength in our wavelength-dependent oxygen evolution experiment (Figure S 61, *vide supra*).[30] More importantly, experiments with $RuO_2@WO_3$ were conducted in our research group earlier, which explains the origin of this catalytically active contaminant.

Similar to the $AgNO_3$ blank experiments, photocatalysis experiments with Ir@TAPB-BPY COF conducted in a pristine reactor also indicate no significant O_2 generation (Figure S 71), suggesting

that its previously observed activity (Figure S 54) can be solely ascribed to contaminations. Note that RuO_2@WO_3 nanoparticles are not active under chemical water oxidation conditions and thus not refute the catalytic activity of Ir@TAPB-BPY COF when using CAN (see main text, Figure S 70).

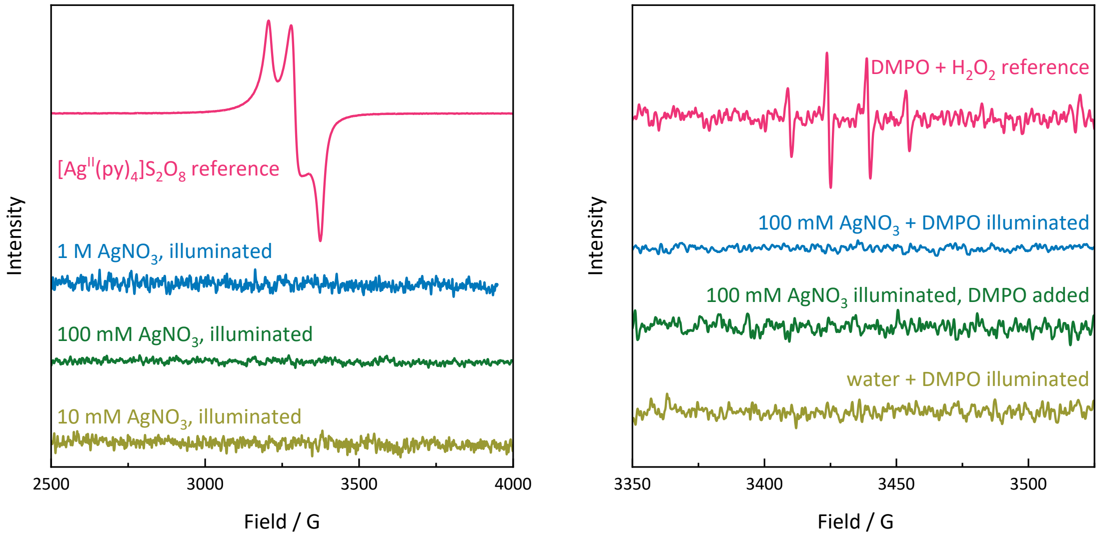

Figure S 58: EPR spectra for the detection of paramagnetic metal species (left) and organic radicals (right).

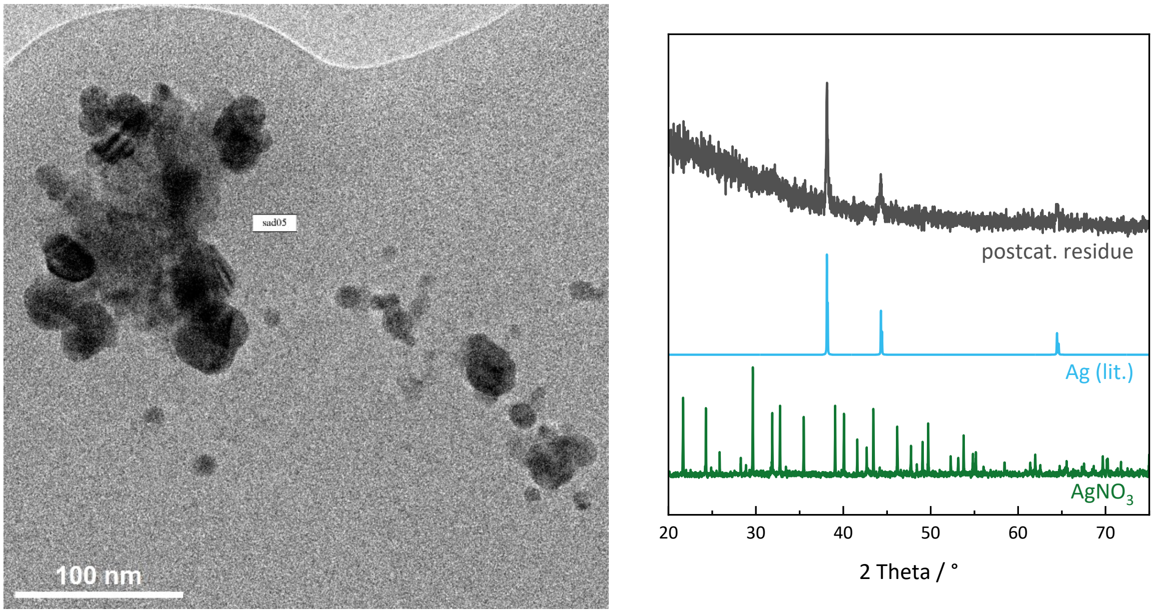

Figure S 59: TEM images (left) and XRPD pattern (right) of particles obtained from $AgNO_3$ photolysis (10 mM). Silver reference: ICSD 64994.[69]

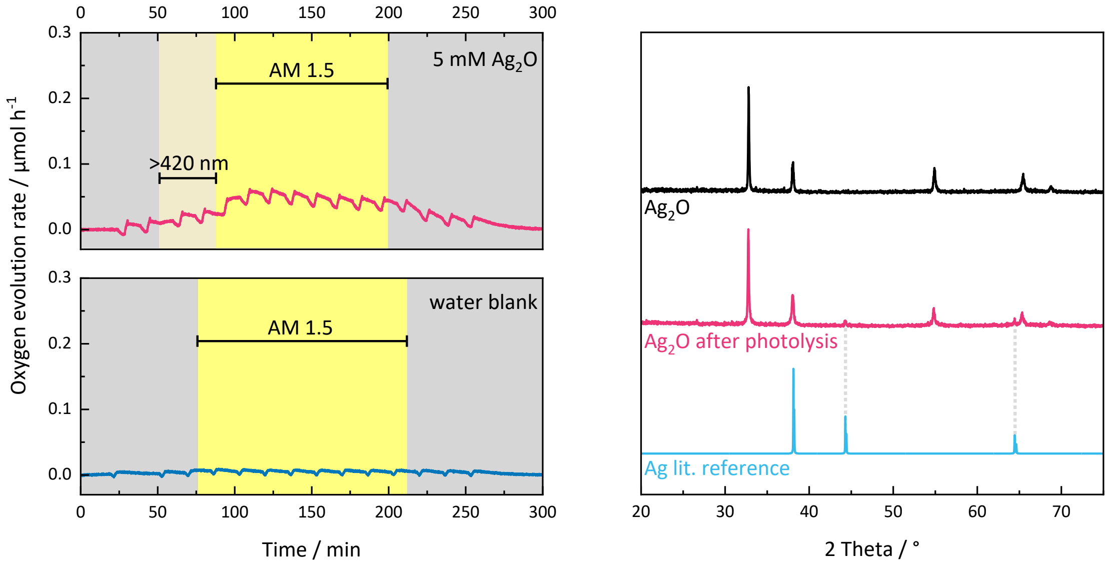

Figure S 60: Oxygen evolution by an Ag_2O suspension (5 mM) (left) and XRPD pattern (right) of the remaining particles. Photolysis experiment for pure water shown for comparison. Periodic peaks are due to pressure variations upon GC sampling. Silver reference: ICSD 64994.[69]

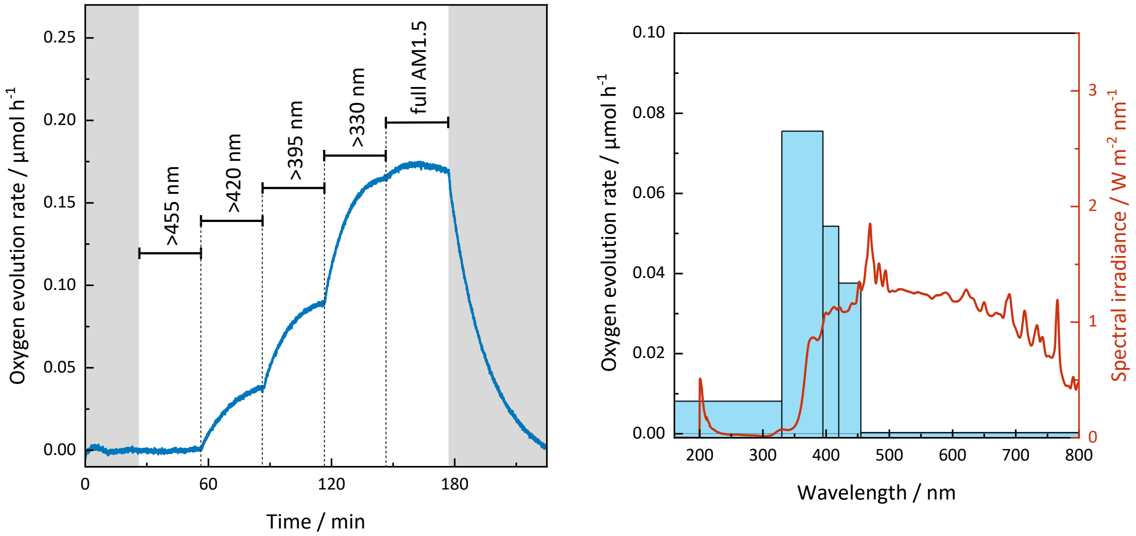

Figure S 61: Wavelength-dependence of $AgNO_3$ photolysis measured during longpass-filtered illumination (AAA solar simulator, 1 sun, AM1.5). Bar graph shows differential oxygen evolution, obtained by subtracting the oxygen evolution of longer wavelength illumination periods from the subsequent rate with shorter wavelength.

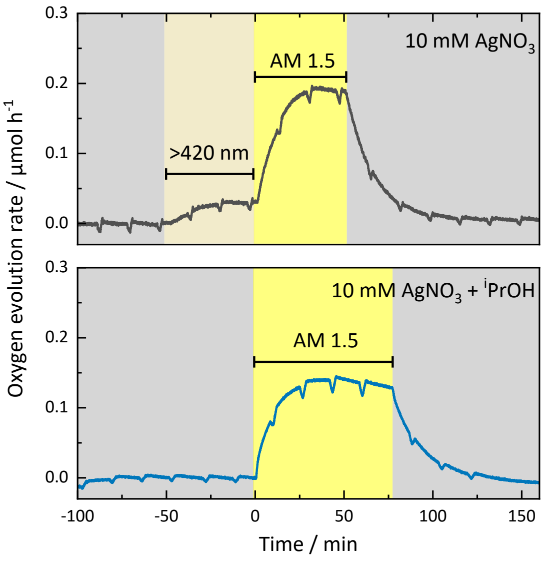

Figure S 62: Photolysis of $AgNO_3$ (10 mM) in the absence (top) and presence (bottom) of isopropanol (0.2 M) as hydroxyl radical scavenger. Periodic peaks are due to pressure variations upon GC sampling.

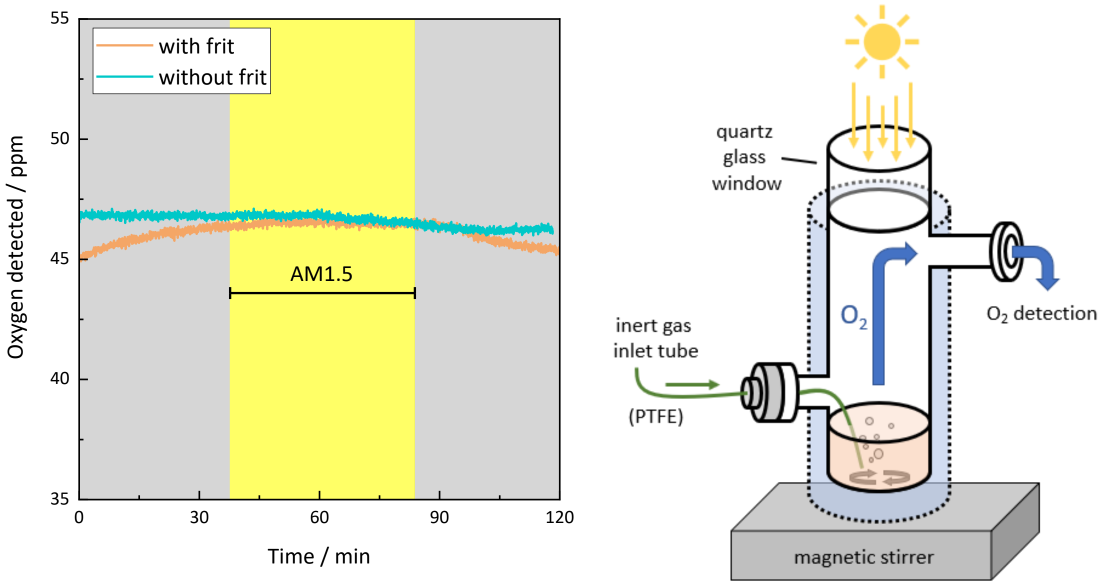

Figure S 63: Control photolysis experiment with $AgNO_3$ (10 mM) in a bulk photoreactor with and without additional loose glass frit (borosilicate, por. 4, 20 mm diameter, 2.5 mm thick, pristine, left) submerged in the solution. Oxygen readouts are given on the original ppm scale. Schematic illustration of the experimental setup with the bulk photoreactor (right).

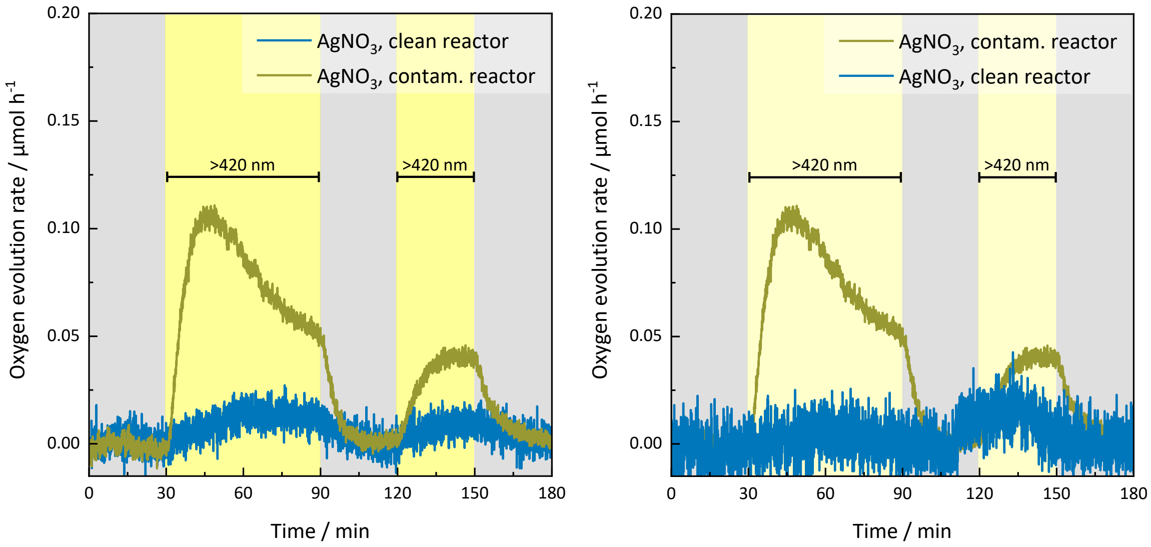

Figure S 64: Comparison of $AgNO_3$ (10 mM) photolysis experiments in a contaminated (ocre) and a pristine flow photoreactor (blue). Reaction conditions: $AgNO_3$ (10 mM, 5 mL), 300 W Xe lamp with installed >420 nm longpass filter. Grey areas represent dark reaction conditions.

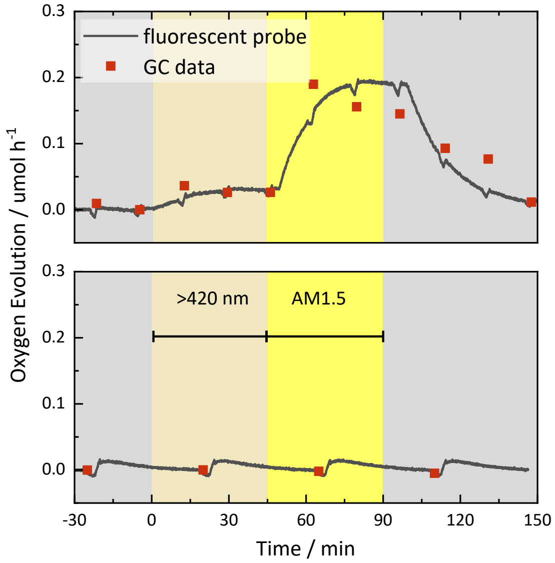

Figure S 65: Comparative photolysis of silver nitrate solutions in a contaminated (top) and a pristine flow photoreactor (bottom). Reaction conditions: 10 mM $AgNO_3$, 5 mL, AAA solar simulator, 1 sun, optical filters as annotated. Simultaneous oxygen measurement *via* gas chromatography and fluorescent sensors under flow conditions. Periodic peaks are due to pressure variations upon GC sampling.

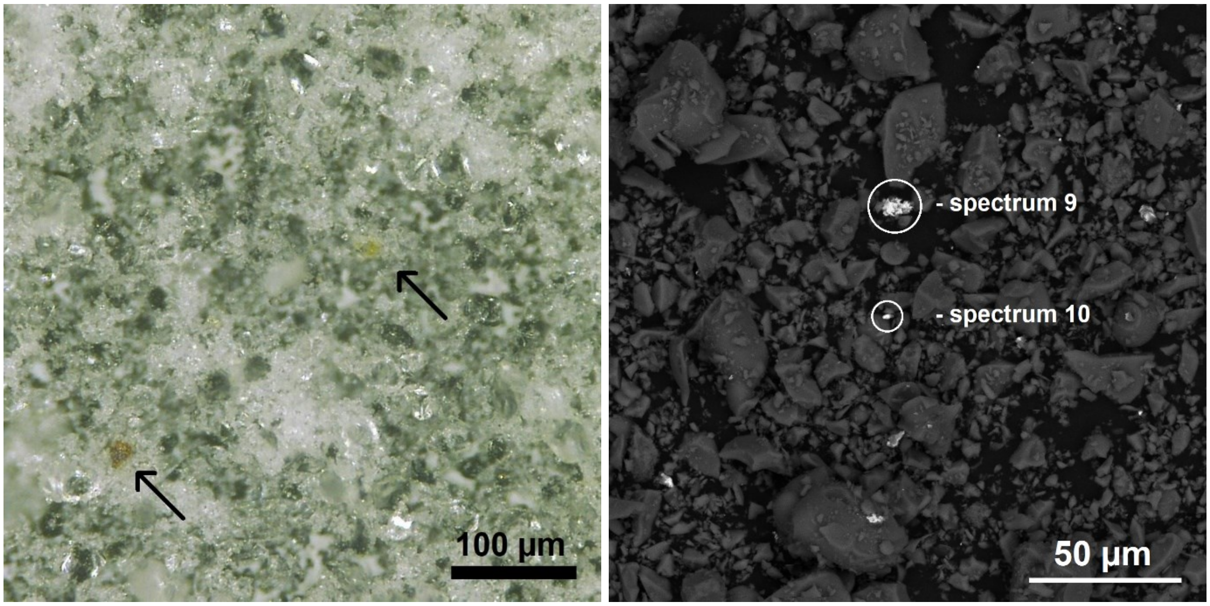

Figure S 66: Microscopic (left) and SEM image with backscattered electron detection (right) of the flow reactor's drilled-out glass frit after several photocatalytic experiments and consecutive cleaning steps. The annotations refer to the EDX spectra summed up in Table S 5 whereas the arrows highlight potential contaminations.

Table S 5: Summary of EDX spectra measured on the flow reactor’s drilled-out glass frit after several photocatalytic experiments and consecutive cleaning steps. Values given in at%.

Element	Spectrum 1	Spectrum 2	Spectrum 3	Spectrum 4	Spectrum 5	Spectrum 6	Spectrum 7	Spectrum 8	Spectrum 9	Spectrum 10
O	56.55	40.29	43.61	27.18	78.67	67.48	31.78	37.37	47.81	78.13
F	1.73									
Na	2.53	2.21	3.09		0.56	2.39	1.01	0.93	1.97	1.43
Al	0.90	1.04	2.91	0.27		0.91	0.78	0.95	1.10	1.43
Si	24.49	26.06	14.09	6.15	4.83	28.90	11.30	10.42	14.91	10.19
S									0.24	
Cl			0.09							
K	0.23	0.23	0.10			0.32				0.10
Ca			0.09							
Ti			0.14							
Cr			6.66							
Mn			0.65							
Fe			23.90							
Ni			3.47							
Cu	12.27	28.65	0.70	58.09	0.52		50.52	43.73	30.46	0.33
Sr					1.68					
Mo			0.50							
Ru					0.66					
Ag	0.24	0.22		2.09			0.70	1.71	0.58	
Sn	1.06	1.30		6.21			3.91	4.89	2.92	
W					13.08					8.39
Total	100.00	100.00	100.00	100.00	100.00	100.00	100.00	100.00	100.00	100.00
Mater.	**bronze**	**bronze**	**steel**	**bronze**	**WO_3**	**bare frit**	**bronze**	**bronze**	**bronze**	**WO_3**

Mean for spectra 1,2,4,7,8,9: 90.2 at% Cu, 2.0 at% Ag, 7.8 at% Sn. These values suggest that Ag stems from a bronze rather than $AgNO_3$. Bronze and steel are assumed to stem from the mechanical removal of the glass frit’s top layer.

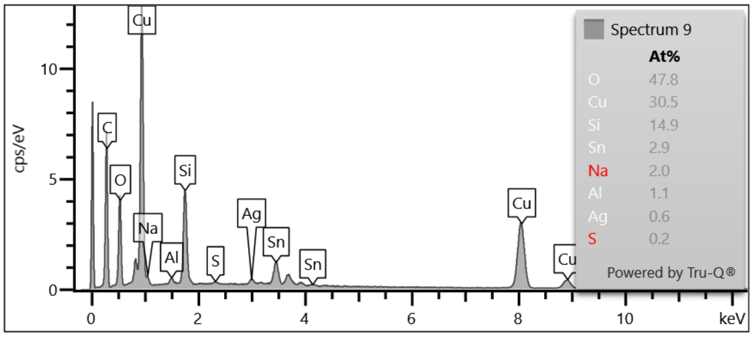

Figure S 67: EDX spectrum of a contamination on the flow reactor’s glass frit after several photocatalytic experiments and consecutive cleaning steps. Spectrum measured at position “9” marked in Figure S 66.

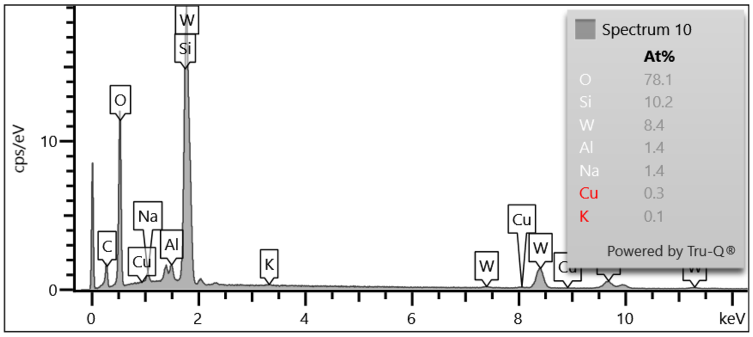

Figure S 68: EDX spectrum of a contamination on the flow reactor’s glass frit after several photocatalytic experiments and consecutive cleaning steps. Spectrum measured at position “10” marked in Figure S 66.

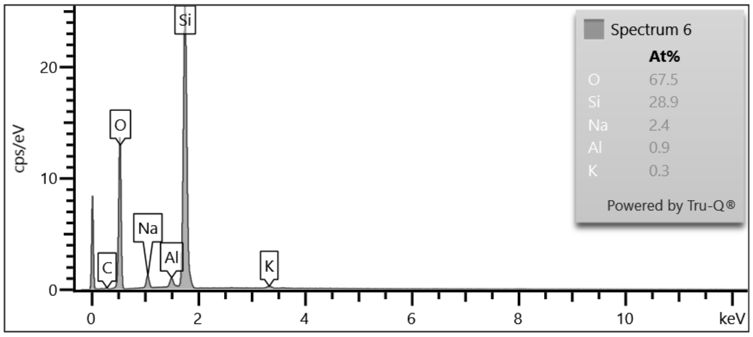

Figure S 69: EDX spectrum of non-contaminated parts of the flow reactor’s glass frit after several photocatalytic experiments and consecutive cleaning steps.

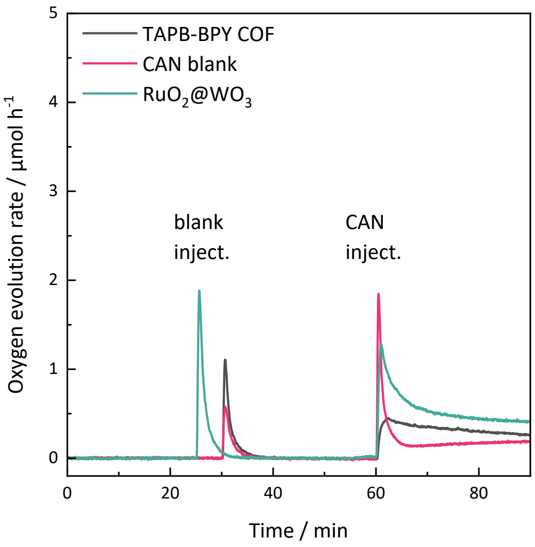

Figure S 70: Control experiment for the chemical water oxidation with CAN as oxidant (78mM) and ruthenium-loaded tungsten oxide as WOC.

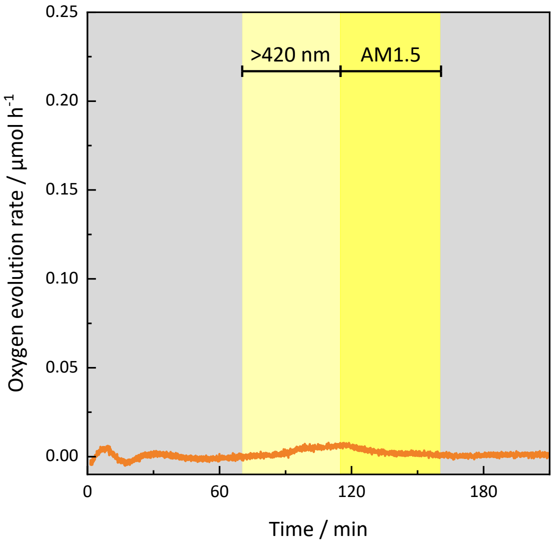

Figure S 71: Photocatalytic oxygen evolution experiment with Ir@TAPB-BPY COF (1.9 wt% Ir) in a pristine flow reactor. Reaction condition: (5 mg COF, 10 mM $AgNO_3$, 5 mL). Illumination with 1 sun (AAA sun simulator) and optical filters as specified. Grey areas represent dark reaction conditions.

S8 - Electrochemistry and Computational Chemistry

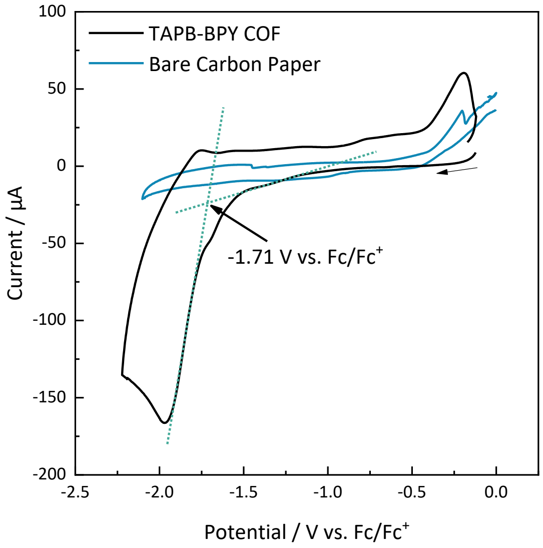

Figure S 72: Cyclic voltammogram for TAPB-BPY COF. COF measured after deposition on carbon paper working electrodes.

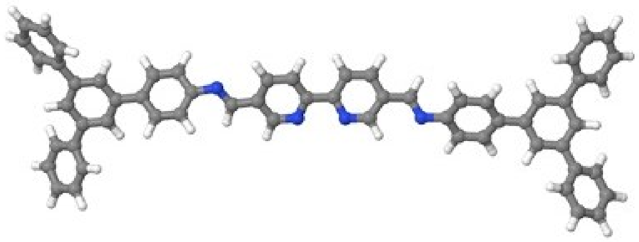

Figure S 73: Cluster model used to calculate IP and EA of TAPB-BPY COF.

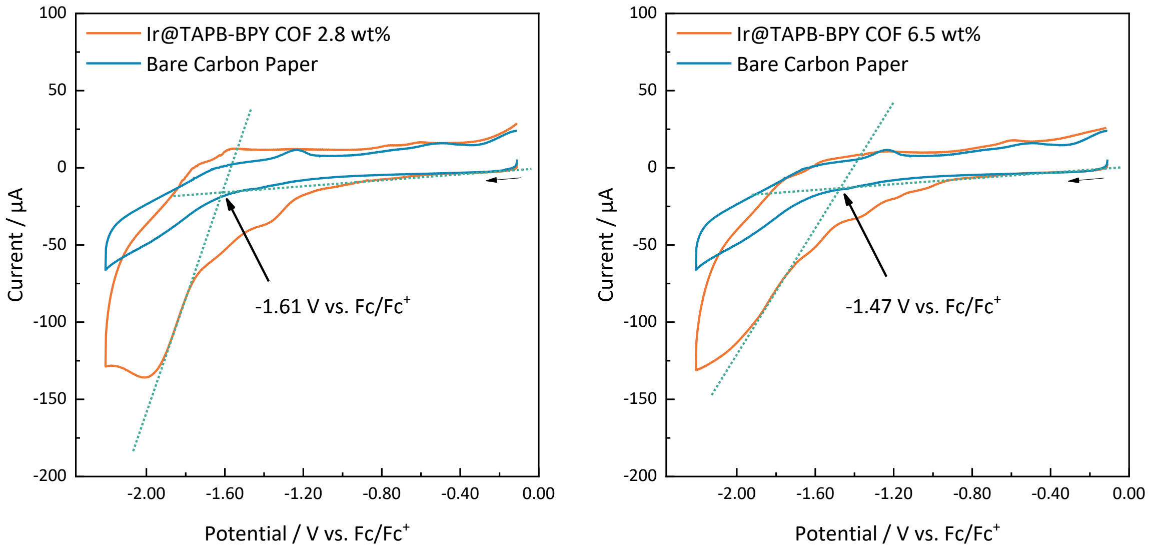

Figure S 74: Cyclic voltammograms for Ir@TAPB-BPY COF samples of varying iridium contents. COFs measured after deposition on carbon paper working electrodes.

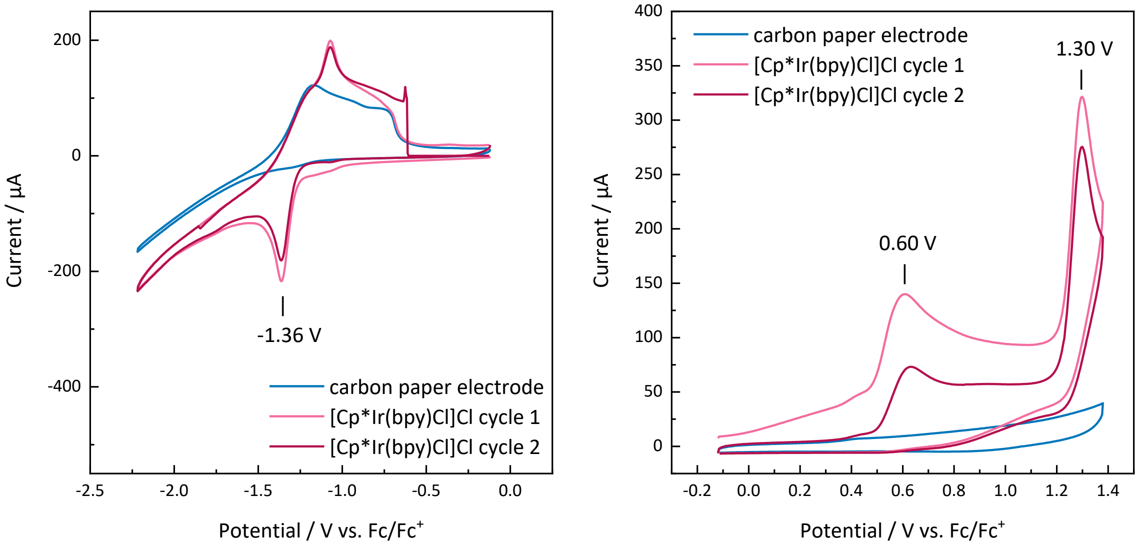

Figure S 75: Cyclic voltammograms of molecular [Cp*Ir(bpy)Cl]Cl dissolved in anhydrous acetonitrile (0.1 M NBu_4PF_6). Annotation at -1.36 V, 0.60 V, and 1.30 V vs. Fc/Fc^+ mark features ascribed to Ir^{III} reduction,[70] Cl oxidation,[71] and Ir^{III} oxidation,[71] respectively.

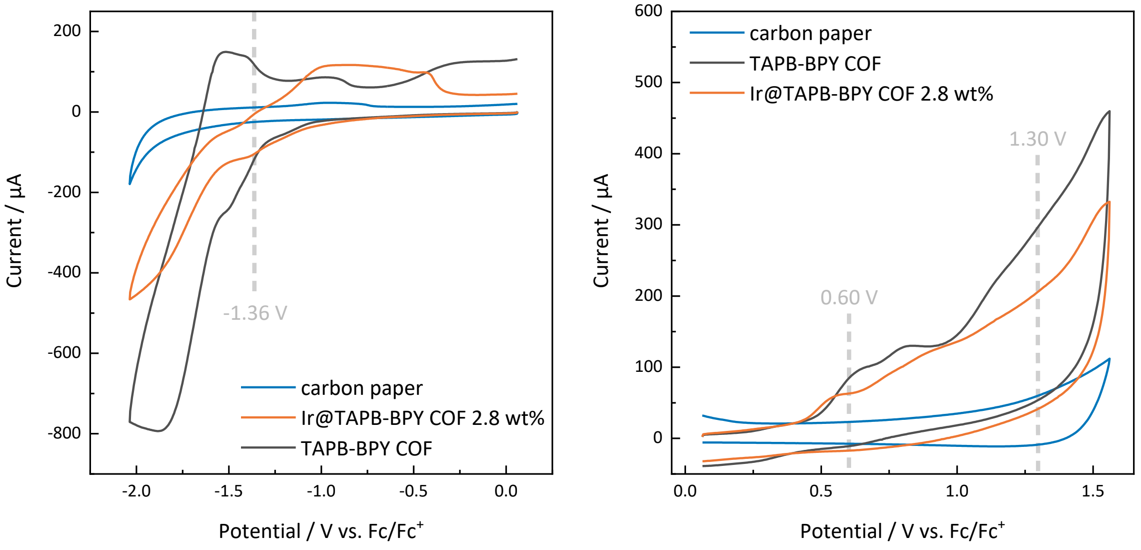

Figure S 76: Cyclic voltammograms for TAPB-BPY COF and Ir@TAPB-BPY COF in anhydrous acetonitrile (0.1 M NBu_4PF_6). COFs deposited on carbon paper working electrodes. Annotations correspond to redox features expected for [Cp*Ir(bpy)Cl]Cl moieties as depicted in Figure S 75.

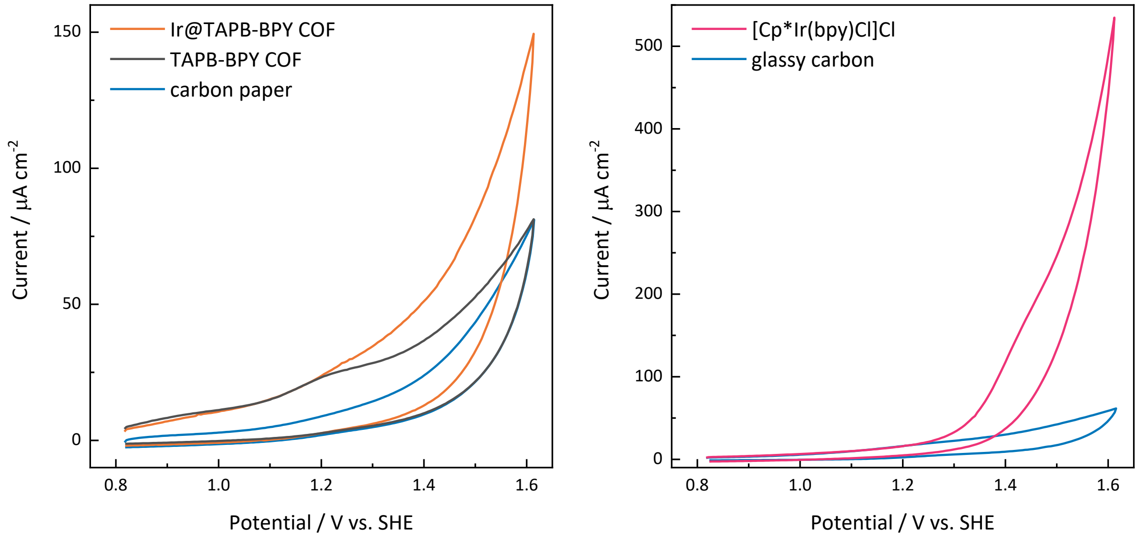

Figure S 77: Cyclic voltammograms for Ir@TAPB-BPY COF (left) and [Cp*Ir(bpy)Cl]Cl (right, 1.13 mM) in aqueous electrolyte (0.5 M sodium phosphate buffer, pH 7). Measurements conducted against an RHE reference electrode and converted to SHE according to the Nernst equation for better comparability. Note that the irreversible oxidative features for [Cp*Ir(bpy)Cl]Cl are usually not ascribed to actual water oxidation catalysis.[72–74]

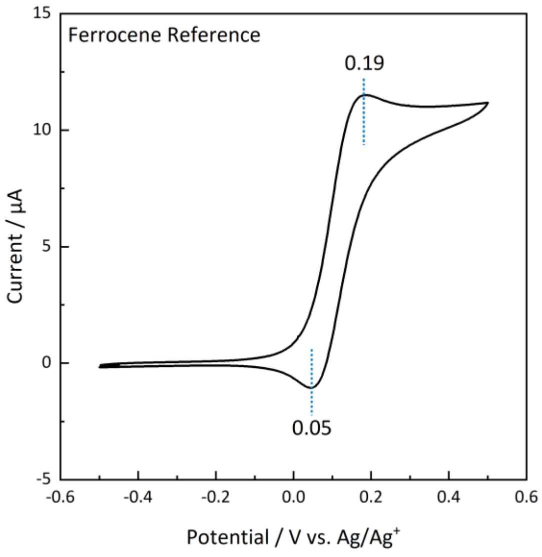

Figure S 78: Exemplary cyclic voltammogram for a ferrocene reference. For each series of CV measurements, a new reference was measured.

Table S 6: Comparison of band positions and related data for TAPB-BPY COF and Ir@TAPB-BPY COF.

**COF**	**$E_{red, onset}$**	**E_{CB}**	**Opt. band gap**	**E_{VB}**	**Source**
TAPB-BPY COF	-1.59 V vs. Ag/Ag^+<br>-1.71 V vs. Fc/Fc^+	-3.39 eV	2.50 eV	-5.89 eV	This work
TAPB-BPY COF („Bp-COF“)	-1.27 V vs. Ag/Ag^+<br>-1.34 V vs. Fc/Fc^+	-3.76 eV	2.41 eV	-6.17 eV	Ref. [[28]] [a]
Ir@TAPB-BPY COF (2.8 wt% Ir)	-1.50 V vs. Ag/Ag^+<br>-1.61 V vs. Fc/Fc^+	-3.49 eV	2.40 eV	-5.89 eV	This work
Ir@TAPB-BPY COF (6.5 wt% Ir)	-1.36 V vs. Ag/Ag^+<br>-1.47 V vs. Fc/Fc^+	-3.63 eV	2.25 eV	-5.89 eV	This work

Conversion of potentials done according to E(V *vs.* vac) = -(E(V *vs.* Fc/Fc+) + 5.1) eV [a]: $E_{1/2}(Fc/Fc^+)$ = 0.07 vs. Ag/Ag^+ extracted from reference measurement in the supporting information.

Table S 7: Ionisation potential (IP) and electron affinity (EA) values predicted for cluster models of TAPB-BPY COF and Ir@TAPB-BPY COF in water and acetonitrile. All values are given in V vs. SHE.

	In acetonitrile		In water	
	IP	EA	IP	EA
TAPB-BPY COF	1.16	-1.57	1.13	-1.54
Ir@TAPB-BPY COF (Cp*Ir-H_2O fragment)	1.42		1.38	
Ir@TAPB-BPY COF (Cp*Ir-MeCN fragment)	1.41			

The representative COF cluster used for the prediction of TAPB-BPY COF is depicted in Figure S 73. Detailed structural data for the Ir@TAPB-BPY COF cluster can be found in the .zip file provided alongside the manuscript.

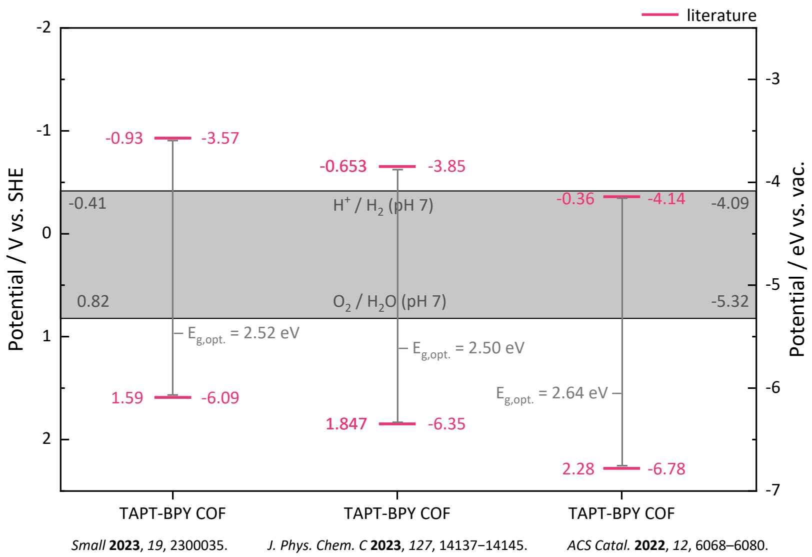

Figure S 79: Band positions for TAPT-BPY COF retrieved from literature references given in the annotations.

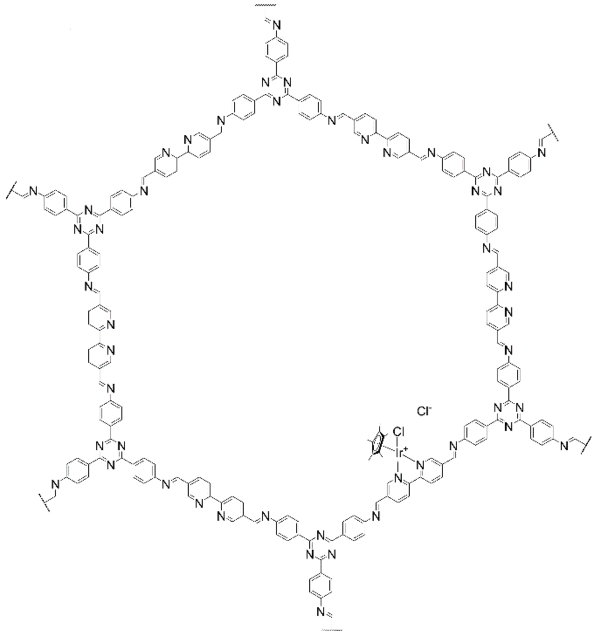

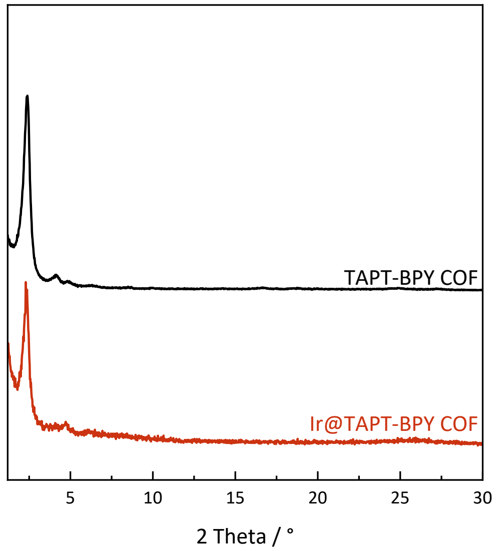

Figure S 80: Molecular structure (left) and XRPD patterns (right) of Ir@TAPT-BPY COF.

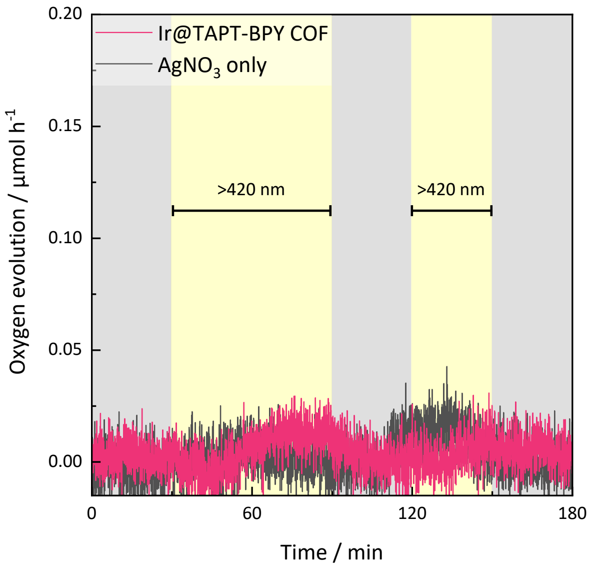

Figure S 81: Photocatalysis experiment with Ir@TAPT-BPY COF. Reaction conditions: 5 mg COF (2.3 wt% Ir), $AgNO_3$ (10 mM, 5 mL), 300 W Xe lamp, optical filter as annotated.

Table S 8: ^{1}H-NMR spectroscopy results for the attempted photo-oxidation of methanol to formaldehyde (signal at 9.6 ppm attributed to aldehyde protons).

**Catalyst**	**Aldehyde signal (9.6 ppm)**
Ir@TAPB-BPY COF	n.d.
TAPB-BPY COF	n.d.
[Cp*Ir(bpy)Cl]Cl	n.d.

The absence of signals attributable to formaldehyde hints to an unsuccessful photo-oxidation of methanol in either case.

S9 - Reflections on the GCC Concept

SURENDRANATH and co-workers found that after binding rhodium, ruthenium, or rhenium complexes to graphitic carbon *via* terminal phenazine sites the electrocatalytic activity and the underlying mechanisms drastically changed compared to the molecular analogues.[75–77] In a nutshell, the catalysis – in their case, CO_2 reduction and hydrogen evolution – proceeded without the metal atom changing its oxidation state, as opposed to similar molecular catalysts in solution.[75,78] We note that in our case iridium is bound to an extended π-system via bipyridine sites resembling phenazine, and that during catalysis charges could be stored on the COF instead of the bound metal atom, thus hampering oxidation of metal-bound water molecules. A recent review discusses this concept explicitly for COFs in the context of electrocatalytic CO_2 reduction.[79]

Based on the assumption that photocatalytic water oxidation with Ir@TAPB-BPY COF involves a mechanism where the metal oxidation state does not change (following the GCC approach), we use DFT to calculate the free energy profile of water oxidation at a redox-innocent iridium complex bound to an exemplary TAPB-BPY COF fragment. We note that the four electron transfers underlying water oxidation differ significantly in their respective free energy changes (Scheme S 1:, Figure S 83). TAPB-BPY COF might especially be struggling to drive step (2) with photogenerated holes (ΔG^0 = 1.30 eV, E_{VB} = 1.16/1.39 V vs. SHE, Figure 4). By considering only proton-coupled electron transfers (PCETs) eventual transition barriers associated with either e^- or H^+ transfer are neglected, so our ΔG^0 values can be regarded lower estimates.[80,81] Chemical water oxidation with CAN (vide supra), however, can seemingly overcome the supposedly limiting kinetics of the individual oxidation events in Ir@TAPB-BPY COF due to its high potential of ca. 1.75 V at pH 1.[42] The free energy change for step (2) at pH 0 was calculated to be 1.71 eV (Scheme S 2).

ΔG^0 at pH 7

	(1)		(2)		(3)		(4)	
	1.16 eV		1.30 eV		0.60 eV		0.03 eV	
Ir(III)-H_2O	⟶	Ir(III)-$OH^•$	⟶	Ir(III)-O(H)OH	⟶	Ir(III)-OOH	⟶	Ir(III)-H_2O
	- e^-		- e^-		- e^-		- e^-	
	- H^+		- H^+		- H^+		- H^+	
			+ H_2O				- O_2	

Scheme S 1: Schematic reaction pathway for water oxidation on Ir@TAPB-BPY COF assuming redox-innocent Ir(III) centers according to the GCC principle. Changes in free energy given in eV at pH 7.

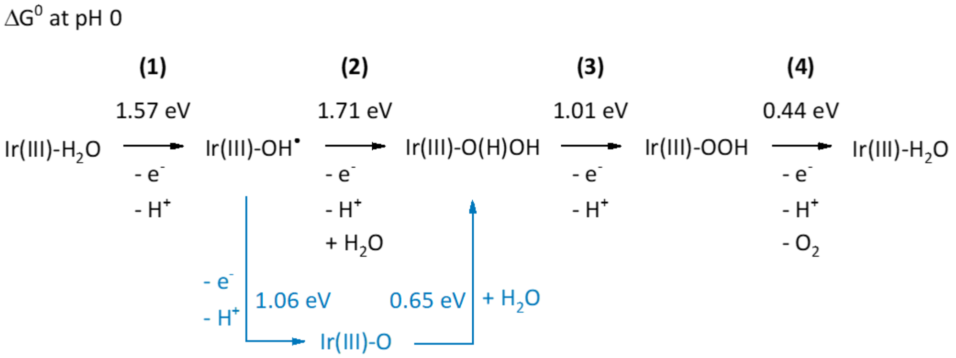

Scheme S 2: Schematic reaction pathway for water oxidation on Ir@TAPB-BPY COF assuming redox-innocent Ir(III) centers according to the GCC principle. Changes in free energy given in eV at pH 0. Apart from PCET steps, the Ir(III)-O species (blue) is given for better comparison with Ir(V)=O species which are an important intermediate in the WNA mechanism of molecular Ir WOCs.

For Co@TAPB-BPY COF – which was reported to evolve oxygen photocatalytically –, the predicted free energy changes for water oxidation indicate even stronger kinetic limitations.[28] Here, oxidation of COF-bound Co-OH species and subsequent formation of the O-O bond is associated with ΔG^0 = 1.62 eV at pH 7 (Figure S 84) which would be achievable given literature values for E_{VB} of 1.67 V vs. NHE, but not with our experimentally determined and calculated values of 1.39 and 1.16 V vs. SHE, respectively (Figure 4).

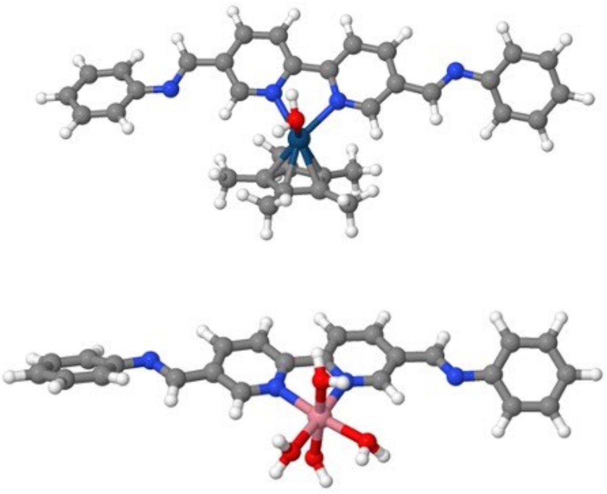

Figure S 82: Cluster model of the Ir@TAPB-BPY COF (top) and Co@TAPB-BPY COF (bottom) used when calculating the free energy landscape of the water oxidation mechanism.

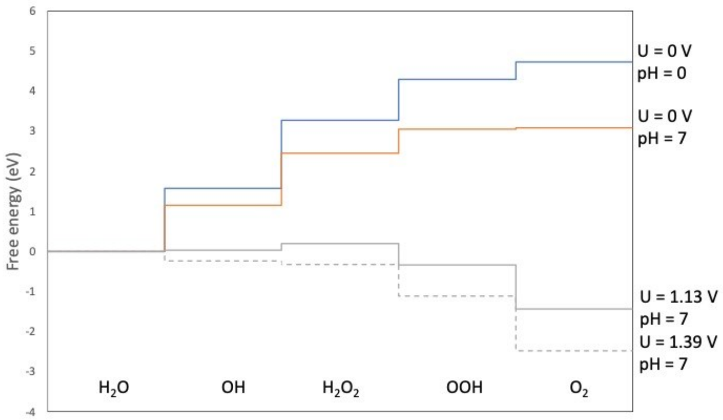

Figure S 83: Predicted free energy diagram for the oxidation of water for Ir@TAPB-BPY COF for different pH and applied potential values.

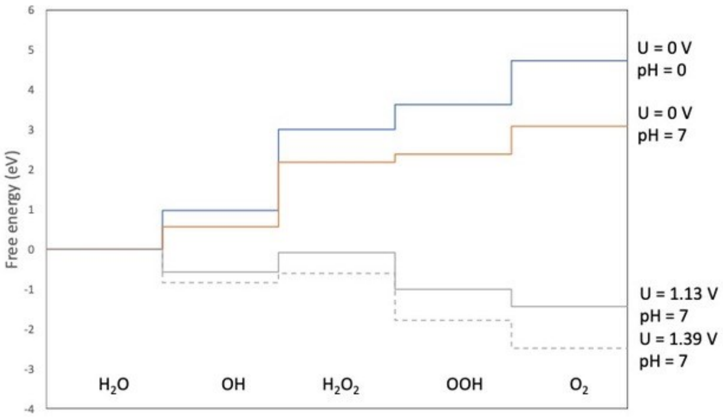

Figure S 84: Predicted dree energy diagram for the oxidation of water for Co@TAPB-BPY COF for different pH and applied potential values.

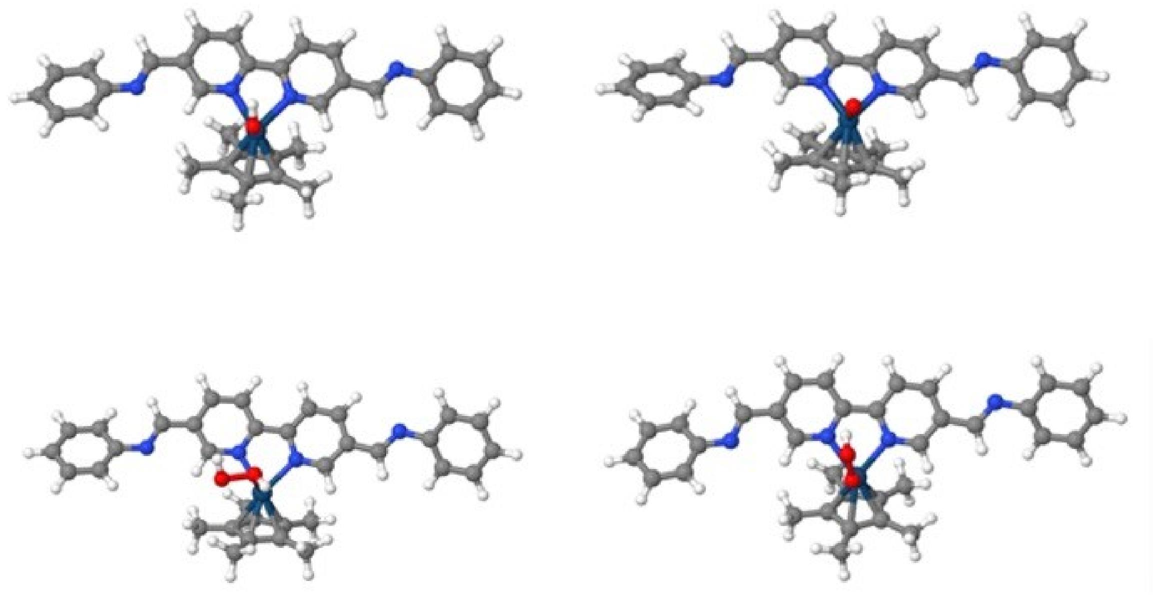

Figure S 85: Minimum energy structures for the Ir@TAPB-BPY COF cluster model with an OH group (top, left), an O atom (top, right), adsorbed H_2O_2 (bottom, left) and an OOH group (bottom, right).

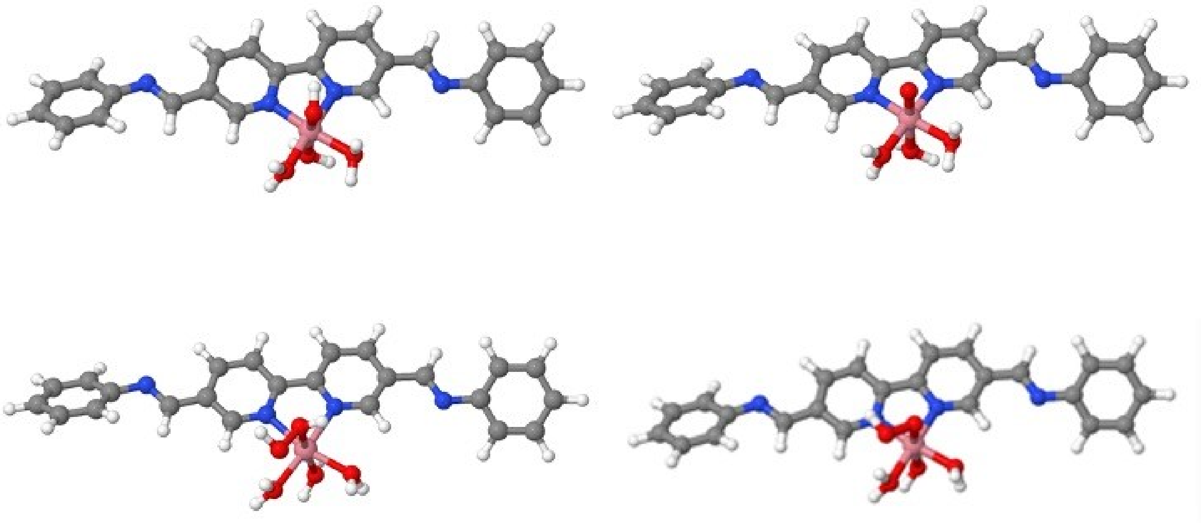

Figure S 86: Minimum energy structures for the Co@TAPB-BPY COF cluster model with an OH group (top, left), an O atom (top, right), adsorbed H_2O_2 (bottom, left) and an OOH group (bottom, right).

S10 - Photocatalysis Reproduction

Unfortunately, comparable blank experiments as discussed in section S7 have not been conducted in other literature reports on COF photocatalysts and are only rarely ever mentioned (Table S 10). We thus tried to reproduce some of the few examples for oxygen evolution with COF photocatalysts, namely "BpCo-COF-1" as well as cobalt-loaded "I-TST", which we will refer to as Co@TAPB-BPY COF and Co@TTI-COF in the following (Figure S 87).[27,28,82] Both have been reported for OER in conjunction with $AgNO_3$ as SEA, and can be prepared from commercially available building blocks. Material characterization with respect to crystallinity, porosity, and metal content shows conformity between our reproduced COFs and the respective literature data (Figure S 89 - Figure S 94). For Co@TAPB-BPY COF, we found cobalt evenly distributed over the spherical particles, in line with the proposed binding of Co^{2+} to the frameworks' bipyridine site (Figure S 95, Figure S 96). In contrast, for Co@TTI-COF SEM and TEM imaging revealed heterogenous deposition of amorphous CoO_xH_y species (Figure S 97, Figure S 98), which was not mentioned in the original publication.[27] In a separate study, we identify cobalt oxide deposits as the true active species in the electrocatalytic oxygen evolution reaction with a cobalt-loaded COF, which highlights the importance of post-catalytic catalyst characterization.[83]

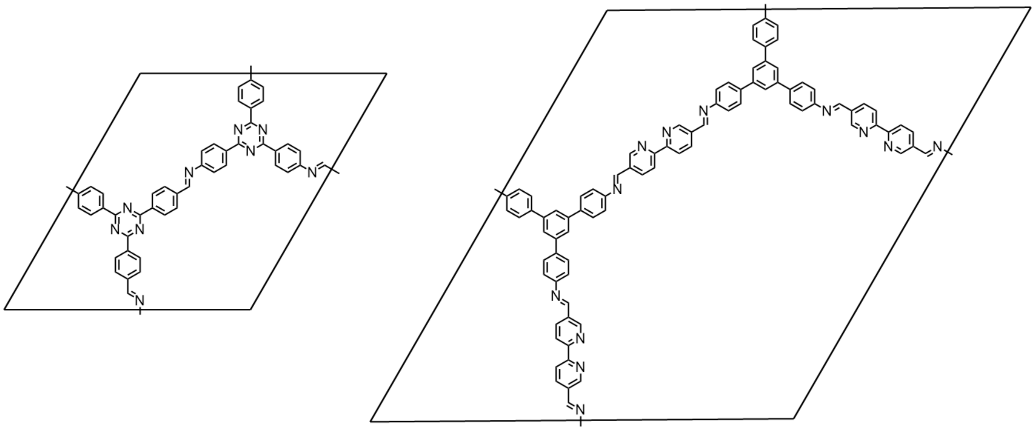

Figure S 87: Unit cells as schematic representation of TTI-COF (left) and TAPB-BPY COF (right).

However, when assessing the photocatalytic activity of Co@TAPB-BPY and Co@TTI-COF, we could not confirm the reported oxygen evolution rates. In fact, no significant amounts of evolved oxygen could be detected at all, even though we meticulously followed the given protocol (Figure S 88). Attempts to achieve distinct oxygen evolution by the COFs through variation of the reaction parameters were unsuccessful (Table S 11, Table S 12). Only when using contaminated glassware, we could obtain apparent gravimetric OER rates in the range of 8 –200 µmol g^{-1} h^{-1}, which coincidentally match the literature activity of Co@TAPB-BPY and Co@TTI-COF of 152 and 37 µmol g^{-1} h^{-1}, respectively (Figure S 100 - Figure S 102). As a third example, we could also not reproduce the recently reported photocatalytic oxygen evolution over cobalt-loaded TAPT-BPY COF (Figure S 105-Figure S 107).[29]

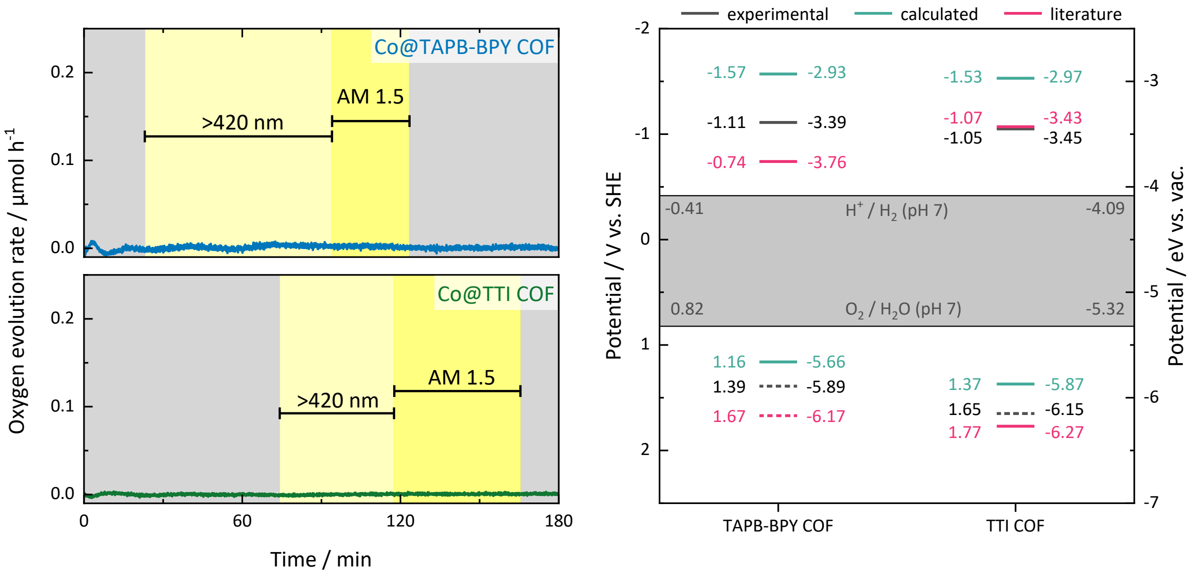

Figure S 88: Reproduction of photocatalytic oxygen evolution experiments with literature-known COFs. Reaction conditions: 2.0 mg Co@TAPB-BPY COF (1.0 wt% Co), $AgNO_3$ (5 mM, 20 mL), bulk reactor (top) or: 1.0 mg Co@TTI-COF (0.9 wt% Co), $AgNO_3$ (10 mM, 5 mL), 10 mg La_2O_3, flow reactor (bottom). Both experiments were performed with an AAA solar simulator at 1 sun and with optical filters as annotated. Right: Comparison of experimentally determined band positions (*via* CV in MeCN; black), computationally predicted ionization potentials and electron affinities (in MeCN, turquoise), and literature values (pink) for TAPB-BPY COF and TTI COF. Dashed lines represent values obtained indirectly, for example E_{VB}/VBM calculated from the E_{CB}/CBM *via* subtraction of the optical band gap. Potentials for the water splitting half reactions illustrated as grey area. See Table S 7 and Table S 13 for details.

Table S 9: Comparison of COFs and other systems reported for photocatalytic water oxidation.

**Photosensitizer**	**Cocatalyst**	**SEA, buffer**	**OER activity**	**Opt. filter**	**Ref.**
BpCo-COF-0	None	5 mM $AgNO_3$	traces	>420 nm	28
BpCo-COF-1	Co 1.2 wt%	5 mM $AgNO_3$	152 µmol g^{-1} h^{-1}	>420 nm	28
g-$C_{40}N_3$-COF	None	10 mM $AgNO_3$, La_2O_3	1.6 µmol h^{-1}<br>32 µmol g^{-1} h^{-1}	>420 nm	84
g-$C_{40}N_3$-COF	Co 3.0 wt%	10 mM $AgNO_3$, La_2O_3	2.5 µmol h^{-1}<br>50 µmol g^{-1} h^{-1}	>420 nm	84
I-TST = TTI-COF	None	10 mM $AgNO_3$, La_2O_3	0.13 µmol h^{-1}<br>13 µmol g^{-1} h^{-1}	>420 nm	27, a
I-TST = TTI-COF	$Co(OH)_2$	10 mM $AgNO_3$, La_2O_3	~0.4 µmol h^{-1}<br>~37 µmol g^{-1} h^{-1}	>420 nm	27, a
sp^2c-COF	$Co(NO_3)_2$	10 mM $AgNO_3$, La_2O_3	1.1 µmol h^{-1}<br>22 µmol g^{-1} h^{-1}	>420 nm	85

g-$C_{52}N_6$-COF	$Co(NO_3)_2$ 3wt%	10 mM $AgNO_3$, La_2O_3	12.5 µmol g^{-1} h^{-1}	>420 nm	[86]
g-$C_{54}N_6$-COF	$Co(NO_3)_2$ 3wt%	10 mM $AgNO_3$, La_2O_3	51 µmol g^{-1} h^{-1}	>420 nm	[86]
N_0-COF	"$CoNO_3$"	10 mM $AgNO_3$, La_2O_3	390 µmol g^{-1} h^{-1}	>300 nm	[87]
TAPT-Bpy-COF	None	10 mM $AgNO_3$, La_2O_3	2.98 µmol h^{-1} 298 µmol g^{-1} h^{-1}	>420 nm	[29, a]
TAPT-Bpy-COF-Co-3	Co 2.12 wt%	10 mM $AgNO_3$, La_2O_3	4.83 µmol h^{-1} 483 µmol g^{-1} h^{-1}	>420 nm	[29]
TpBpy-NS	Pt ~1.2 wt%	10 mM $AgNO_3$, La_2O_3	3.18 µmol h^{-1} 212 µmol g^{-1} h^{-1}	>420 nm	[88]
TpBpy-2-NS	Pt ~1.2 wt%	10 mM $AgNO_3$, La_2O_3	1.36 µmol h^{-1} 91 µmol g^{-1} h^{-1}	>420 nm	[88]
BtB-COF	16 wt% $Co(ClO_4)_2$	0.1 mM $AgNO_3$ [b]	6.65 µmol h^{-1} 665 µmol g^{-1} h^{-1}	>420 nm	[89]
Ni-TAPP-COF [c]		50 mM $AgNO_3$	0.62 µmol h^{-1} 123 µmol g^{-1} h^{-1}	AM1.5	[90]
Ni-TAPP-COF-BF_2 [c]		50 mM $AgNO_3$	7.02 µmol h^{-1} 1404 µmol g^{-1} h^{-1}	AM1.5	[90]
Zn-TAPP-COF-BF_2 [c]		50 mM $AgNO_3$	2.48 µmol h^{-1} 496 µmol g^{-1} h^{-1}	AM1.5	[90]
Co-TAPP-COF-BF_2 [c]		50 mM $AgNO_3$	2.24 µmol h^{-1} 448µmol g^{-1} h^{-1}	AM1.5	[90]
Mn-TAPP-COF-BF_2 [c]		50 mM $AgNO_3$	1.29 µmol h^{-1} 257 µmol g^{-1} h^{-1}	AM1.5	[90]
CoTPP-$CoBpy_3$ [c]		5 mM $AgNO_3$	36.6 µmol h^{-1} 7323 µmol g^{-1} h^{-1}	AM1.5	[91]
CoTPP-Bpy [c]		5 mM $AgNO_3$	1.5 µmol h^{-1} 300 µmol g^{-1} h^{-1}	AM1.5	[91]

Co-TFPP-COF<br>Co-TPFO-COF [c,d,e]		50 mM $AgNO_3$	12.0 µmol h^{-1}<br>2399 µmol g^{-1} h^{-1}	>420 nm	92
TpBPy	None	25 mM $AgNO_3$	Evolves N_2 instead	>400 nm	93
$CoCl_2$-TpBPy	1.9 wt% Co	25 mM $AgNO_3$	0.01 µmol h^{-1}<br>1 mmol g^{-1} h^{-1}	>400 nm	93
Co_3O_4/Re/COF	3 wt% Co_3O_4<br>2 wt% Re	0.1 mM $AgNO_3$	4.0 µmol h-1<br>400 µmol g-1 h-1	>420 nm	94
CoTPP-DMTP-COF	None	0.1 M $AgNO_3$	0.2 µmol h-1<br>42.5 µmol g-1 h-1	>420 nm	95
Bpy→CoTPP<br>-DMTP-COF	None	0.1 M $AgNO_3$	1.4 µmol h^{-1}<br>280 µmol g^{-1} h^{-1}	>420 nm	95
Bpy→CoTPP<br>-DMTP-COF	6 wt% Co^{2+}<br>$Co(NO_3)_2$	0.1 M $AgNO_3$	5.775 µmol h^{-1}<br>1155 µmol g^{-1} h^{-1}	>420 nm	95
CTF-BPDCN	None	10 mM $NaIO_3$<br>La_2O_3	0.78 µmol h^{-1}<br>26 µmol g^{-1} h^{-1}	>420 nm	96
CTF-1	RuO_2	200 mM $AgNO_3$	7 µmol h^{-1}<br>140 µmol g^{-1} h^{-1}	>420 nm	97
CTF-1	None	50 mM $AgNO_3$	1.5 µmol h^{-1}<br>30 µmol g^{-1} h^{-1}	>420 nm	97
P10 – lin. polymer	1 wt% Co	10 mM $AgNO_3$<br>200 mg La_2O_3<br>100 mL	16.6 µmol h^{-1}<br>332 µmol g^{-1} h^{-1}	full arc	98
			5.2 µmol h^{-1}<br>104 µmol g^{-1} h^{-1}	>420 nm	98

[a] OER values extracted from graph. [b] The authors state [$AgNO_3$] as 0.1 mM and 1 mM at different sections of the respective supporting information. [c] For the porphyrin-based COFs discussed here, the conceptualization of photosensitizer and cocatalyst varies from the other examples. [d] The authors interchangeably use two different denominations for their COF. [e] The authors also report the use of “$Na_2S_2O_4$” and “$NaIO_4$” as alternative sacrificial electron acceptors, supposedly meaning the common SEAs $Na_2S_2O_8$ and $NaIO_3$. To avoid misinterpretation, we refrain from including the respective results in this table.

Table S 10: Comparison of background oxygen evolution measurements (sacrificial only) in selected publications.

SEA	conc., vol.	buffer/additive	OER	Illumination	Ref.	Material
$AgNO_3$	10 mM, 100 mL	200 mg La_2O_3	not measured	300 W Xe lamp, >420 nm	85	sp^{2}c-COF
$AgNO_3$	5 mM, 100 mL	none	unclear "blank"	300 W Xe lamp, >420 nm	28	Bp-COF
$AgNO_3$	10 mM, 100 mL	200 mg La_2O_3	"Oxygen was not detected"	300 W Xe lamp, >420 nm	29	TAPT-Bpy-COF
$AgNO_3$	10 mM, 100 mL	200 mg La_2O_3	not measured	300 W Xe lamp, >420 nm	84	g-C40N3-COF
$AgNO_3$	10 mM, 50 mL	100 mg La_2O_3	not measured	300 W Xe lamp, >420 nm	27	I-TST *a.k.a.* TTI-COF
$AgNO_3$	10 mM, 100 mL	200 mg La_2O_3	"no O_2 evolution detected"	300 W Xe lamp, >420 nm	86	g-C52N6-COF<br>g-C54N6-COF
$AgNO_3$	10 mM, 100 mL	200 mg La_2O_3	not measured	unknown, >300 nm	87	N_0-COF
$AgNO_3$	10 mM, 50 mL	100 mg La_2O_3	not measured	300 W Xe lamp, >420 nm	88	TpBpy-NS
$AgNO_3$	0.1 mM[a], 50 mL	none	not measured	300 W Xe lamp, >420 nm	89	BtB-COF
$NaIO_3$	unknown	none	not measured	300 W Xe lamp, >420 nm	89	BtB-COF
NaS_2O_8 (sic)	unknown	none	not measured	300 W Xe lamp, >420 nm	89	BtB-COF
$AgNO_3$	5 mM, 100 ml	none	not detected	300 W Xe lamp, AM1.5G	91	CoTPP-$CoBpy_3$
$AgNO_3$	25 mM, 200 mL	none	not measured	300 W Xe lamp, >400 nm	93	$CoCl_2$-TpBPy
$K_2S_2O_8$	unknown	none	not measured	300 W Xe lamp, >400 nm	93	$CoCl_2$-TpBPy
$NaIO_3$	unknown	none	not measured	300 W Xe lamp, >400 nm	93	$CoCl_2$-TpBPy
$AgNO_3$	0.1 mM, 50 mL	none	not measured	300 W Xe lamp, >420 nm	94	Co_3O_4/Re/COF
$AgNO_3$	0.1 M, 100 mL	200 mg La_2O_3	no O_2 detected	300 W, >420 nm	95	Bpy→CoTPP-DMTP-COF

$AgNO_3$	10 mM, 100 mL	200 mg La_2O_3	0 µmol h^{-1}	300 W, >420 nm	98	P10
$AgNO_3$	10 mM, 100 mL	none	ca. 3 µmol in 5 h	300 W, UV+vis	99	IrO_2

[a] The authors state 0.1 mM and 1 mM at different sections of the respective supporting information.

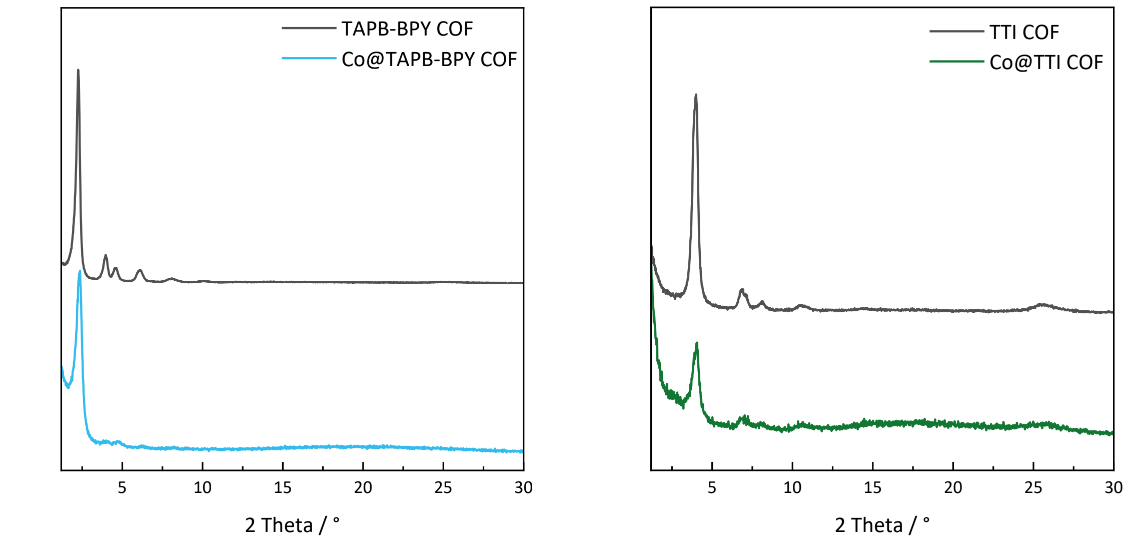

Figure S 89: XRPD patterns for reproduced COFs Co@TAPB-BPY COF (left) and Co@TTI-COF (right) with the respective unmetalled COF. We note significant deviations between our diffractogram and that presented in the literature for TTI-COF.[27]

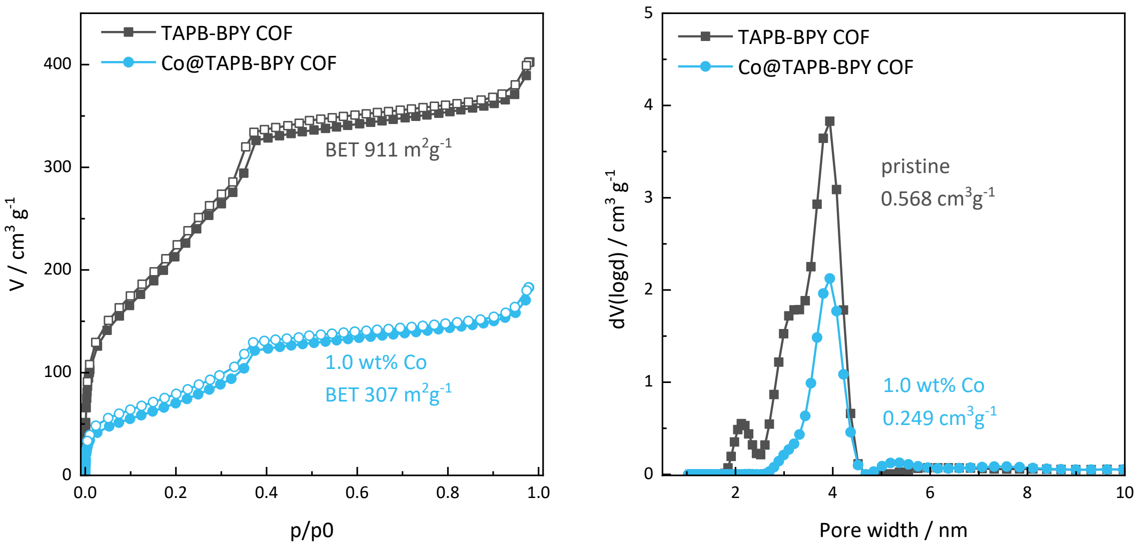

Figure S 90: Nitrogen sorption isotherms at 77 K (left) and pore size distribution (right) for TAPB-BPY COF before and after postsynthetic loading with $Co(NO_3)_2$. Filled and open symbols represent the adsorption and the desorption branches, respectively. The pore size distribution was obtained from a QSDFT kernel for cylindrical pores (adsorption branch). The cobalt content was determined via ICP-OES.

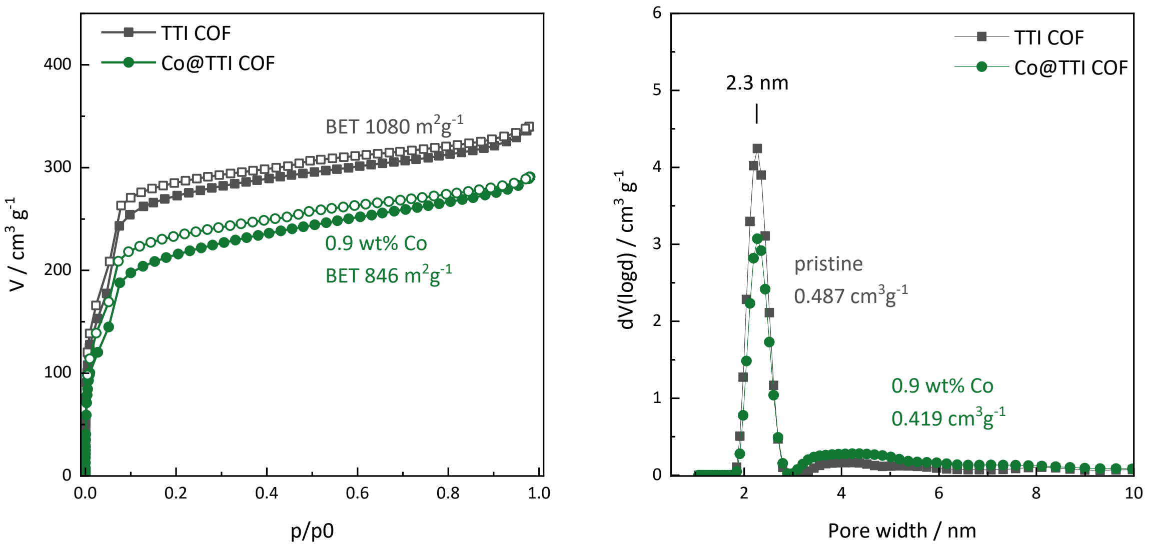

Figure S 91: Nitrogen sorption isotherms at 77 K (left) and pore size distribution (right) for TTI-COF before and after postsynthetic loading with $Co(NO_3)_2$. Filled and open symbols represent the adsorption and the desorption branches, respectively. The pore size distribution was obtained from a QSDFT kernel for cylindrical pores (adsorption branch). The cobalt content was determined via ICP-OES.

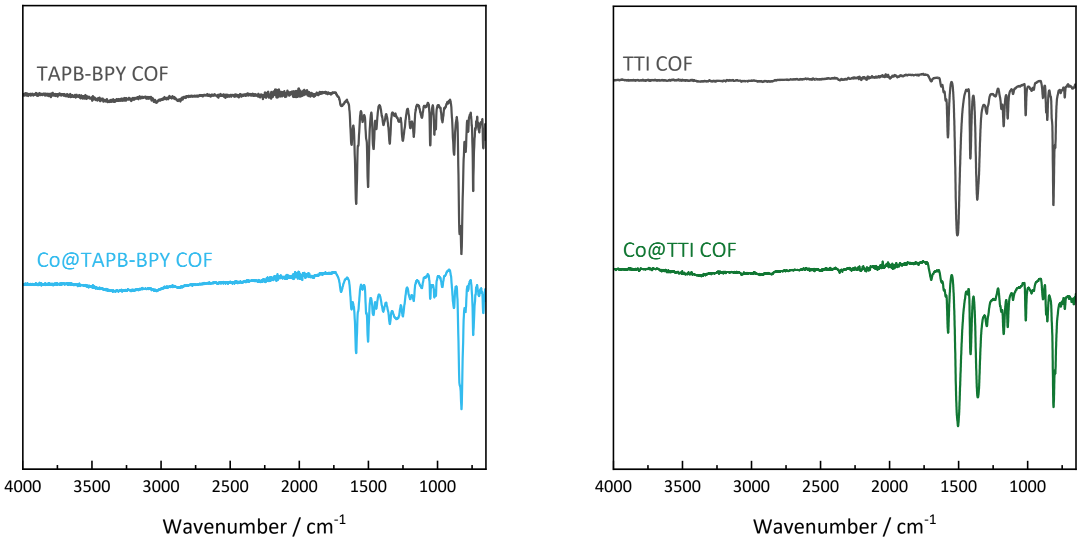

Figure S 92: FTIR spectra of pristine and cobalt-loaded TAPB-BPY COF (left) and TTI-COF (right).

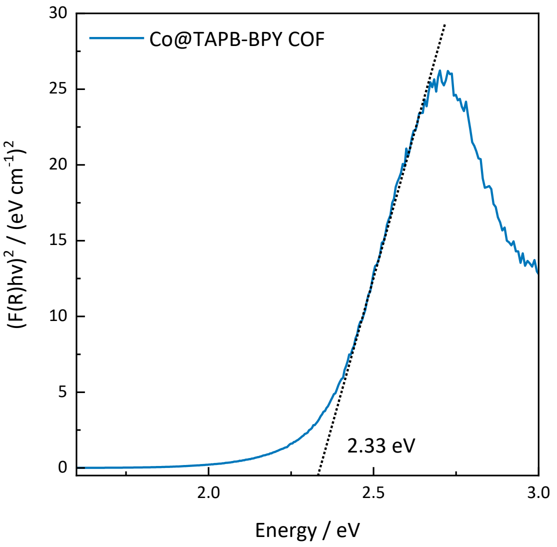

Figure S 93: Tauc plot for Co@TAPB-BPY COF with 1.038 wt% Co content. The Annotation indicates the optical band gap.

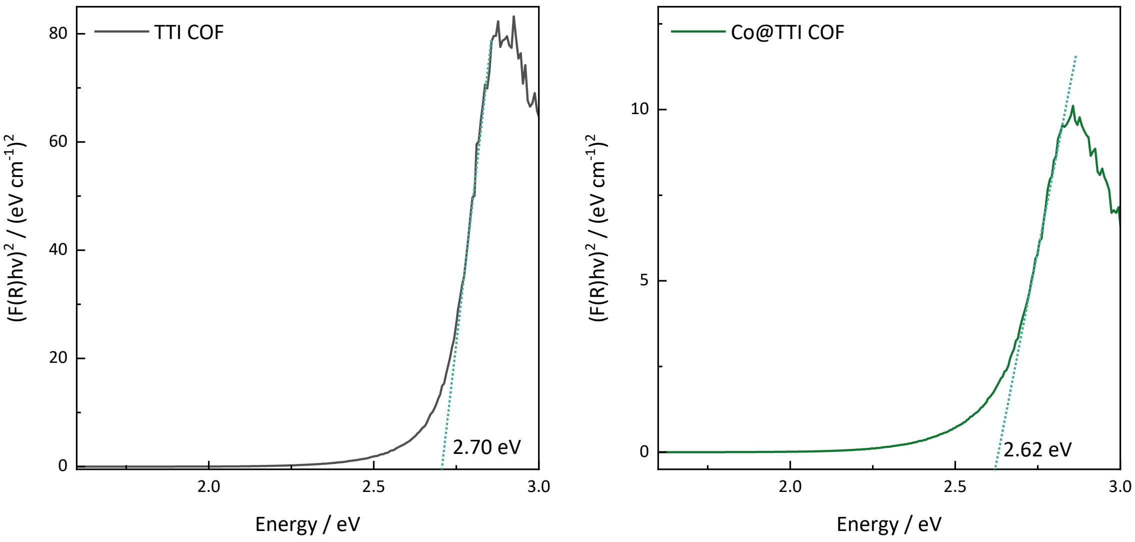

Figure S 94: Tauc plot for TTI-COF (left) and Co@TTI-COF with 0.867 wt% Co content (right). Annotations indicate the optical band gap.

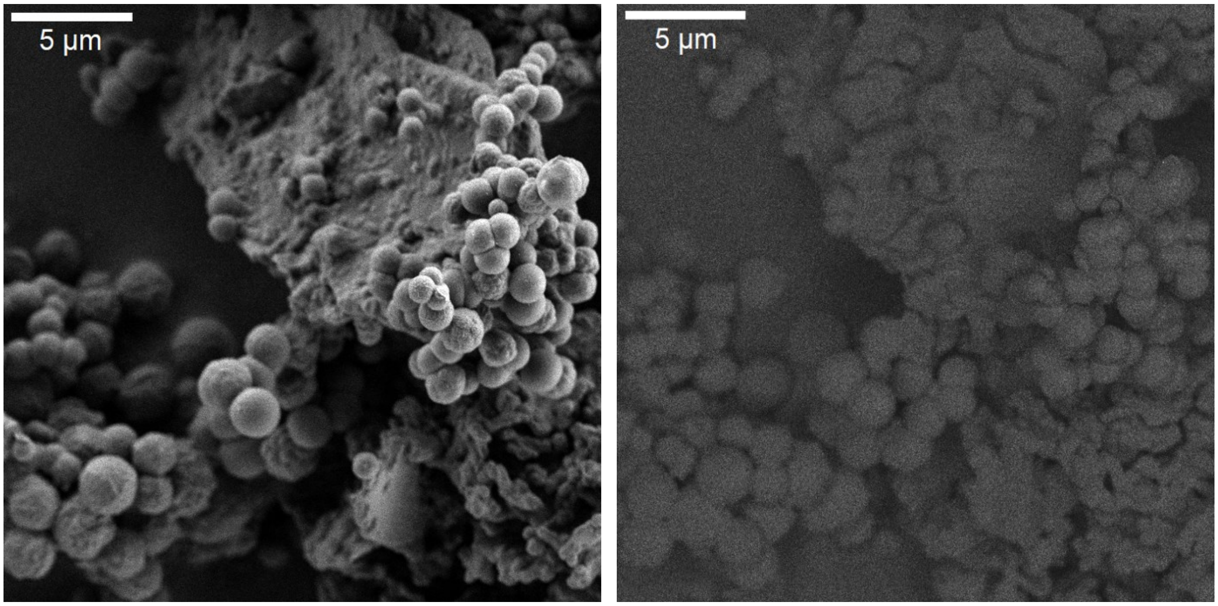

Figure S 95: SEM images of Co@TAPB-BPY COF (1 wt% Co) with secondary electron detection (left) and energy selective backscattered electron detection for material contrast illustration (right).

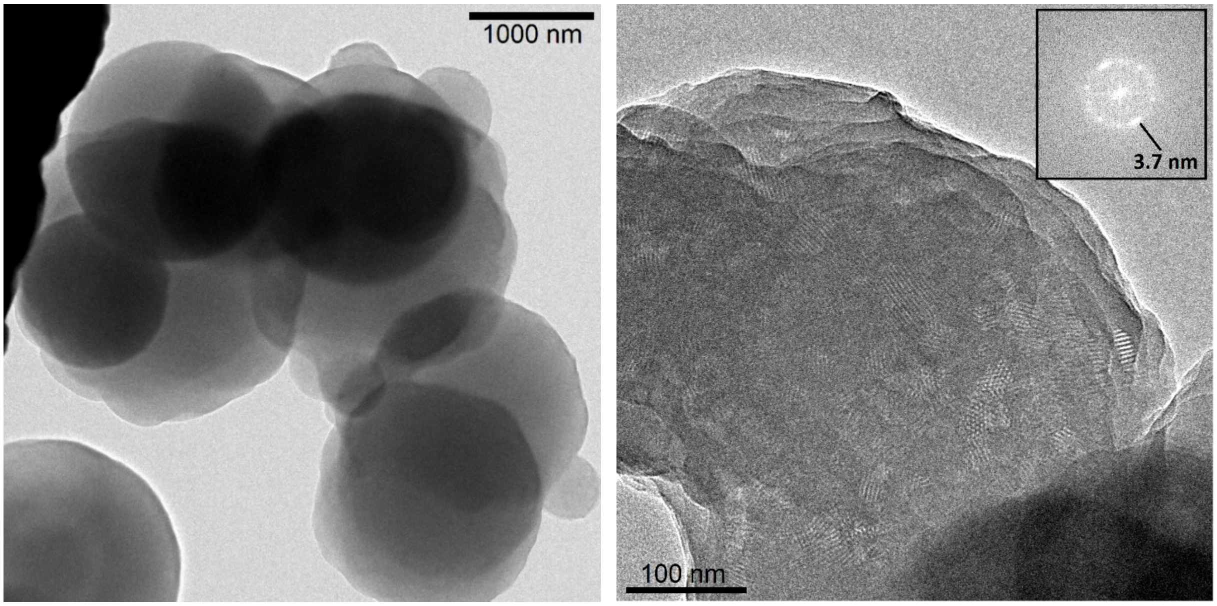

Figure S 96: TEM images of Co@TAPB-BPY COF (1 wt%). Inset shows FFT.

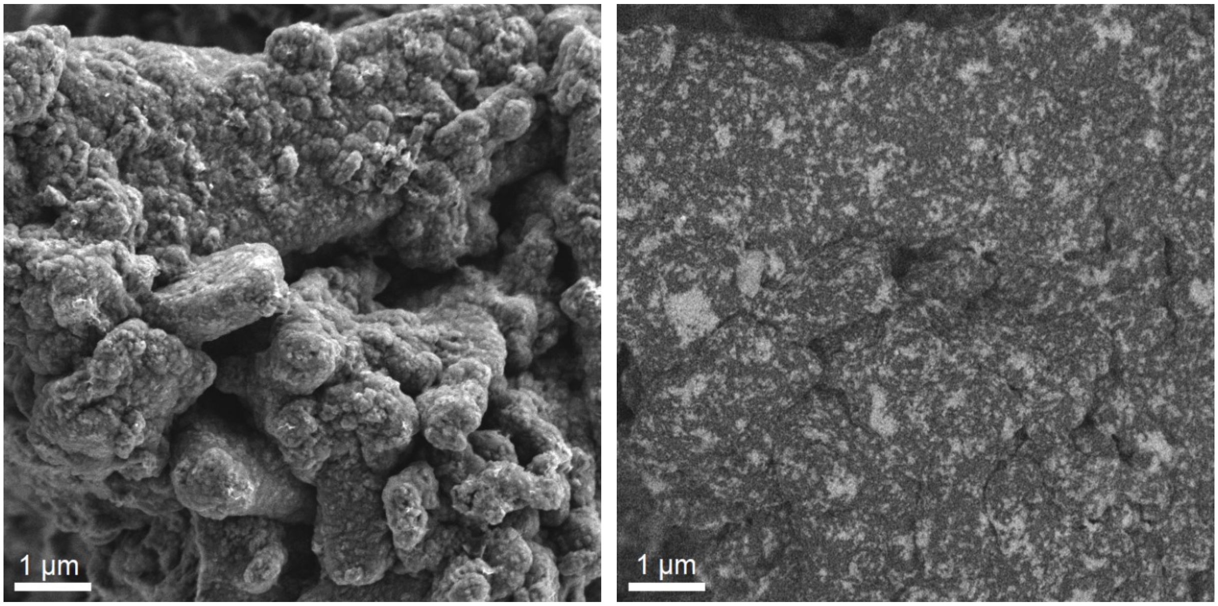

Figure S 97: SEM images of Co@TTI-COF (0.9 wt% Co) with secondary electron detection (left) and energy selective backscattered electron detection for material contrast illustration (right).

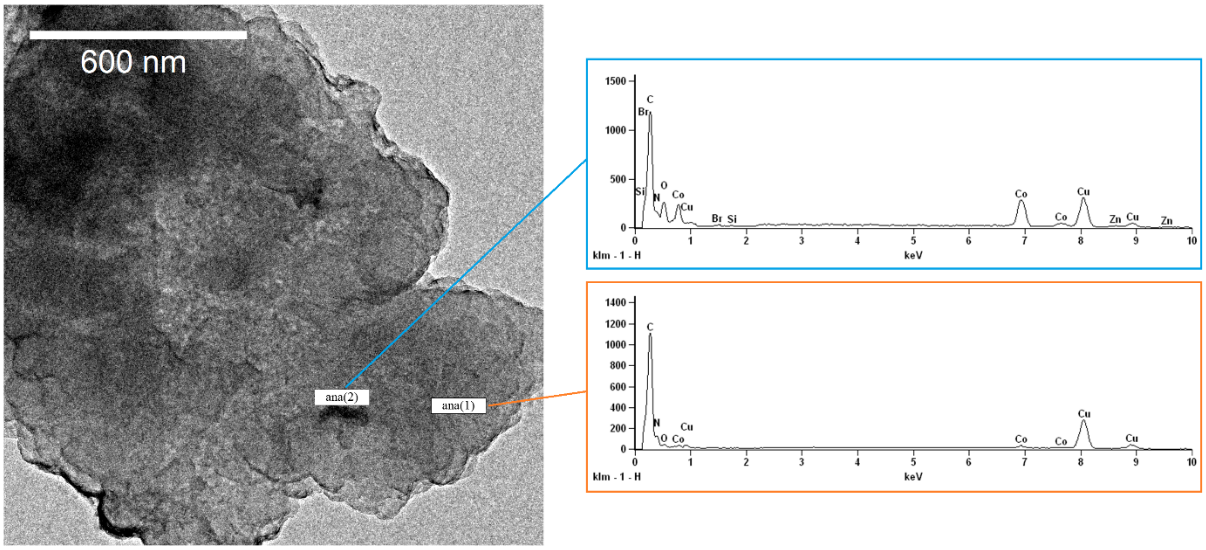

Figure S 98: TEM image of Co@TTI-COF (0.9 wt% Co) and corresponding EDX spectra collected at the designated areas illustrating inhomogeneous distribution of cobalt over the COF particles.

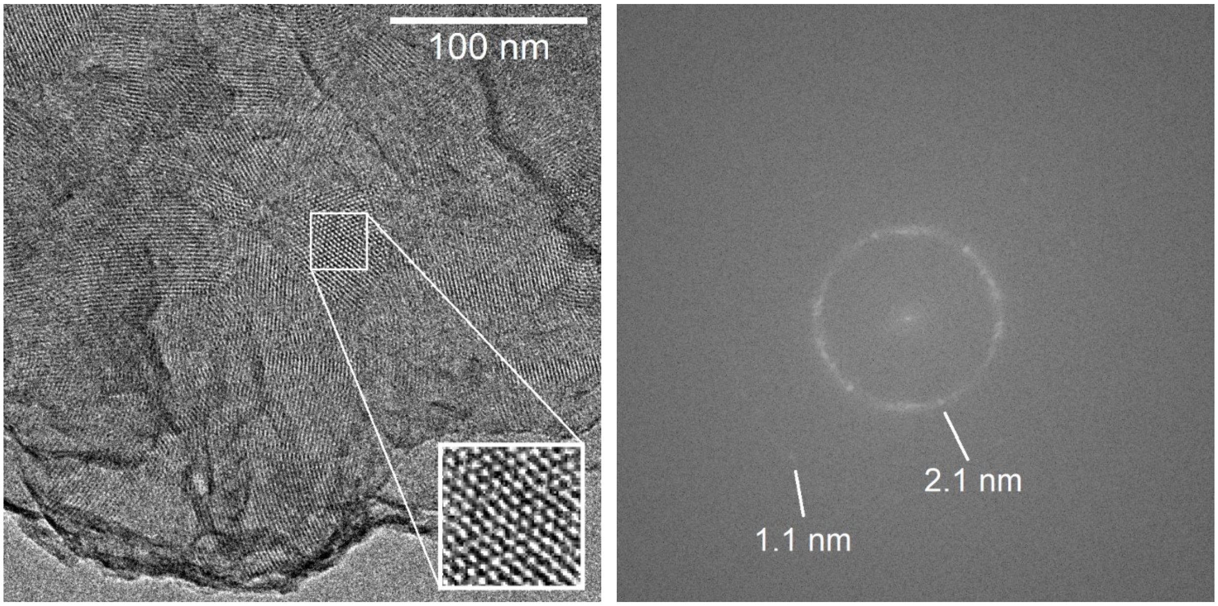

Figure S 99: TEM image of Co@TTI-COF (0.9 wt% Co) and corresponding FFT.

Table S 11: Screening results for photocatalytic oxygen evolution experiments with Co@TAPB-BPY COF and comparison to literature results.

Co / wt%	$c(AgNO_3)$	COF / medium	reactor	illumination	OER rate µmol h^{-1}	OER rate µmol h^{-1} g^{-1}	Source
0	5 mM	10 mg COF in 100 mL water	Pyrex glass reaction cell	300 W Xe lamp >420 nm	0	0 ("traces")	Ref. [28]
1.2					1.52	152	
0.25					~0.75	~75[a]	
0.5					~0.92	~92[a]	
2.5					~1.30	~130[a]	
5.1					~0.71	~71[a]	
7.5					~0.70	~70[a]	
0	10 mM	No COF in 5 mL water	flow reactor, contaminated with $RuOx@WO_3$	300 W Xe lamp >420 nm	0.1	-	This work
0	10 mM	5 mg COF in 5 mL water		300 W Xe lamp >420 nm	0.11	20	This work
1.223	10 mM	0.5 mg COF in 5 mL water		300 W Xe lamp >420 nm	0.04	8.2	This work
1.688	10 mM	5 mg COF in 5 mL water		300 W Xe lamp >420 nm	0.1	20	This work
~1	10 mM	4 mg COF in 5 mL water		300 W Xe lamp full arc	0.24	60	This work
				AM1.5	0.18	45	
				>420	0.10	25	
1.223	10 mM	0.5 mg COF in 5 mL water		300 W Xe lamp >420 nm	0.1	196	This work
1.038	5 mM	2 mg COF in 20 mL water	bulk reactor	Solar simulator >420 nm	0	0	This work

[a]: value extracted from graph.

Table S 12: Screening results for photocatalytic oxygen evolution experiments with Co@TTI-COF and comparison to literature results.

Co / wt%	c($AgNO_3$)	COF / medium	reactor	illumination	OER rate µmol h^{-1}	OER rate µmol h^{-1} g^{-1}	Source
0	10 mM	10 mg COF<br>100 mg La_2O_3<br>in 50 mL water	quartz tube?	300 W Xe lamp<br>>420 nm	~0.13	~12.5[a]	Ref. [27]
~2	10 mM	10 mg COF<br>100 mg La_2O_3<br>in 50 mL water	quartz tube?	300 W Xe lamp<br>>420 nm	~0.4	~37[a]	Ref. [27]
0.867	10 mM	1.0 mg COF<br>10 mg La_2O_3<br>in 5 mL water	flow reactor, contaminated	Solar simulator >420 nm	0.04	40	This work
				AM1.5	0.2	200	
0.324	10 mM	5.0 mg COF<br>10 mg La_2O_3<br>in 5 mL water	flow reactor, contaminated	300 W Xe lamp >420 nm	0.12	24	This work
0.867	10 mM	1.0 mg COF<br>10 mg La_2O_3<br>in 5 mL water	flow reactor, pristine	Solar simulator AM1.5	0	0	This work
				>420 nm	0	0	

[a]: value extracted from graph.

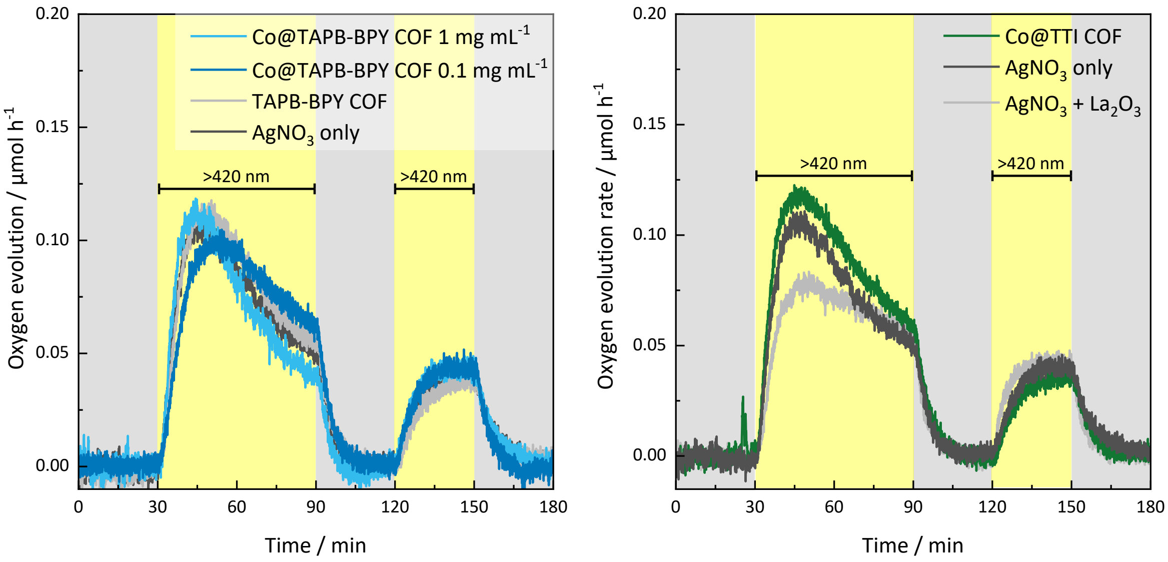

Figure S 100: Photocatalytic oxygen evolution experiments with literature-known COFs in a contaminated flow-reactor. Reaction conditions: 5.0 mg COF (or 0.5 mg if stated), $AgNO_3$ (10 mM, 5 mL), 300 W Xenon lamp, >420 nm longpass filter. 10 mg La_2O_3 added as pH buffer for Co@TTI-COF.

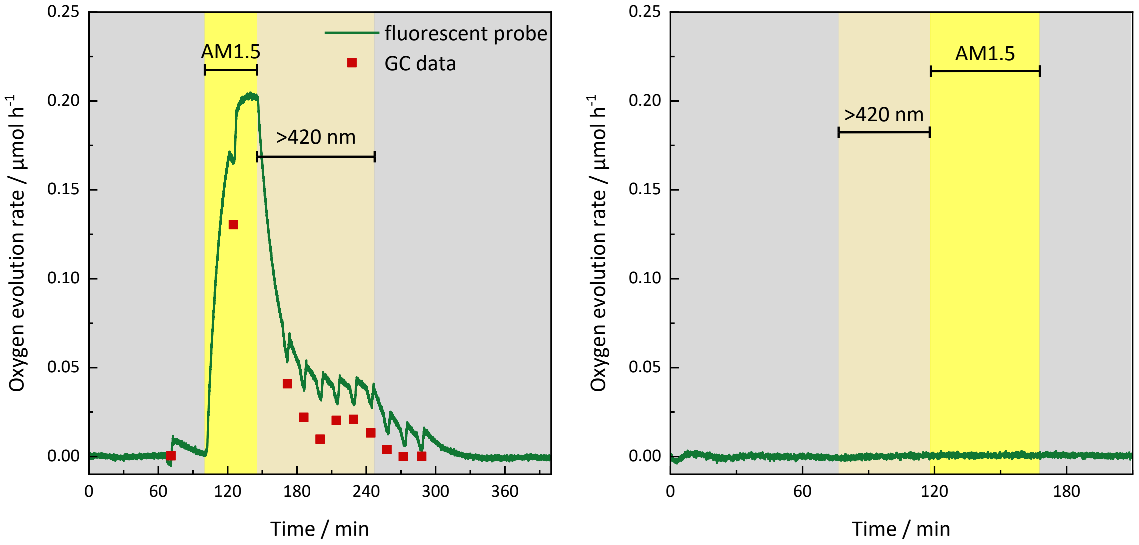

Figure S 101: Oxygen evolution experiment with Co@TTI COF under literature-inspired conditions (1 mg COF with 0.867 wt% Co, 10 mg La_2O_3, 5 mL 10 mM $AgNO_3$). Illumination with a solar simulator as annotated (1 sun). Periodic peaks are due to pressure variations upon GC sampling. Reactions conducted in a contaminated (left) and a pristine photoreactor (right).

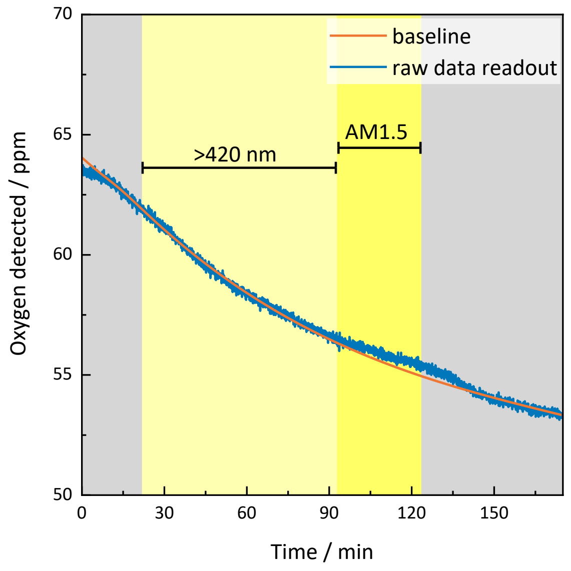

Figure S 102: Raw data and derived baseline for the photocatalytic oxygen evolution experiment with Co@TAPB-BPY COF in a bulk photoreactor without glass frit (Figure S 63). Reaction condition: 2 mg COF, 20 mL AgNO3, 5 mM. Illumination with an AAA sun simulator (1 sun) and optical filters as specified. Grey areas represent dark reaction conditions. The negative slope is due to ongoing degassing of the bulk/flow hybrid setup.

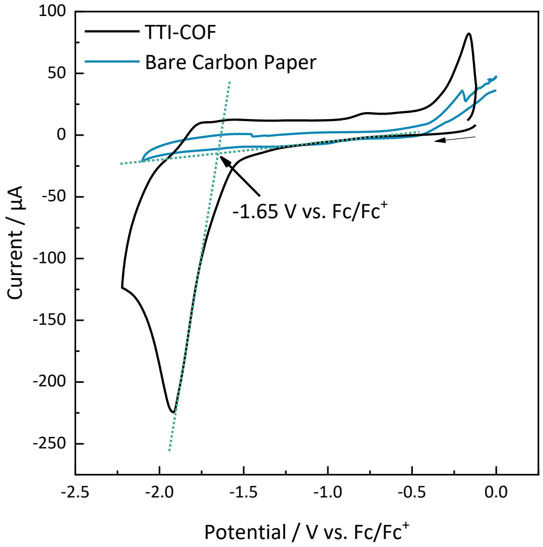

Figure S 103: Cyclic voltammogram TTI-COF. COF measured after deposition on carbon paper working electrode.

Table S 13: Comparison of band positions and related data for TTI-COF.

COF	$E_{red, onset}$	E_{CB}	Opt. band gap	E_{VB}	Source
TTI-COF	-1.65 V vs. Fc/Fc^+	-3.45 eV	2.70 eV	-6.15 eV	This work
TTI-COF („I-TST“)		-3.43 eV	2.93 eV	-6.27 eV	Ref. [27][a]

Conversion of potentials done according to E(V *vs.* vac) = -(E(V *vs.* Fc/Fc+) + 5.1) eV [a]: Band positions not experimentally determined, but calculated.

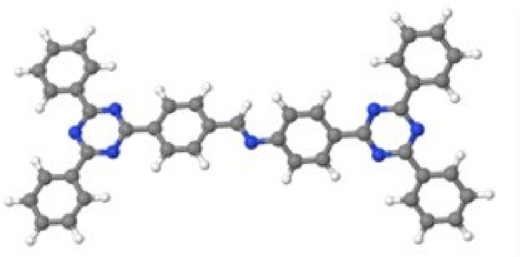

Figure S 104: Cluster model used to calculate IP and EA of TTI-COF.

Table S 14: Ionisation potential (IP) and electron affinity (EA) values predicted for cluster model of TTI-COF in water and acetonitrile. All values are given in V vs. SHE.

	In acetonitrile		In water	
	IP	EA	IP	EA
TTI-COF	1.37	-1.53	1.34	-1.50

Representative COF cluster used for the prediction is depicted in Figure S 104.

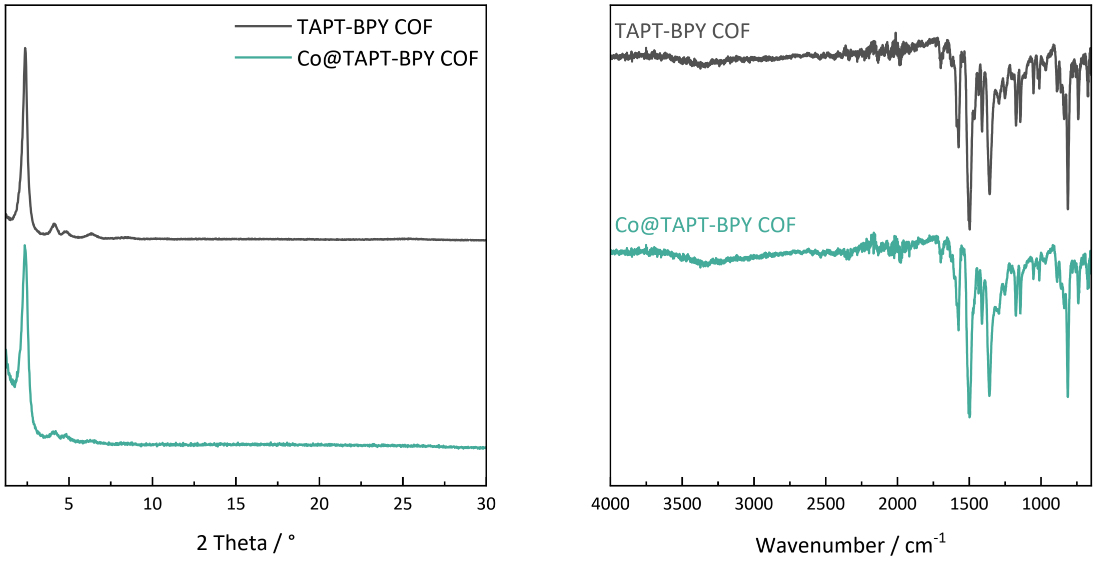

Figure S 105: XRPD patterns (left) and FTIR spectra (right) of pristine and cobalt-loaded TAPT-BPY COF.

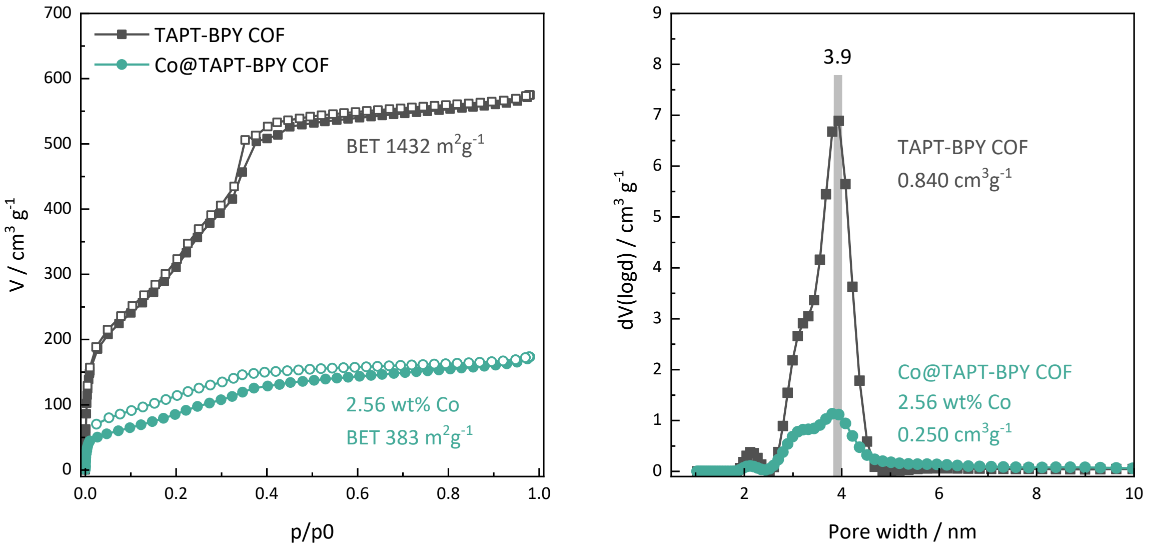

Figure S 106: Nitrogen sorption isotherms at 77 K (left) and pore size distribution (right) for TAPT-BPY COF before and after postsynthetic loading with $Co(NO_3)_2$. Filled and open symbols represent the adsorption and the desorption branches, respectively. The pore size distribution was obtained from a QSDFT kernel for cylindrical pores (adsorption branch). The cobalt content was determined *via* ICP-OES.

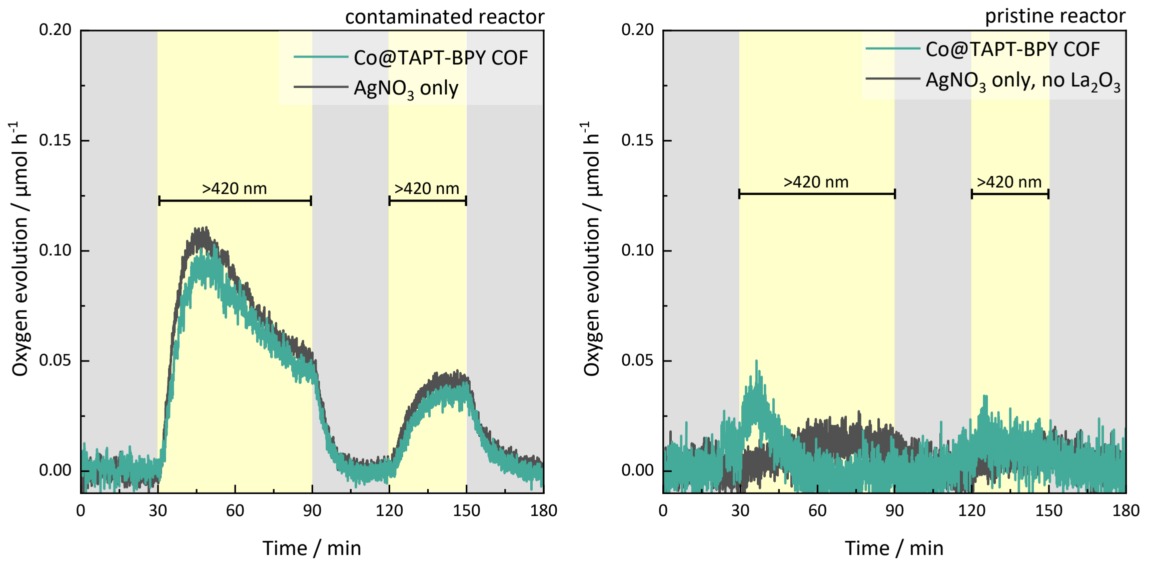

Figure S 107: Left: Photocatalytic oxygen evolution experiment with Co@TAPT-BPY COF (2.56 wt% Co) in a contaminated flow reactor. Reaction conditions: 5.0 mg COF, $AgNO_3$ (10 mM, 5 mL), no La_2O_3, 300 W Xenon lamp, >420 nm longpass filter. Right: Photocatalytic oxygen evolution experiment with Co@TAPT-BPY COF (2.56 wt% Co) in a pristine flow reactor. Reaction conditions: 5.0 mg COF, $AgNO_3$ (10 mM, 5 mL), 300 W Xenon lamp, >420 nm longpass filter, 10 mg La_2O_3 (only for COF measurement).

Table S 15: Summary table on oxygen evolution / catalytic activity of materials tested within this work.

Material	Setup	Conditions	Oxygen evolution	Reference
TAPB-BPY COF	flow reactor with glass frit	20 mM $Na_2S_2O_8$, 300 W Xe lamp, not filtered	no	Figure S 52
Ir@TAPB-BPY COF	flow reactor with glass frit	20 mM $Na_2S_2O_8$, 300 W Xe lamp, not filtered	yes	Figure S 52
Ir@TAPB-BPY COF	flow reactor with glass frit	20 mM $Na_2S_2O_8$, 300 W Xe lamp, AM1.5 filter	no	Figure S 52
Ir@TAPB-BPY COF	flow reactor with glass frit	without $Na_2S_2O_8$<br>300 W Xe lamp, not filtered or AM1.5-filtered	no	Figure S 52
$Na_2S_2O_8$ only	flow reactor with glass frit	20 mM $Na_2S_2O_8$<br>300 W Xe lamp, not filtered or AM1.5-filtered	yes	Figure S 52
TAPB-BPY COF	flow reactor with glass frit, contaminated	10 mM $AgNO_3$	yes	Figure S 54
Ir@TAPB-BPY COF	flow reactor with glass frit, contaminated	10 mM $AgNO_3$	yes	Figure S 54
$AgNO_3$only	flow reactor with glass frit, contaminated	10 mM $AgNO_3$<br>300 W Xe lamp, not filtered, AM1.5 or 420 nm longpass-filtered	yes	Figure S 54
Ir@TAPB-BPY COF	flow reactor with glass frit, contaminated	10 mM $AgNO_3$<br>300 W Xe lamp, not filtered, AM1.5 or 420 nm longpass-filtered	yes	Figure S 54

Co@TAPB-BPY COF	flow reactor with glass frit, contaminated	various, see Table S 11	yes	Table S 11
Co@TAPB-BPY COF	bulk photoreactor without glass frit, inert gas flow through PTFE tube<br><br>(Figure S 63)	2 mg COF, 20 mL $AgNO_3$, 5 mM<br><br>AAA sun simulator, AM1.5 or 420 nm longpass-filtered (1 sun)	no	Figure S 102
$AgNO_3$ + pristine glass frit	bulk photoreactor with suspended glass frit, inert gas flow through PTFE tube<br><br>(Figure S 63)	10 mM $AgNO_3$ (10 mL)<br><br>AAA sun simulator, AM1.5 filter (1 sun)	no	Figure S 63
$AgNO_3$ only	bulk photoreactor without glass frit, inert gas flow through PTFE tube<br><br>(Figure S 63)	10 mM $AgNO_3$ (10 mL)<br><br>AAA sun simulator, AM1.5 filter (1 sun)	no	Figure S 63
$AgNO_3$ only	flow reactor with glass frit, contaminated	10 mM $AgNO_3$	yes	Figure S 64
$AgNO_3$ only	flow reactor with glass frit, pristine	10 mM $AgNO_3$	no	Figure S 64
$AgNO_3$ only	flow reactor with glass frit, contaminated	10 mM $AgNO_3$<br><br>AAA sun simulator, AM1.5 or 420 nm longpass-filtered (1 sun)	yes	Figure S 65<br><br>Figure S 55

$AgNO_3$ only	flow reactor with glass frit, pristine	10 mM $AgNO_3$<br>AAA sun simulator, AM1.5 or 420 nm longpass-filtered (1 sun)	no	Figure S 65
Ir@TAPB-BPY COF	flow reactor with glass frit, pristine	1.9 wt% Ir<br>AAA sun simulator, AM1.5 or 420 nm longpass-filtered (1 sun)	no	Figure S 71
Ir@TAPT-BPY COF	flow reactor with glass frit, pristine	2.3 wt% Ir<br>10 mM $AgNO_3$	no	Figure S 81
Co@TTI COF	flow reactor with glass frit, contaminated	5 mg COF, 0.324 wt% Co, 10 mg La_2O_3, 10 mM AgNO3	yes	Figure S 100<br>Table S 12
Co@TTI COF	flow reactor with glass frit, contaminated	1 mg COF, 0.867 wt% Co, 10 mg La_2O_3, 5 mL 10 mM $AgNO_3$<br>AAA sun simulator, AM1.5 (1 sun)	yes	Figure S 101<br>Table S 12
Co@TTI COF	flow reactor with glass frit, pristine	1 mg COF, 0.867 wt% Co, 10 mg La_2O_3, 5 mL 10 mM $AgNO_3$<br>AAA sun simulator, AM1.5	no	Figure S 101<br>Table S 12
Co@TAPT-BPY COF	flow reactor with glass frit, contaminated	2.56 wt% Co<br>10 mM $AgNO_3$	yes	Figure S 107
Co@TAPT-BPY COF	flow reactor with glass frit, pristine	2.56 wt% Co<br>10 mM $AgNO_3$<br>10 mg La_2O_3	No	Figure S 107

If not stated otherwise: flow reactor with glass frit (Figure S 43), 5 mg COF, 5 mL aqueous suspension, 420 nm longpass filter, 300 W Xe lamp

References

(1) Swift, P. Adventitious Carbon-The Panacea for Energy Referencing? *Surf. Interface Anal.* **1982**, *4*, 47–51.

(2) Barr, T. L.; Seal, S. Nature of the use of adventitious carbon as a binding energy standard. *J. Vac. Sci. Technol. A* **1995**, *13*, 1239–1246.

(3) Biesinger, M. C. Accessing the robustness of adventitious carbon for charge referencing (correction) purposes in XPS analysis: Insights from a multi-user facility data review. *Appl. Surf. Sci.* **2022**, *597*, 153681.

(4) Biswal, B. P.; Vignolo-González, H. A.; Banerjee, T.; Grunenberg, L.; Savasci, G.; Gottschling, K.; Nuss, J.; Ochsenfeld, C.; Lotsch, B. V. Sustained Solar H2 Evolution from a Thiazolo5,4-dthiazole-Bridged Covalent Organic Framework and Nickel-Thiolate Cluster in Water. *J. Am. Chem. Soc.* **2019**, *141*, 11082–11092.

(5) Trenker, S.; Grunenberg, L.; Banerjee, T.; Savasci, G.; Poller, L. M.; Muggli, K. I. M.; Haase, F.; Ochsenfeld, C.; Lotsch, B. V. A flavin-inspired covalent organic framework for photocatalytic alcohol oxidation. *Chem. Sci.* **2021**, *12*, 15143–15150.

(6) Yao, L.; Rodríguez-Camargo, A.; Xia, M.; Mücke, D.; Guntermann, R.; Liu, Y.; Grunenberg, L.; Jiménez-Solano, A.; Emmerling, S. T.; Duppel, V.; Sivula, K.; Bein, T.; Qi, H.; Kaiser, U.; Grätzel, M.; Lotsch, B. V. Covalent Organic Framework Nanoplates Enable Solution-Processed Crystalline Nanofilms for Photoelectrochemical Hydrogen Evolution. *J. Am. Chem. Soc.* **2022**, *144*, 10291–10300.

(7) Sick, T.; Hufnagel, A. G.; Kampmann, J.; Kondofersky, I.; Calik, M.; Rotter, J. M.; Evans, A.; Döblinger, M.; Herbert, S.; Peters, K.; Böhm, D.; Knochel, P.; Medina, D. D.; Fattakhova-

Rohlfing, D.; Bein, T. Oriented Films of Conjugated 2D Covalent Organic Frameworks as Photocathodes for Water Splitting. *J. Am. Chem. Soc.* **2018**, *140*, 2085–2092.

(8) Guiglion, P.; Butchosa, C.; Zwijnenburg, M. A. Polymeric watersplitting photocatalysts; a computational perspective on the water oxidation conundrum. *J. Mater. Chem. A* **2014**, *2*, 11996–12004.

(9) Guiglion, P.; Monti, A.; Zwijnenburg, M. A. Validating a Density Functional Theory Approach for Predicting the Redox Potentials Associated with Charge Carriers and Excitons in Polymeric Photocatalysts. *J. Phys. Chem. C* **2017**, *121*, 1498–1506.

(10) Woods, D. J.; Hillman, S. A. J.; Pearce, D.; Wilbraham, L.; Flagg, L. Q.; Duffy, W.; McCulloch, I.; Durrant, J. R.; Guilbert, A. A. Y.; Zwijnenburg, M. A.; Sprick, R. S.; Nelson, J.; Cooper, A. I. Side-chain tuning in conjugated polymer photocatalysts for improved hydrogen production from water. *Energy Environ. Sci.* **2020**, *13*, 1843–1855.

(11) Nørskov, J. K.; Rossmeisl, J.; Logadottir, A.; Lindqvist, L.; Kitchin, J. R.; Bligaard, T.; Jónsson, H. Origin of the Overpotential for Oxygen Reduction at a Fuel-Cell Cathode. *J. Phys. Chem. B* **2004**, *108*, 17886–17892.

(12) Rossmeisl, J.; Nørskov, J. K.; Taylor, C. D.; Janik, M. J.; Neurock, M. Calculated phase diagrams for the electrochemical oxidation and reduction of water over Pt(111). *J. Phys. Chem. B* **2006**, *110*, 21833–21839.

(13) Rossmeisl, J.; Qu, Z.-W.; Zhu, H.; Kroes, G. J.; Nørskov, J. K. Electrolysis of water on oxide surfaces. *J. Electroanal. Chem.,* **2007**, *607*, 83–89.

(14) Becke, A. D. Density-functional thermochemistry. III. The role of exact exchange. *J. Chem. Phys.* **1993**, *98*, 5648–5652.

(15) Lee, C.; Yang, W.; Parr, R. G. Development of the Colle-Salvetti correlation-energy formula into a functional of the electron density. *Phys. Rev. B* **1988**, *37*, 785–789.

(16) Vosko, S. H.; Wilk, L.; Nusair, M. Accurate spin-dependent electron liquid correlation energies for local spin density calculations: a critical analysis. *Can. J. Phys.* **1980**, *58*, 1200–1211.

(17) Stephens, P. J.; Devlin, F. J.; Chabalowski, C. F.; Frisch, M. J. Ab Initio Calculation of Vibrational Absorption and Circular Dichroism Spectra Using Density Functional Force Fields. *J. Phys. Chem.* **1994**, *98*, 11623–11627.

(18) Schäfer, A.; Horn, H.; Ahlrichs, R. Fully optimized contracted Gaussian basis sets for atoms Li to Kr. *J. Chem. Phys.* **1992**, *97*, 2571–2577.

(19) Weigend, F.; Ahlrichs, R. Balanced basis sets of split valence, triple zeta valence and quadruple zeta valence quality for H to Rn: Design and assessment of accuracy. *Phys. Chem. Chem. Phys.* **2005**, *7*, 3297–3305.

(20) Andrae, D.; Häußermann, U.; Dolg, M.; Stoll, H.; Preuß, H. Energy-adjusted ab initio pseudopotentials for the second and third row transition elements. *Theor. Chim. Acta* **1990**, *77*, 123–141.

(21) Balasubramani, S. G.; Chen, G. P.; Coriani, S.; Diedenhofen, M.; Frank, M. S.; Franzke, Y. J.; Furche, F.; Grotjahn, R.; Harding, M. E.; Hättig, C.; Hellweg, A.; Helmich-Paris, B.; Holzer, C.; Huniar, U.; Kaupp, M.; Marefat Khah, A.; Karbalaei Khani, S.; Müller, T.; Mack, F.; Nguyen, B. D.; Parker, S. M.; Perlt, E.; Rappoport, D.; Reiter, K.; Roy, S.; Rückert, M.; Schmitz, G.; Sierka,

M.; Tapavicza, E.; Tew, D. P.; van Wüllen, C.; Voora, V. K.; Weigend, F.; Wodyński, A.; Yu, J. M. TURBOMOLE: Modular program suite for ab initio quantum-chemical and condensed-matter simulations. *J. Chem. Phys.* **2020**, *152*, 184107.

(22) Furche, F.; Ahlrichs, R.; Hättig, C.; Klopper, W.; Sierka, M.; Weigend, F. Turbomole. *Wiley Interdiscip. Rev. Comput. Mol. Sci.* **2014**, *4*, 91–100.

(23) Klamt, A.; Schüürmann, G. COSMO: A NEW Approach to Dielectric Screening in Solvents with Explicit Expressions for the Screening Energy and its Gradient. *J. Chem. Soc., Perkin Trans. 2* **1993**, 799–805.

(24) Jakoobi, M.; Halcovitch, N.; Whitehead, G. F. S.; Sergeev, A. G. Selective Arene Cleavage by Direct Insertion of Iridium into the Aromatic Ring. *Angew. Chem. Int. Ed.* **2017**, *56*, 3266–3269.

(25) Yang, Z.; Zhu, Z.; Luo, R.; Qiu, X.; Liu, J.; Yang, J.-K.; Tang, W. Iridium-catalyzed highly efficient chemoselective reduction of aldehydes in water using formic acid as the hydrogen source. *Green Chem.* **2017**, *19*, 3296–3301.

(26) Haase, F.; Troschke, E.; Savasci, G.; Banerjee, T.; Duppel, V.; Dörfler, S.; Grundei, M. M. J.; Burow, A. M.; Ochsenfeld, C.; Kaskel, S.; Lotsch, B. V. Topochemical conversion of an imine- into a thiazole-linked covalent organic framework enabling real structure analysis. *Nat. Commun.* **2018**, *9*, 2600.

(27) Wan, Y.; Wang, L.; Xu, H.; Wu, X.; Yang, J. A Simple Molecular Design Strategy for Two-Dimensional Covalent Organic Framework Capable of Visible-Light-Driven Water Splitting. *J. Am. Chem. Soc.* **2020**, *142*, 4508–4516.

(28) Chen, J.; Tao, X.; Li, C.; Ma, Y.; Tao, L.; Zheng, D.; Zhu, J.; Li, H.; Li, R.; Yang, Q. Synthesis of bipyridine-based covalent organic frameworks for visible-light-driven photocatalytic water oxidation. *Appl. Catal. B* **2020**, *262*, 118271.

(29) Chen, H.; Gardner, A. M.; Lin, G.; Zhao, W.; Wang, X.; Bahri, M.; Browning, N. D.; Xu, X.; Li, X. Triazine-Based Covalent Organic Framework for Photocatalytic Water Oxidation: The Role of Bipyridine Ligand and Cobalt Coordination. *J. Phys. Chem. C* **2023**, *127*, 14137–14145.

(30) Vignolo-González, H. A.; Gouder, A.; Laha, S.; Duppel, V.; Carretero-Palacios, S.; Jiménez-Solano, A.; Oshima, T.; Schützendübe, P.; Lotsch, B. V. Morphology Matters: 0D/2D WO3 Nanoparticle-Ruthenium Oxide Nanosheet Composites for Enhanced Photocatalytic Oxygen Evolution Reaction Rates. *Adv. Energy Mater.* **2023**, *13*, 2203315.

(31) Firouzabadi, H.; Salehi, P.; Sardarian, A. R.; Seddighi, M. Oxidation of Benzylic Hydrocarbons to Carbonyl Compounds by Tetrapyridinesilver(II) Peroxydisulfate Ag(Py)4S2O8 Under Non-Aqueous and Aprotic Condition. *Synth. Commun.* **1991**, *21*, 1121–1127.

(32) Sypaseuth, F. D.; Matlachowski, C.; Weber, M.; Schwalbe, M.; Tzschucke, C. C. Electrocatalytic carbon dioxide reduction by using cationic pentamethylcyclopentadienyl-iridium complexes with unsymmetrically substituted bipyridine ligands. *Chem. Eur. J.* **2015**, *21*, 6564–6571.

(33) Golczak, S.; Kanciurzewska, A.; Fahlman, M.; Langer, K.; Langer, J. Comparative XPS surface study of polyaniline thin films. *Solid State Ion.* **2008**, *179*, 2234–2239.

(34) Kapteijn, F.; Moulijn, J. A.; Matzner, S.; Boehm, H.-P. The development of nitrogen functionality in model chars during gasification in CO and O2. *Carbon* **1999**, *37*, 1143–1150.

(35) Wang, H.; Maiyalagan, T.; Wang, X. Review on Recent Progress in Nitrogen-Doped Graphene: Synthesis, Characterization, and Its Potential Applications. *ACS Catal.* **2012**, *2*, 781–794.

(36) Rabchinskii, M. K.; Saveliev, S. D.; Stolyarova, D. Y.; Brzhezinskaya, M.; Kirilenko, D. A.; Baidakova, M. V.; Ryzhkov, S. A.; Shnitov, V. V.; Sysoev, V. V.; Brunkov, P. N. Modulating nitrogen species via N-doping and post annealing of graphene derivatives: XPS and XAS examination. *Carbon* **2021**, *182*, 593–604.

(37) Kaushik, V. K. XPS Core Level Spectra and Auger Parameters for some Silver Compounds. *J. Electron Spectrosc. Relat. Phenom.* **1991**, *56*, 273–277.

(38) Bêche, E.; Charvin, P.; Perarnau, D.; Abanades, S.; Flamant, G. Ce 3d XPS investigation of cerium oxides and mixed cerium oxide (CexTiyOz). *Surf. Interface Anal.* **2008**, *40*, 264–267.

(39) Paparazzo, E.; Ingo, G. M.; Zacchetti, N. X-ray induced reduction effects at CeO2 surfaces: An x-ray photoelectron spectroscopy study. *J. Vac. Sci. Technol. A* **1991**, *9*, 1416–1420.

(40) Mullins, D. R. The surface chemistry of cerium oxide. *Surf. Sci. Rep.* **2015**, *70*, 42–85.

(41) Savini, A.; Belanzoni, P.; Bellachioma, G.; Zuccaccia, C.; Zuccaccia, D.; Macchioni, A. Activity and degradation pathways of pentamethyl-cyclopentadienyl-iridium catalysts for water oxidation. *Green Chem.* **2011**, *13*, 3360–3374.

(42) Parent, A. R.; Crabtree, R. H.; Brudvig, G. W. Comparison of primary oxidants for water-oxidation catalysis. *Chem. Soc. Rev.* **2013**, *42*, 2247–2252.

(43) Mills, A.; Valenzuela, M. A. The photo-oxidation of water by sodium persulfate, and other electron acceptors, sensitised by TiO2. *J. Photochem. Photobiol. A* **2004**, *165*, 25–34.

(44) Vignolo-González, H. A.; Laha, S.; Jiménez-Solano, A.; Oshima, T.; Duppel, V.; Schützendübe, P.; Lotsch, B. V. Toward Standardized Photocatalytic Oxygen Evolution Rates Using RuO2@TiO2 as a Benchmark. *Matter* **2020**, *3*, 464–486.

(45) Schneider, J.; Bahnemann, D. W. Undesired Role of Sacrificial Reagents in Photocatalysis. *J. Phys. Chem. Lett.* **2013**, *4*, 3479–3483.

(46) Ike, I. A.; Linden, K. G.; Orbell, J. D.; Duke, M. Critical review of the science and sustainability of persulphate advanced oxidation processes. *Chem. Eng. J.* **2018**, *338*, 651–669.

(47) Wacławek, S.; Lutze, H. V.; Grübel, K.; Padil, V. V.; Černík, M.; Dionysiou, D. Chemistry of persulfates in water and wastewater treatment: A review. *Chem. Eng. J.* **2017**, *330*, 44–62.

(48) Liu, H.; Bruton, T. A.; Li, W.; van Buren, J.; Prasse, C.; Doyle, F. M.; Sedlak, D. L. Oxidation of Benzene by Persulfate in the Presence of Fe(III)- and Mn(IV)-Containing Oxides: Stoichiometric Efficiency and Transformation Products. *Environ. Sci. Technol.* **2016**, *50*, 890–898.

(49) Bartlett, P. D.; Cotman, J. D. The Kinetics of the Decomposition of Potassium Persulfate in Aqueous Solutions of Methanol. *J. Am. Chem. Soc.* **1949**, *71*, 1419–1422.

(50) Ivanova, I.; Kandiel, T. A.; Cho, Y.-J.; Choi, W.; Bahnemann, D. Mechanisms of Photocatalytic Molecular Hydrogen and Molecular Oxygen Evolution over La-Doped NaTaO3 Particles: Effect of Different Cocatalysts and Their Specific Activity. *ACS Catal.* **2018**, *8*, 2313–2325.

(51) Jeon, T. H.; Monllor-Satoca, D.; Moon, G.-H.; Kim, W.; Kim, H.-I.; Bahnemann, D. W.; Park, H.; Choi, W. Ag(I) ions working as a hole-transfer mediator in photoelectrocatalytic water oxidation on WO3 film. *Nat. Commun.* **2020**, *11*, 967.

(52) Po, H. N.; Swinehart, J. H.; Allen, T. L. Kinetics and mechanism of the oxidation of water by silver(II) in concentrated nitric acid solution. *Inorg. Chem.* **1968**, *7*, 244–249.

(53) McMillan, J. A.; Smaller, B. Paramagnetic Resonance of Some Silver (II) Compounds. *J. Chem. Phys.* **1961**, *35*, 1698–1701.

(54) Buch, T. Paramagnetic Resonance Spectra of a Silver (II) Complex. *J. Chem. Phys.* **1965**, *43*, 761–762.

(55) Mogi, H.; Okazaki, M.; Nishioka, S.; Maeda, K. In situ formation of a molecular cobalt(III)/AgCl photocatalyst for visible-light water oxidation. *Sustain. Energy Fuels* **2021**, *5*, 5694–5698.

(56) Wang, X.; Li, S.; Yu, H.; Yu, J.; Liu, S. Ag2O as a new visible-light photocatalyst: self-stability and high photocatalytic activity. *Chem. Eur. J.* **2011**, *17*, 7777–7780.

(57) Fathi, F.; Schlitt, M.; Pedersen, D. B.; Kraatz, H.-B. Chemical behavior of electrochemically generated nanostructured silver surfaces. *Langmuir* **2011**, *27*, 12098–12105.

(58) Abid, J. P.; Wark, A. W.; Brevet, P. F.; Girault, H. H. Preparation of silver nanoparticles in solution from a silver salt by laser irradiation. *Chem. Commun.* **2002**, 792–793.

(59) Mahlmann, H. A.; Willmarth, T. E. Radiolytic and Photolytic Reduction of Aqueous Silver Nitrate Solutions. *Nature* **1964**, *202*, 590–591.

(60) Konishi, Y.; Saijo, H.; Hada, H.; Tamura, M. Formation of silver mirror by photolysis of thin films of high molecular weight silver compounds. *Nature* **1977**, *268*.

(61) Shen, Z.; Duan, H.; Frey, H. Water-Soluble Fluorescent Ag Nanoclusters Obtained from Multiarm Star Poly(acrylic acid) as "Molecular Hydrogel" Templates. *Adv. Mater.* **2007**, *19*, 349–352.

(62) Belloni, J.; Mostafavi, M.; Remita, H.; Marignier, J.-L.; Delcourt, M.-O. Radiation-induced synthesis of mono- and multi-metallic clusters and nanocolloids. *New J. Chem.* **1998**, 1239–1255.

(63) Thomsen, C. L.; Madsen, D.; Keiding, S. R.; Tho/gersen, J.; Christiansen, O. Two-photon dissociation and ionization of liquid water studied by femtosecond transient absorption spectroscopy. *J. Chem. Phys.* **1999**, *110*, 3453–3462.

(64) Svoboda, V.; Michiels, R.; LaForge, A. C.; Med, J.; Stienkemeier, F.; Slavíček, P.; Wörner, H. J. Real-time observation of water radiolysis and hydrated electron formation induced by extreme-ultraviolet pulses. *Sci. Adv.* **2020**, *6*, eaaz0385.

(65) Hada, H.; Yonezawa, Y.; Akio, Y.; Kurakake, A. Photoreduction of silver ion in aqueous and alcoholic solutions. *J. Phys. Chem.* **1976**, *80*, 2728–2731.

(66) Temgire, M. K.; Joshi, S. S. Optical and structural studies of silver nanoparticles. *Radiat. Phys. Chem.* **2004**, *71*, 1039–1044.

(67) Ge, S.; Zhao, J.; Ma, G. Monochromatic Photolysis to Generate Silver Quantum Clusters in Polymer Matrices with Efficiently Antibio Property. *Langmuir* **2020**, *36*, 4088–4097.

(68) Cataldo, F.; Ursini, O.; Angelini, G. Synthesis of silver nanoparticles by radiolysis, photolysis and chemical reduction of AgNO3 in Hibiscus sabdariffa infusion (karkadé). *J. Radioanal. Nucl. Chem.* **2016**, *307*, 447–455.

(69) Owen, E. A.; Williams, G. I. A low-temperature X-ray camera. *J. Sci. Instrum.* **1954**, *31*, 49–54.

(70) Ladwig, M.; Kaim, W. Electronic structure of catalytic intermediates for production of H2: (C5Me5)Ir(bpy) and its conjugated acid. *J. Organomet. Chem.* **1992**, *439*, 79–90.

(71) Savini, A.; Bucci, A.; Bellachioma, G.; Rocchigiani, L.; Zuccaccia, C.; Llobet, A.; Macchioni, A. Mechanistic Aspects of Water Oxidation Catalyzed by Organometallic Iridium Complexes. *Eur. J. Inorg. Chem.* **2014**, *2014*, 690–697.

(72) Zhang, T.; deKrafft, K. E.; Wang, J.-L.; Wang, C.; Lin, W. The Effects of Electron-Donating Substituents on [Ir(bpy)Cp*Cl]+: Water Oxidation versus Ligand Oxidative Modifications. *Eur. J. Inorg. Chem.* **2014**, *2014*, 698–707.

(73) Thomsen, J. M.; Sheehan, S. W.; Hashmi, S. M.; Campos, J.; Hintermair, U.; Crabtree, R. H.; Brudvig, G. W. Electrochemical activation of Cp* iridium complexes for electrode-driven water-oxidation catalysis. *J. Am. Chem. Soc.* **2014**, *136*, 13826–13834.

(74) Blakemore, J. D.; Schley, N. D.; Balcells, D.; Hull, J. F.; Olack, G. W.; Incarvito, C. D.; Eisenstein, O.; Brudvig, G. W.; Crabtree, R. H. Half-sandwich iridium complexes for homogeneous water-oxidation catalysis. *J. Am. Chem. Soc.* **2010**, *132*, 16017–16029.

(75) Oh, S.; Gallagher, J. R.; Miller, J. T.; Surendranath, Y. Graphite-Conjugated Rhenium Catalysts for Carbon Dioxide Reduction. *J. Am. Chem. Soc.* **2016**, *138*, 1820–1823.

(76) Jackson, M. N.; Oh, S.; Kaminsky, C. J.; Chu, S. B.; Zhang, G.; Miller, J. T.; Surendranath, Y. Strong Electronic Coupling of Molecular Sites to Graphitic Electrodes via Pyrazine Conjugation. *J. Am. Chem. Soc.* **2018**, *140*, 1004–1010.

(77) Jackson, M. N.; Surendranath, Y. Molecular Control of Heterogeneous Electrocatalysis through Graphite Conjugation. *Acc. Chem. Res.* **2019**, *52*, 3432–3441.

(78) Jackson, M. N.; Kaminsky, C. J.; Oh, S.; Melville, J. F.; Surendranath, Y. Graphite Conjugation Eliminates Redox Intermediates in Molecular Electrocatalysis. *J. Am. Chem. Soc.* **2019**, *141*, 14160–14167.

(79) Nie, W.; Tarnopol, D. E.; McCrory, C. C. The effect of extended conjugation on electrocatalytic CO2 reduction by molecular catalysts and macromolecular structures. *Curr. Opin. Electrochem.* **2021**, *28*, 100716.

(80) Rossmeisl, J.; Logadottir, A.; Nørskov, J. K. Electrolysis of water on (oxidized) metal surfaces. *Chem. Phys.* **2005**, *319*, 178–184.

(81) Ikeda, K.; Hori, Y.; Mahyuddin, M. H.; Shiota, Y.; Staykov, A.; Matsumoto, T.; Yoshizawa, K.; Ogo, S. Dual Catalytic Cycle of H2 and H2O Oxidations by a Half-Sandwich Iridium Complex: A Theoretical Study. *Inorg. Chem.* **2019**, *58*, 7274–7284.

(82) Vyas, V. S.; Vishwakarma, M.; Moudrakovski, I.; Haase, F.; Savasci, G.; Ochsenfeld, C.; Spatz, J. P.; Lotsch, B. V. Exploiting Noncovalent Interactions in an Imine-Based Covalent Organic Framework for Quercetin Delivery. *Adv. Mater.* **2016**, *28*, 8749–8754.

(83) Hosseini, P.; Rodríguez-Camargo, A.; Jiang, Y.; Zhang, S.; Scheu, C.; Yao, L.; Lotsch, B. V.; Tschulik, K. Shedding Light on the Active Species in a Cobalt-Based Covalent Organic Framework for the Electrochemical Oxygen Evolution Reaction. *Adv. Sci.* **2024**, e2413555.

(84) Bi, S.; Yang, C.; Zhang, W.; Xu, J.; Liu, L.; Wu, D.; Wang, X.; Han, Y.; Liang, Q.; Zhang, F. Two-dimensional semiconducting covalent organic frameworks via condensation at arylmethyl carbon atoms. *Nat. Commun.* **2019**, *10*, 2467.

(85) Jin, E.; Lan, Z.; Jiang, Q.; Geng, K.; Li, G.; Wang, X.; Jiang, D. 2D sp2 Carbon-Conjugated Covalent Organic Frameworks for Photocatalytic Hydrogen Production from Water. *Chem* **2019**, *5*, 1632–1647.

(86) Xu, J.; Yang, C.; Bi, S.; Wang, W.; He, Y.; Wu, D.; Liang, Q.; Wang, X.; Zhang, F. Vinylene-Linked Covalent Organic Frameworks (COFs) with Symmetry-Tuned Polarity and Photocatalytic Activity. *Angew. Chem. Int. Ed.* **2020**, *59*, 23845–23853.

(87) Chai, S.; Chen, X.; Zhang, X.; Fang, Y.; Sprick, R. S.; Chen, X. Rational design of covalent organic frameworks for efficient photocatalytic hydrogen peroxide production. *Environ. Sci. Nano* **2022**, *9*, 2464–2469.

(88) Yang, Y.; Chu, X.; Zhang, H.-Y.; Zhang, R.; Liu, Y.-H.; Zhang, F.-M.; Lu, M.; Yang, Z.-D.; Lan, Y.-Q. Engineering β-ketoamine covalent organic frameworks for photocatalytic overall water splitting. *Nat. Commun.* **2023**, *14*, 593.

(89) He, Y.; Liu, G.; Liu, Z.; Bi, J.; Yu, Y.; Li, L. Photoinduced Hydration Boosts O2 Evolution on Co-Chelating Covalent Organic Framework. *ACS Energy Lett.* **2023**, *8*, 1857–1863.

(90) Borse, R. A.; Tan, Y.-X.; Lin, J.; Zhou, E.; Hui, Y.; Yuan, D.; Wang, Y. Coupling Electron Transfer and Redox Site in Boranil Covalent Organic Framework Toward Boosting Photocatalytic Water Oxidation. *Angew. Chem. Int. Ed.* **2024**, *63*, e202318136.

(91) Zhou, E.; Zhang, X.; Zhu, L.; Chai, E.; Chen, J.; Li, J.; Yuan, D.; Kang, L.; Sun, Q.; Wang, Y. Ultrathin covalent organic framework nanosheets for enhanced photocatalytic water oxidation. *Sci. Adv.* **2024**, *10*, eadk8564.

(92) Xiao, Z.; Wu, H.; Jiao, L.; Zhang, X.; Wang, Y. A dual-functional metalloporphyrin-fluorenone covalent organic framework for solar hydrogen and oxygen production. *J. Mater. Chem. A* **2024**, *12*, 7515–7521.

(93) Li, X.; Yang, Q.; Yuan, Y.; Shama, Y.; Yan, H. Inhibiting Photo-Oxidation and Enhancing Visible-Light-Driven Photocatalytic Water Oxidation over Covalent Organic Frameworks Through the Coordination of Cobalt with Bipyridine. *Small* **2024**, e2401168.

(94) Yu, M.; Li, Q.; Li, L.; Yu, Y. Dual Cocatalysts on Covalent Organic Framework as Charge Transfer Mediator Facilitating Photocatalytic Water Oxidation. *ChemCatChem* **2024**, *16*, e202401154.

(95) Xie, S.; Liu, R.; Liu, N.; Xu, H.; Chen, X.; Wang, X.; Jiang, D. Vertically Expanded Covalent Organic Frameworks for Photocatalytic Water Oxidation into Oxygen. *Angew. Chem. Int. Ed.* **2025**, *64*, e202416771.

(96) Chen, L.; Wang, L.; Wan, Y.; Zhang, Y.; Qi, Z.; Wu, X.; Xu, H. Acetylene and Diacetylene Functionalized Covalent Triazine Frameworks as Metal-Free Photocatalysts for Hydrogen

Peroxide Production:A New Two-Electron Water Oxidation Pathway. *Adv. Mater.* **2020**, *32*, e1904433.

(97) Xie, J.; Shevlin, S. A.; Ruan, Q.; Moniz, S. J. A.; Liu, Y.; Liu, X.; Li, Y.; Lau, C. C.; Guo, Z. X.; Tang, J. Efficient visible light-driven water oxidation and proton reduction by an ordered covalent triazine-based framework. *Energy Environ. Sci.* **2018**, *11*, 1617–1624.

(98) Sprick, R. S.; Chen, Z.; Cowan, A. J.; Bai, Y.; Aitchison, C. M.; Fang, Y.; Zwijnenburg, M. A.; Cooper, A. I.; Wang, X. Water Oxidation with Cobalt-Loaded Linear Conjugated Polymer Photocatalysts. *Angew. Chem. Int. Ed.* **2020**, *59*, 18695–18700.

(99) Frame, F. A.; Townsend, T. K.; Chamousis, R. L.; Sabio, E. M.; Dittrich, T.; Browning, N. D.; Osterloh, F. E. Photocatalytic water oxidation with nonsensitized IrO2 nanocrystals under visible and UV light. *J. Am. Chem. Soc.* **2011**, *133*, 7264–7267.
